# Supplementary material for: Fecal DNA metabarcoding helps characterize the Canada jay’s diet and confirms its reliance on stored food for winter survival and breeding
Source: PLoS One. 2024 Apr 24;19(4):e0300583. doi: 10.1371/journal.pone.0300583 (PMC11042713; doi:10.1371/journal.pone.0300583)
Supplement: S1 Table — The date and location the observation was made is included for each observation in addition to the identification of the food item being consumed. We also summarize additional information related to who made the record, what method was used to make the observation (“DO”–direct observation, “SC”–stomach content analysis, “FS”–metabarcoding of fecal samples), the age of the individual which consumed a food item (e.g., adult, juvenile, or nestling), and which of our four diet groups the observation belongs in (“A”–arthropod, “P”–“plants”, “V”–vertebrate tissue). Finally, for observations made during the winter and spring, we describe whether a food item was “likely-cached”, “likely fresh”, or if it could be either cached or fresh. Observations labeled with “ROM” represent stomach samples from the Royal Ontario Museum collection. (DOCX) [file pone.0300583.s001.docx]

| **Year** | **Month** | **Ordinal Date** | **Location** | **Food item(s)** | **Class** | **Order** | **Observer** | **Method** | **Age** | **Food type** | **Caching Designation** |
| --- | --- | --- | --- | --- | --- | --- | --- | --- | --- | --- | --- |
| 1970 | April | 103 | La Vérendrye, Québec | Flying "midge" | Insecta | Diptera | DS, unpublished | DO | Ad | A |  |
| 1982 | April | 104 | Algonquin, Ontario | Winter ticks (*Dermacentor albipictus*) | Arachnida | Ixodida | Addison et al. 1989 | DO | Ad | A |  |
| 2023 | April | 94 | Pattee Canyon Recreation Area, Montana | Winter ticks (*Dermacentor albipictus*) | Arachnida | Ixodida | Hendricks 2023 | DO | Ad | A |  |
| 1916 | April | 111 | Oregon? | Stoneflies (newly hatched) | Insecta | Plecoptera | Rathbun in Bent 1946 | DO | Ad | A |  |
| 1945 | April | 119 | Ontario | "Small moustached mosquitos" | Insecta | Diptera | Lawrence 1947 | DO | Ad | A |  |
| 1981 | May | 122 | Algonquin, Ontario | Winter ticks (*Dermacentor albipictus*) | Arachnida | Ixodida | Addison et al. 1989 | DO | Ad | A |  |
| 1963 | July | 201 | Anticosti | Maggots | Insecta | Diptera | Ouellet 1970 | DO | Ad | A |  |
| 1977 | August | 239 | Algonquin, Ontario | Large flying beetle | Insecta | Coleoptera | G. Thorn, pers. comm. | DO | Ad | A |  |
| 1977 | August | 241 | Algonquin, Ontario | Winged ants emerging from old railway embankment | Insecta | Hymenoptera | W. J. Crins, pers. comm. | DO | Ad | A |  |
| 1991 | August | 241 | Algonquin, Ontario | Wingless ants at edge of the road | Insecta | Hymenoptera | M. Runtz, P. Smith, pers. comm. | DO | Ad | A |  |
| 1938 | September | 263 | James Bay | Wasps and/or larvae and/or pupae | Insecta | Hymenoptera | Lewis 1939 | DO | Ad | A |  |
| 2004 | September | 267 | Algonquin, Ontario | Black-tipped Darner (*Aeshna tuberculifera*) | Insecta | Odonata | Earley 2005 | DO | Ad | A |  |
| 1968 | October | 280 | La Vérendrye, Québec | Insects in grass by roadside | Insecta | Unknown | DS, unpublished | DO | Ad | A |  |
| Not given | October | 288 | Maine | Grasshoppers ("apparently") | Insecta | Orthoptera | Brewster 1937 | DO | Ad | A |  |
| Not given | November | 315 | Ontario | "Moth-like insects" | Insecta | Lepidoptera | Lawrence 1968 | DO | Ad | A |  |
| Not given | Fall? | 311 | New Mexico | "Insects, especially grasshoppers and caterpillars" | Insecta | Unknown | Bailey 1928 in Bent 1946 | DO | Ad | A |  |
| Not given | Fall? | 311 | New Mexico | "Insects, especially grasshoppers and caterpillars" | Insecta | Orthoptera | Bailey 1928 in Bent 1946 | DO | Ad | A |  |
| Not given | Fall? | 311 | New Mexico | "Insects, especially grasshoppers and caterpillars" | Insecta | Lepidoptera | Bailey 1928 in Bent 1946 | DO | Ad | A |  |
| Not given | Fall | 311 | Not given | Grasshoppers | Insecta | Orthoptera | Rutter 1969 | DO | Ad | A |  |
| Not given | Not given |  | Minnesota | Grubs from floating logs | Insecta | Unknown | Warren 1899 | DO | Ad | A |  |
| 1968 | March | 66 | La Vérendrye, Québec | White Spruce (*Picea glauca*) seeds | Coniferopsida | Pinales | DS, unpublished | DO | Ad | P |  |
| 1984 | March | 86 | Algonquin, Ontario | *Phibalis furfuracea* (Roth.) wallr - ascomycete | Leotiomycetes | Helotiales | Collected by DS. Identified by J. Ginus courtesy of Greg Thorn | DO | Ad | P |  |
| 1970 | April | 102 | La Vérendrye, Québec | Balsam fir (*Abies balsamea*) seeds | Coniferopsida | Pinales | DS, unpublished | DO | Ad | P |  |
| 2020 | July | 187 | Algonquin, Ontario | Unidentified bolete (fruiting body) | Agaricomycetes | Boletales | Fuirst et al. 2022 | DO | Ad | P |  |
| 2020 | July | 190 | Algonquin, Ontario | Craterellus sp. (fruiting body) | Agaricomycetes | Cantharellales | Fuirst et al. 2022 | DO | Ad | P |  |
| 1977 | July | 207 | Washington State | Slime mold (Fuligo septica) | Myxogastria | Physarales | Sutherland and Crawford 1979 | DO | Ad | P |  |
| 2020 | July | 211 | Vancouver Island, BC | Slime mold (Fuligo septica) | Myxogastria | Physarales | DS, unpublished (photo) | DO | Ad | P |  |
| 2021 | July | 211 | Algonquin, Ontario | *Amanita muscaria* - mushroom | Agaricomycetes | Agaricales | Fuirst et al. 2022 | DO | Ad | P |  |
| 1961 | September | 247 | Québec | Blueberries (Vaccinium sp) | Magnoliopsida | Ericales | Ouellet 1970 | DO | Ad | P |  |
| 1980 | September | 258 | Mount Revelstoke, BC | *Amanita pantherina* - mushroom | Agaricomycetes | Agaricales | Greg Thorn, pers. comm. | DO | Ad | P |  |
| 2021 | September | 260 | Montana | *Symphoricarpos albus* | Magnoliopsida | Ericales | Hendricks and Pagano 2023 | DO | A | P |  |
| 1979 | September | 261 | Algonquin, Ontario | *Amanita muscaria* - mushroom | Agaricomycetes | Agaricales | DS, unpublished | DO | Ad | P |  |
| Not given | September & October | 273 | Alaska | Blueberries | Magnoliopsida | Ericales | Grinnell in Bent 1946 | DO | Ad | P |  |
| 1968 | October | 280 | Québec | Undigested vegetal material in bear dung | Unknown | Unknown | Ouellet 1970 | DO | Ad | P |  |
| 1973 | October | 290 | Cook Co. Minnesota | Mountain-ash berries (Sorbus americana) | Magnoliopsida | Rosales | Olyphant 1976 | DO | Ad | P |  |
| Not given | "late fall" |  | Algonquin, Ontario | Frozen chokecherries (Prunus virginiana) | Magnoliopsida | Rosales | Rutter 1969 | DO | Ad | P |  |
| Not given | Fall? | 311 | New Mexico | "Wild fruits including elderberry…" | Magnoliopsida | Dipsacales | Bailey 1928 in Bent 1946 | DO | Ad | P |  |
| Not given | Fall? | 311 | New Mexico | "… sumac …" | Magnoliopsida | Sapindales | Bailey 1928 in Bent 1946 | DO | Ad | P |  |
| Not given | Fall? | 311 | New Mexico | "... and viburnum…" | Magnoliopsida | Dipsacales | Bailey 1928 in Bent 1946 | DO | Ad | P |  |
| Not given | Fall? | 311 | New Mexico | "… also scattered grain in corrals" | Magnoliopsida | Poales | Bailey 1928 in Bent 1946 | DO | Ad | P |  |
| Not given | Fall? | 311 | Not given | "Toadstool" mushroom | Unknown | Unknown | Bailey 1904 in Bent 1946 | DO | Ad | P |  |
| Not given | Fall? | 311 | Not given | "2 Persimon seeds" | Magnoliopsida | Ericales | Audubon 1841 | DO | Ad | P |  |
| Not given | Late fall | 330 | Ontario | Frozen chokecherries (Prunus virginiana) | Magnoliopsida | Rosales | R. J. Rutter, pers. comm. | DO | Ad | P |  |
| 1984 | January | 21 | Lake County, Minnesota | Shrew or small rodent | Mammalia | Unknown | Lesher and Lesher, 1984 | DO | Ad | VL |  |
| 1973 | January | 27 | Algonquin, Ontario | Flesh of Snowshoe hare (Lepus americanus) | Mammalia | Lagomorpha | Paul Catling, pers. comm. | DO | Ad | VC |  |
| 1967 | February | 50 | La Vérendrye, Québec | Boreal Chickadee (Poecile hudsonicus) | Aves | Passeriformes | Strickland 1969 | DO | Ad | VL |  |
| 1970 | February | 51 | La Vérendrye, Québec | Common Redpoll (Acanthis flammea) | Aves | Passeriformes | DS, unpublished | DO | Ad | VL |  |
| 2004 | February | 56 | Algonquin, Ontario | Black-capped Chickadee (Poecile atricapillus) | Aves | Passeriformes | Tozer and Allen 2004 | DO | Ad | VL |  |
| 2018 | February | 85 | Algonquin, Ontario | 3-5 day-old crossbill nestling Loxia sp. | Aves | Passeriformes | A. Sutton and N. Freeman, pers. comm. | DO | Ad | VL |  |
| 1967 | March | 60 | La Vérendrye, Québec | Boreal Chickadee (Poecile hudsonicus) | Aves | Passeriformes | Strickland 1969 | DO | Ad | VL |  |
| 1985 | March | 62 | Algonquin, Ontario | Apparently already dead Golden-crowned kinglet (Regulus satrapa) | Aves | Passeriformes | R.G. Tozer, pers. comm. | DO | Ad | VC |  |
| 1967 | March | 65 | La Vérendrye, Québec | Boreal Chickadee (Poecile hudsonicus) | Aves | Passeriformes | Strickland 1969 | DO | Ad | VL |  |
| 1970 | March | 65 | La Vérendrye, Québec | Leg of small bird | Aves | Passeriformes | DS, unpublished | DO | Ad | VC |  |
| 1967 | March | 69 | La Vérendrye, Québec | Spruce grouse carcass (Canachites canadensis) | Aves | Galliformes | DS, unpublished | DO | Ad | VC |  |
| 1968 | March | 70 | La Vérendrye, Québec | Dead mouse (Clethrionomys gapperi?) | Mammalia | Rodentia | DS, unpublished | DO | Ad | VC |  |
| 1967 | March | 84 | La Vérendrye, Québec | Intestines and fecal matter of Ruffed Grouse carcass (Bonasa umbellus) | Aves | Galliformes | DS, unpublished | DO | Ad | VC |  |
| 1968 | March | 87 | La Vérendrye, Québec | Boreal Chickadee (Poecile hudsonicus) | Aves | Passeriformes | Strickland 1969 | DO | Ad | VL |  |
|  | March | 90 | Red Lake, Ontario | Unidentified mouse or vole | Mammalia | Rodentia | Gilmore 1996 | DO | Ad | VL |  |
| 1967 | April | 112 | La Vérendrye, Québec | Lynx carcass (Lynx lynx) | Mammalia | Carnivora | DS, unpublished | DO | Ad | VC |  |
| 2020 | May | 135 | Algonquin, Ontario | Live American toad (Anaxyrus americanus) | Amphibia | Anura | Fuirst et al. 2022 | DO | Ad | VL |  |
| 2020 | May | 140 | Algonquin, Ontario | Live Spotted Salamander (Ambystoma maculatum) | Amphibia | Caudata | Fuirst et al. 2022 | DO | Ad | VL |  |
| 2021 | May | 144 | Algonquin, Ontario | Spotted Salamander (Ambystoma maculatum) | Amphibia | Caudata | Fuirst et al. 2022 | DO | Ad | VL |  |
| 1974 | May | 149 | Algonquin, Ontario | Egg of Robin (Turdus migratorius) | Aves | Passeriformes | R.G. Tozer, pers. comm. | DO | Ad | VE |  |
| 2021 | May | 151 | Algonquin, Ontario | Red-backed Salamander (Plethodon cinereus) | Amphibia | Caudata | Fuirst et al. 2022 | DO | Ad | VL |  |
|  | June | 156 | Whitefish Point, Michigan | Black-capped Chickadee (Poecile atricapillus) | Aves | Passeriformes | Pike 1978 | DO | Ad | VL |  |
| 2021 | June | 158 | Algonquin, Ontario | Chestnut-sided Warbler (Setophaga pensylvanica) | Aves | Passeriformes | Fuirst et al. 2022 | DO | Ad | VL |  |
| 1969 | June | 159 | Québec | Egg, probably of Swainson's thrush (Catharus ustulatus) | Aves | Passeriformes | Ouellet 1970 | DO | Ad | VE |  |
| 1969 | June | 161 | Québec | White-throated Sparrow nestling (Zonotrichia albicollis) | Aves | Passeriformes | Ouellet 1970 | DO | Ad | VL |  |
| 1973 | June | 161 | Québec | Swainson's thrush nestling (Catharus ustulatus) | Aves | Passeriformes | Ouellet 1970 | DO | Ad | VL |  |
| Not given | June | 163 | Ungava, Québec | Dead "Round Whitefish" (Prosopium sp) | Actinopterygii | Salmoniformes | Harper 1958 | DO | Ad | VC |  |
| 1969 | June | 163 | Québec | Fat of recently skinned bear carcass (Ursus americanus) | Mammalia | Carnivora | Ouellet 1970 | DO | Ad | VC |  |
| Not given | June | 164 | Labrador | Dead Northern Lemming mouse (Synaptomys borealis) | Mammalia | Rodentia | Harper 1953 | DO | Ad | VC |  |
| 1969 | June | 169 | Québec | Small bird, probably fledgling Hermit thrush (Catharus guttatus) | Aves | Passeriformes | Ouellet 1970 | DO | Ad | VL |  |
| 2021 | June | 170 | Algonquin, Ontario | White-winged crossbill (Loxia leucoptera) | Aves | Passeriformes | Fuirst et al. 2022 | DO | Ad | VL |  |
| 1967 | June | 174 | Québec | Vole (Clethrionomys gapperi) | Mammalia | Rodentia | Ouellet 1970 | DO | Ad | VC |  |
| 1968 | June | 175 | La Vérendrye, Québec | Nestling possibly Common grackle (Quiscalus quiscula) | Aves | Passeriformes | DS, unpublished | DO | Ad | VL |  |
| 1958 | June | 177 | Québec | Egg of Robin (Turdus migratorius) | Aves | Passeriformes | Ouellet 1970 | DO | Ad | VE |  |
| 1994 | June? | 181 | Colorado | Cassin's Finch (Haemoorhous cassinii) nestlings | Aves | Passeriformes | Kingery and Kingery 1995 | DO | Ad | VE |  |
| Not given | June or July | 181 | Bay of Fundy | Eggs of Crow (Corvus brachyrynchos) | Aves | Passeriformes | Audubon 1840 | DO | Ad | VE |  |
| 1959 | June | 181 | Clearwater Co. Minnesota | Nursing Hoary Bat (Lasiurus cinereus) | Mammalia | Chiroptera | Macior 1959 | DO | Ad | VL |  |
| Not given | June or July | 181 | New England | Junco nestlings (Junco hyemalis) from 4 nests | Aves | Passeriformes | Samuels in Bendire 1895 | DO | Ad | VL |  |
| Not given | June or July | 181 | Oregon | Junco nestlings | Aves | Passeriformes | Longmire in Jewett et al. 1953 | DO | Ad | VL |  |
| 2021 | July | 182 | Algonquin, Ontario | Black-capped Chickadee (Poecile atricapillus) | Aves | Passeriformes | Fuirst et al. 2022 | DO | Ad | VL |  |
| 1963 | July | 183 | Anticosti | Deer carcass (Odocoileus virginianus) | Mammalia | Artiodactyla | Ouellet 1970 | DO | Ad | VC |  |
| 1939 | July | 186 | British Columbia | Ruby-crowned kinglet nestlings (Regulus calendula) | Aves | Passeriformes | Munro in Bent 1946 | DO | Ad | VL |  |
| 1960 | July | 187 | Labrador Coast | Egg, probably of White-crowned sparrow (Zonotrichia leucophrys) | Aves | Passeriformes | Ouellet 1970 | DO | Ad | VE |  |
| 1960 | July | 192 | Labrador Coast | Small bird presumed to be chick of Spruce grouse (Canachites canadensis) | Aves | Galliformes | Ouellet 1970 | DO | Ad | VL |  |
| 1957 | July | 193 | New Brunswick | Lincoln's sparrow nestling (Melospiza lincolnii) | Aves | Passeriformes | Ouellet 1970 | DO | Ad | VL |  |
| 1990 | July | 200 | Vermont | Magnolia Warbler juvenile (Setophaga magnolia) | Aves | Passeriformes | Barnard 1996 | DO | Ad | VL |  |
| 1963 | July | 201 | Anticosti | Deer carcass (Odocoileus virginianus) | Mammalia | Artiodactyla | Ouellet 1970 | DO | Ad | VC |  |
| 2020 | July | 201 | Algonquin, Ontario | Magnolia warbler adult (Setpophaga magnolia) | Aves | Passeriformes | Fuirst et al. 2022 | DO | Ad | VL |  |
| 1961 | July | 209 | Québec | Magnolia warbler fledgling (Dendroica magnolia) | Aves | Passeriformes | Ouellet 1970 | DO | Ad | VL |  |
| Not given | "Midsummer" | 212 | British Columbia | Audubon's warbler nestlings (Dendroica coronata auduboni) | Aves | Passeriformes | Munro in Bent 1946 | DO | Ad | VL |  |
| 1963 | August | 224 | Anticosti | Red fox carcass (Vulpes vulpes) | Mammalia | Carnivora | Ouellet 1970 | DO | Ad | VC |  |
| 1957 | August | 231 | New Brunswick | Ruffed grouse carcass (Bonasa umbellus) | Aves | Galliformes | Ouellet 1970 | DO | Ad | VC |  |
| Not given | August - December | 232 | Northern Manitoba | Carcasses of caribou (Rangifer tarandus) | Mammalia | Artiodactyla | Harper 1958 | DO | Ad | VC |  |
| 1960 | August | 235 | Labrador Coast | Carcass of seal and fish offal | Mammalia | Carnivora | Ouellet 1970 | DO | Ad | VC |  |
| 2016 | August | 239 | Algonquin, Ontario | Long-tailed Shrew (Sorex sp.) | Mammalia | Soricomorpha | Ann Brokelman, pers. comm. (photos) | DO | Ad | V? |  |
| 2016 | August | 239 | Algonquin, Ontario | Hairy-tailed Mole (Parascalops breweri) | Mammalia | Soricomorpha | Ann Brokelman pers. comm. (photos) | DO | Ad | V? |  |
| 1972 | September | 255 | Huntsville, Ontario | One live Red-bellied snake (Storeria occipitomaculata) | Reptilia | Squamata | R. Helselfide R. J. Rutter, pers. comm | DO | Ad | VL |  |
| Not given | September | 258 | Ungava, Québec | Dead vole (Microtus) | Mammalia | Rodentia | Harper 1953 | DO | Ad | VC |  |
| 1978 | September | 268 | Albany Co. Wyoming | Tadpoles of boreal toad, Bufo boreas | Amphibia | Anura | Beiswenger 1981 | DO | Ad | VC |  |
| 2003 | October | 276 | Crater Lake N.P. Oregon | Larval Long-toed Salamanders (Ambystoma macrodactylum) | Amphibia | Caudata | Murray et al. 2005 | DO | Ad | VL |  |
| 1967 | October | 280 | La Vérendrye, Québec | Moose flesh (Alces alces) | Mammalia | Artiodactyla | DS, unpublished | DO | Ad | VC |  |
| 1974 | October | 285 | Algonquin, Ontario | Leopard (?) frog (Rana pipiens) | Amphibia | Anura | H. J. Hawkins, R. G. Tozer, pers. comm. | DO | Ad | VL |  |
| 1974 | October | 287 | Québec | Dead fish | Pisces | Unknown | Ouellet 1970 | DO | Ad | VC |  |
| 1968 | October | 289 | Québec | Pine Grosbeak (Pinicola enucleator) | Aves | Passeriformes | Ouellet 1970 | DO | Ad | VC |  |
| 1968 | October | 289 | Québec | Pine siskin (Spinus pinus) | Aves | Passeriformes | Ouellet 1970 | DO | Ad | VC |  |
| 1968 | October | 289 | Québec | Evening Grosbeak (Hesperiphona vespertina) | Aves | Passeriformes | Ouellet 1970 | DO | Ad | VC |  |
| 1968 | October | 289 | Québec | White-winged Crossbill (Loxia leucoptera) | Aves | Passeriformes | Ouellet 1970 | DO | Ad | VC |  |
| 1968 | October | 289 | Québec | Gray jay (Perisoreus canadensis) | Aves | Passeriformes | Ouellet 1970 | DO | Ad | VC |  |
| 1933 | October | 292 | Manitoba | Ruffed grouse carcass (Bonasa umbellus) | Aves | Galliformes | ROM 69578 | DO | Ad | VC |  |
| 1973 | October | 298 | Alberta | Two boreal deer mice (Peromyscus maniculatus borealis) | Mammalia | Rodentia | Gill 1974 | DO | Ad | VC |  |
| 1973 | October | 298 | Alberta | Deer mice (Peromyscus maniculatus borealis) - 2 | Mammalia | Rodentia | Gill 1974 | DO | Ad | VL |  |
| Not given | Not given | 334 | Maine | Marten carcasses (Martes americana) | Mammalia | Carnivora | Hardy in Bendire 1895 | DO | Ad | VC |  |
| Not given | Not given | 334 | Maine | Skinned beaver carcass (Castor canadensis) | Mammalia | Rodentia | Hardy in Bendire 1895 | DO | Ad | VC |  |
| Not given | Not given | 334 | Maine | "Duck" breast | Aves | Anseriformes | Hardy in Bendire 1895 | DO | Ad | VC |  |
| Not given | Not given | 334 | Labrabor | Ptarmigan carcasses (Lagopus sp) | Aves | Galliformes | Clement in Todd 1964 | DO | Ad | VC |  |
| Not given | Winter | 334 | Algonquin, Ontario | Deer carcasses (Odocoileus virginanus) | Mammalia | Artiodactyla | Rutter 1969 | DO | Ad | VC |  |
| 1967 | December | 335 | La Vérendrye, Québec | Crossbills (Loxia curvirostra and leucoptera) | Aves | Passeriformes | DS, unpublished | DO | Ad | VC |  |
| 1970 | April | 105 | La Vérendrye, Québec | Food mass containing a segmented insect larva about 2.5 cm long, and dull, dark brown in colour | Insecta | Unknown | DS, unpublished | DO | Ne | A |  |
| 1970 | April | 103 | La Vérendrye, Québec | Flesh judged to be old from its dull red color; food mass also included white portions presumed to be fat | (Vertebrate) | Unknown | DS, unpublished | DO | Ne | V |  |
| 2015 | April | 109 | Algonquin, Ontario | Protoboarnia porcelaria (Porcelain gray moth) | Insecta | Lepidoptera | This study | FS | Ne | A | Either Possible |
| 2015 | April | 109 | Algonquin, Ontario | Cicurina arcuata (Meshweaver spider) | Arachnida | Araneae | This study | FS | Ne | A | Likely Fresh |
| 2015 | April | 109 | Algonquin, Ontario | Dryocampa rubicunda (Rosy maple moth) | Insecta | Lepidoptera | This study | FS | Ne | A | Likely Cached |
| 2015 | April | 109 | Algonquin, Ontario | Xysticus elegans Elegant crab spider) | Arachnida | Araneae | This study | FS | Ne | A | Likely Cached |
| 2015 | April | 109 | Algonquin, Ontario | Araneus marmoreus (Orbweaver spider) | Arachnida | Araneae | This study | FS | Ne | A | Likely Cached |
| 2015 | April | 109 | Algonquin, Ontario | Lithophane lanei (Pinion moth) | Insecta | Lepidoptera | This study | FS | Ne | A | Likely Fresh |
| 2015 | April | 109 | Algonquin, Ontario | Lithophane baileyi (Bailey's pinion moth) | Insecta | Lepidoptera | This study | FS | Ne | A | Likely Fresh |
| 2015 | April | 109 | Algonquin, Ontario | Deltote bellicula (Bog Lithacodia moth | Insecta | Lepidoptera | This study | FS | Ne | A | Likely Fresh |
| 2015 | April | 109 | Algonquin, Ontario | Pityophantes subarcticus (Sheetweb spider) | Arachnida | Araneae | This study | FS | Ne | A | Either Possible |
| 2015 | April | 109 | Algonquin, Ontario | Dolomedes striatus (Fishing spider) | Arachnida | Araneae | This study | FS | Ne | A | Likely Fresh |
| 2015 | April | 109 | Algonquin, Ontario | Phragmatobia assimilans (Dusky red tiger moth) | Insecta | Lepidoptera | This study | FS | Ne | A | Either Possible |
| 2015 | April | 109 | Algonquin, Ontario | Lithophane petulca (Wanton pinion moth) | Insecta | Lepidoptera | This study | FS | Ne | A | Likely Fresh |
| 2015 | April | 109 | Algonquin, Ontario | Phlogophora periculosa (Brown angle shades moth) | Insecta | Lepidoptera | This study | FS | Ne | A | Likely Cached |
| 2015 | April | 109 | Algonquin, Ontario | Nabis rufusculus (Damsel bug) | Insecta | Hemiptera | This study | FS | Ne | A | Either Possible |
| 2015 | April | 109 | Algonquin, Ontario | Megaselia arcticae (Scuttle fly) | Insecta | Diptera | This study | FS | Ne | A | Unknown |
| 2015 | April | 109 | Algonquin, Ontario | Lithophane pexata (Plush-naped pinion moth) | Insecta | Lepidoptera | This study | FS | Ne | A | Likely Cached |
| 2015 | April | 109 | Algonquin, Ontario | Arctia caja (Garden tiger moth) | Insecta | Lepidoptera | This study | FS | Ne | A | Either Possible |
| 2015 | April | 109 | Algonquin, Ontario | Spodolepis substriataria (Geometrid moth) | Insecta | Lepidoptera | This study | FS | Ne | A | Likely Fresh |
| 2015 | April | 109 | Algonquin, Ontario | Cicurina placida (Meshweaver spider) | Arachnida | Araneae | This study | FS | Ne | A | Unknown |
| 2015 | April | 109 | Algonquin, Ontario | Cratyna longispina (Black fungus gnat) | Insecta | Diptera | This study | FS | Ne | A | not applicable |
| 2015 | April | 109 | Algonquin, Ontario | Callobius bennetti (Hackledmesh weaver spider) | Arachnida | Araneae | This study | FS | Ne | A | Either Possible |
| 2015 | April | 109 | Algonquin, Ontario | Tenuiphantes zebra (Sheetweb spider) | Arachnida | Araneae | This study | FS | Ne | A | Likely Fresh |
| 2015 | April | 109 | Algonquin, Ontario | Alopecosa aculeata (Wolf spider) | Arachnida | Araneae | This study | FS | Ne | A | Either Possible |
| 2016 | April | 109 | Algonquin, Ontario | Orthocladiinae (Midge) | Insecta | Diptera | This study | FS | Ne | A | Likely Fresh |
| 2017 | April | 120 | Algonquin, Ontario | Cladara limitaria (Mottled gray carpet moth) | Insecta | Lepidoptera | This study | FS | Ne | A | Unknown |
| 2017 | April | 120 | Algonquin, Ontario | Callobius bennetti (Hackledmesh weaver spider) | Arachnida | Araneae | This study | FS | Ne | A | Either Possible |
| 2017 | April | 120 | Algonquin, Ontario | Semioscopis inornata (Dull flat-bodied moth) | Insecta | Lepidoptera | This study | FS | Ne | A | Likely Fresh |
| 2017 | April | 120 | Algonquin, Ontario | Cicurina arcuata (Meshweaver spider) | Arachnida | Araneae | This study | FS | Ne | A | Likely Fresh |
| 2017 | April | 120 | Algonquin, Ontario | Clerinae (Checkered beetle) | Insecta | Coleoptera | This study | FS | Ne | A | Unknown |
| 2017 | April | 120 | Algonquin, Ontario | Eupithecia annulata (Larch pug moth) | Insecta | Lepidoptera | This study | FS | Ne | A | Likely Fresh |
| 2017 | April | 120 | Algonquin, Ontario | Calopus angustus (False blister beetle) | Insecta | Coleoptera | This study | FS | Ne | A | Likely Fresh |
| 2017 | April | 120 | Algonquin, Ontario | Anthophylax attenuatus (Mottled long-horned beetle) | Insecta | Coleoptera | This study | FS | Ne | A | Likely Fresh |
| 2017 | April | 120 | Algonquin, Ontario | Eristalis dimidiata (Hover fly) | Insecta | Diptera | This study | FS | Ne | A | Likely Fresh |
| 2017 | April | 120 | Algonquin, Ontario | Syndemis afflictana (Gray leafroller) | Insecta | Lepidoptera | This study | FS | Ne | A | Likely Fresh |
| 2017 | April | 120 | Algonquin, Ontario | Protoboarmia porcelaria (Porcelain gray moth) | Insecta | Lepidoptera | This study | FS | Ne | A | Likely Fresh |
| 2017 | April | 120 | Algonquin, Ontario | Gnorimoschema (Twirler moth) | Insecta | Lepidoptera | This study | FS | Ne | A | Unknown |
| 2017 | April | 120 | Algonquin, Ontario | Zanclognatha jacchusalis (Wavy-lined zanclognatha moth) | Insecta | Lepidoptera | This study | FS | Ne | A | Either Possible |
| 2017 | April | 120 | Algonquin, Ontario | Lepidoptera (butterfly or moth) | Insecta | Lepidoptera | This study | FS | Ne | A | Unknown |
| 2017 | April | 120 | Algonquin, Ontario | Parajulidae (Millipede) | Diplopoda | Julida | This study | FS | Ne | A | Unknown |
| 2017 | April | 120 | Algonquin, Ontario | Agroeca ornata (Liocranid sac spider) | Arachnida | Araneae | This study | FS | Ne | A | Likely Fresh |
| 2017 | April | 120 | Algonquin, Ontario | Agriotes collaris (Click beetle) | Insecta | Coleoptera | This study | FS | Ne | A | Likely Fresh |
| 2017 | April | 120 | Algonquin, Ontario | Ilybius gagates (Predacious diving-beetle) | Insecta | Coleoptera | This study | FS | Ne | A | Unknown |
| 2017 | April | 120 | Algonquin, Ontario | Criorhina nigriventris (Hover fly) | Insecta | Diptera | This study | FS | Ne | A | Likely Fresh |
| 2017 | April | 120 | Algonquin, Ontario | Araneus nordmanni (Orbweaver spider) | Arachnida | Araneae | This study | FS | Ne | A | Likely Cached |
| 2017 | April | 120 | Algonquin, Ontario | Protoboarmia porcelaria (Porcelain gray moth) | Insecta | Lepidoptera | This study | FS | Ne | A | Likely Fresh |
| 2017 | April | 120 | Algonquin, Ontario | Stigmaeidae | Arachnida | Trombidiformes | This study | FS | Ne | A | not applicable |
| 2017 | April | 120 | Algonquin, Ontario | Phragmatobia fuliginosa (Ruby tiger moth) | Insecta | Lepidoptera | This study | FS | Ne | A | Likely Fresh |
| 2017 | April | 120 | Algonquin, Ontario | Piratula insularis (Lonely wolf spider) | Arachnida | Araneae | This study | FS | Ne | A | Unknown |
| 2017 | April | 120 | Algonquin, Ontario | Pityophantes subarcticus (Sheetweb spider) | Arachnida | Araneae | This study | FS | Ne | A | Either Possible |
| 2017 | April | 120 | Algonquin, Ontario | Acilus mediatus (Predacious diving-beetle) | Insecta | Coleoptera | This study | FS | Ne | A | Likely Fresh |
| 2017 | April | 120 | Algonquin, Ontario | Acilus sylvanus (Predacious diving-beetle) | Insecta | Coleoptera | This study | FS | Ne | A | Likely Fresh |
| 2017 | April | 120 | Algonquin, Ontario | Piratula cantralli (Wolf spider) | Arachnida | Araneae | This study | FS | Ne | A | Unknown |
| 2017 | April | 120 | Algonquin, Ontario | Podisus serieventris (Predatory stink-bug) | Insecta | Hemiptera | This study | FS | Ne | A | Likely Cached |
| 2017 | April | 120 | Algonquin, Ontario | Cladara atroliturata (Scribbler moth) | Insecta | Lepidoptera | This study | FS | Ne | A | Likely Fresh |
| 2016 | May | 126 | Algonquin, Ontario | Callobius bennetti (Hackledmesh weaver spider) | Arachnida | Araneae | This study | FS | Ne | A | Either Possible |
| 2016 | May | 126 | Algonquin, Ontario | Tibellus maritimus (Slender crab spider) | Arachnida | Araneae | This study | FS | Ne | A | Likely Fresh |
| 2016 | May | 126 | Algonquin, Ontario | Anaplectoides pressus (Dappled dart moth) | Insecta | Lepidoptera | This study | FS | Ne | A | Either Possible |
| 2016 | May | 126 | Algonquin, Ontario | Ilybius gagates (Predacious diving-beetle) | Insecta | Coleoptera | This study | FS | Ne | A | Unknown |
| 2016 | May | 126 | Algonquin, Ontario | Phlogophora periculosa (Brown angle shades moth) | Insecta | Lepidoptera | This study | FS | Ne | A | Likely Cached |
| 2016 | May | 126 | Algonquin, Ontario | Orthosia revicta (Rusty white-sided moth) | Insecta | Lepidoptera | This study | FS | Ne | A | Likely Fresh |
| 2016 | May | 126 | Algonquin, Ontario | Xestia sp. (Spotted cutworm moth) | Insecta | Lepidoptera | This study | FS | Ne | A | Unknown |
| 2016 | May | 126 | Algonquin, Ontario | Gnaphosa antipola (Stealthy ground spider) | Arachnida | Araneae | This study | FS | Ne | A | Unknown |
| 2016 | May | 126 | Algonquin, Ontario | Phragmatobia fuliginosa (Ruby tiger moth) | Insecta | Lepidoptera | This study | FS | Ne | A | Likely Fresh |
| 2016 | May | 126 | Algonquin, Ontario | Ilybius pleuriticus (Predacious diving-beetle) | Insecta | Coleoptera | This study | FS | Ne | A | Unknown |
| 2016 | May | 126 | Algonquin, Ontario | Hybomitra sp. (Horsefly) | Insecta | Diptera | This study | FS | Ne | A | Unknown |
| 2016 | May | 126 | Algonquin, Ontario | Hemipachnobia monochromatea (Sundew cutworm moth) | Insecta | Lepidoptera | This study | FS | Ne | A | Either Possible |
| 2016 | May | 126 | Algonquin, Ontario | Pseudohermonassa tenuicula (Hair-pin dart moth) | Insecta | Lepidoptera | This study | FS | Ne | A | Either Possible |
| 2016 | May | 126 | Algonquin, Ontario | Dioryctria abietivorella (Fir coneworm moth) | Insecta | Lepidoptera | This study | FS | Ne | A | Either Possible |
| 2016 | May | 126 | Algonquin, Ontario | Cladara limitaria (Mottled gray carpet moth) | Insecta | Lepidoptera | This study | FS | Ne | A | Unknown |
| 2016 | May | 126 | Algonquin, Ontario | Cicurina placida (Meshweaver spider) | Arachnida | Araneae | This study | FS | Ne | A | Unknown |
| 2016 | May | 126 | Algonquin, Ontario | Trochosa terricola (Wolf Spider) | Arachnida | Araneae | This study | FS | Ne | A | Likely Fresh |
| 2016 | May | 126 | Algonquin, Ontario | Ilybius fuliginosus (Predacious diving-beetle) | Insecta | Coleoptera | This study | FS | Ne | A | Unknown |
| 2016 | May | 126 | Algonquin, Ontario | Tipula (Cranefly) | Insecta | Diptera | This study | FS | Ne | A | Unknown |
| 2016 | May | 126 | Algonquin, Ontario | Tipula senega (Cranefly) | Insecta | Diptera | This study | FS | Ne | A | Likely Fresh |
| 2016 | May | 126 | Algonquin, Ontario | Zanclognatha laevigata (Variable zanclognatha moth) | Insecta | Lepidoptera | This study | FS | Ne | A | Either Possible |
| 2016 | May | 126 | Algonquin, Ontario | Gelechiidae | Insecta | Lepidoptera | This study | FS | Ne | A | Unknown |
| 2016 | May | 126 | Algonquin, Ontario | Trypodendron retusum (Poplar ambrosia beetle) | Insecta | Coleoptera | This study | FS | Ne | A | Likely Fresh |
| 2016 | May | 126 | Algonquin, Ontario | Protogygia alberta (Dart moth) | Insecta | Lepidoptera | This study | FS | Ne | A | Unknown |
| 2016 | May | 126 | Algonquin, Ontario | Pachygnatha dorothea (Thick-jawed orbweaver spider) | Arachnida | Araneae | This study | FS | Ne | A | Unknown |
| 2016 | May | 126 | Algonquin, Ontario | Ponometia tortricina (Bird-dropping moth) | Insecta | Lepidoptera | This study | FS | Ne | A | Either Possible |
| 2016 | May | 126 | Algonquin, Ontario | Euphyes bimacula (Two-spotted skipper) | Insecta | Lepidoptera | This study | FS | Ne | A | Either Possible |
| 2016 | May | 126 | Algonquin, Ontario | Podisus serieventris (Predatory stink-bug) | Insecta | Hemiptera | This study | FS | Ne | A | Likely Cached |
| 2016 | May | 126 | Algonquin, Ontario | Alopecosa aculeata (Wolf spider) | Arachnida | Araneae | This study | FS | Ne | A | Either Possible |
| 2016 | May | 126 | Algonquin, Ontario | Mompha solomoni (Momphid moth) | Insecta | Lepidoptera | This study | FS | Ne | A | Error. |
| 2016 | May | 126 | Algonquin, Ontario | Exaeretia canella (Twirler moth) | Insecta | Lepidoptera | This study | FS | Ne | A | Unknown |
| 2016 | May | 126 | Algonquin, Ontario | Agrotis ipsilon (Dark sword-grass moth) | Insecta | Lepidoptera | This study | FS | Ne | A | Either Possible |
| 2016 | May | 126 | Algonquin, Ontario | Protogygia querula (Querula dart moth) | Insecta | Lepidoptera | This study | FS | Ne | A | Unknown |
| 2016 | May | 135 | Algonquin, Ontario | Polydesmidae (Millipede) | Arachnida | Polydesmida | This study | FS | Ne | A | Unknown |
| 2016 | May | 135 | Algonquin, Ontario | Egira dolosa (Lined black aspen moth) | Insecta | Lepidoptera | This study | FS | Ne | A | Likely Fresh |
| 2016 | May | 135 | Algonquin, Ontario | Pterostichus pensylvanicus (Ground beetle) | Insecta | Coleoptera | This study | FS | Ne | A | Likely Fresh |
| 2016 | May | 135 | Algonquin, Ontario | Hemipachnobia monochromatea (Sundew cutworm moth) | Insecta | Lepidoptera | This study | FS | Ne | A | Either Possible |
| 2016 | May | 135 | Algonquin, Ontario | Piratula cantralli (Wolf spider) | Arachnida | Araneae | This study | FS | Ne | A | Unknown |
| 2016 | May | 135 | Algonquin, Ontario | Cybaeopsis tibialis (Hackledmesh weaver spider) | Arachnida | Araneae | This study | FS | Ne | A | Unknown |
| 2016 | May | 135 | Algonquin, Ontario | Cladara limitaria (Mottled gray carpet moth) | Insecta | Lepidoptera | This study | FS | Ne | A | Unknown |
| 2016 | May | 135 | Algonquin, Ontario | Araneus nordmanni (Orbweaver spider) | Arachnida | Araneae | This study | FS | Ne | A | Likely Cached |
| 2016 | May | 135 | Algonquin, Ontario | Pterostichus adstrictus (Ground beetle) | Insecta | Coleoptera | This study | FS | Ne | A | Likely Fresh |
| 2016 | May | 135 | Algonquin, Ontario | Cicurina arcuata (Meshweaver spider) | Arachnida | Araneae | This study | FS | Ne | A | Likely Fresh |
| 2016 | May | 135 | Algonquin, Ontario | Orthocladiinae (Midge) | Insecta | Diptera | This study | FS | Ne | A | Likely Fresh |
| 2016 | May | 135 | Algonquin, Ontario | Criorhina nigriventris (Hover fly) | Insecta | Diptera | This study | FS | Ne | A | Likely Fresh |
| 2016 | May | 135 | Algonquin, Ontario | Podisus serieventris (Predatory stink-bug) | Insecta | Hemiptera | This study | FS | Ne | A | Either Possible |
| 2016 | May | 135 | Algonquin, Ontario | Polydesmidae (Millipede) | Arachnida | Polydesmida | This study | FS | Ne | A | Unknown |
| 2016 | May | 135 | Algonquin, Ontario | Callobius bennetti (Hackledmesh weaver spider) | Arachnida | Araneae | This study | FS | Ne | A | Either Possible |
| 2016 | May | 135 | Algonquin, Ontario | Egira dolosa (Lined black aspen moth) | Insecta | Lepidoptera | This study | FS | Ne | A | Likely Fresh |
| 2016 | May | 135 | Algonquin, Ontario | Hemipachnobia monochromatea (Sundew cutworm moth) | Insecta | Lepidoptera | This study | FS | Ne | A | Either Possible |
| 2016 | May | 135 | Algonquin, Ontario | Pterostichus pensylvanicus (Ground beetle) | Insecta | Coleoptera | This study | FS | Ne | A | Likely Fresh |
| 2016 | May | 135 | Algonquin, Ontario | Pterostichus pensylvanicus (Ground beetle) | Insecta | Coleoptera | This study | FS | Ne | A | Likely Fresh |
| 2016 | May | 135 | Algonquin, Ontario | Pterostichus adstrictus (Ground beetle) | Insecta | Coleoptera | This study | FS | Ne | A | Likely Fresh |
| 2016 | May | 135 | Algonquin, Ontario | Araneus nordmanni (Orbweaver spider) | Arachnida | Araneae | This study | FS | Ne | A | Likely Cached |
| 2016 | May | 135 | Algonquin, Ontario | Callobius bennetti (Hackledmesh weaver spider) | Arachnida | Araneae | This study | FS | Ne | A | Either Possible |
| 2017 | April | 116 | Algonquin, Ontario | Vaccinium angustifolium (Early low-bush blueberry) | Magnoliopsida | Ericales | This study | FS | Ne | P | Likely Cached |
| 2017 | April | 116 | Algonquin, Ontario | Pinus (Pine) | Pinopsida | Pinales | This study | FS | Ne | P | Either possible |
| 2017 | April | 120 | Algonquin, Ontario | Elymus trachycaulus (Slender wildrye) | Liliopsida | Poales | This study | FS | Ne | P | Either Possible |
| 2017 | April | 120 | Algonquin, Ontario | Triticum aestivum (Common wheat) | Liliopsida | Poales | This study | FS | Ne | P | Either Possible |
| 2017 | April | 120 | Algonquin, Ontario | Vaccinium angustifolium (Early low-bush blueberry) | Magnoliopsida | Ericales | This study | FS | Ne | P | Likely Cached |
| 2017 | April | 120 | Algonquin, Ontario | Gaultheria hispidula (Creeping Snowberry) | Magnoliopsida | Ericales | This study | FS | Ne | P | Likely Cached |
| 2017 | April | 120 | Algonquin, Ontario | Pinus (Pine) | Pinopsida | Pinales | This study | FS | Ne | P | Either possible |
| 2017 | April | 120 | Algonquin, Ontario | Populus tremuloides (Trembling Aspen) | Magnoliopsida | Malpighiales | This study | FS | Ne | P | Likely Fresh |
| 2017 | April | 120 | Algonquin, Ontario | Betula papyrifera (White birch) | Magnoliopsida | Fagales | This study | FS | Ne | P | Either possible |
| 2017 | April | 120 | Algonquin, Ontario | Chamaedaphne calyculata (Leatherleaf) | Magnoliopsida | Ericales | This study | FS | Ne | P | Either possible |
| 2017 | April | 120 | Algonquin, Ontario | Epigaea repens (Trailing Arbutus) | Magnoliopsida | Ericales | This study | FS | Ne | P | Likely Cached |
| 2017 | April | 120 | Algonquin, Ontario | Pyrola asarifolia (Pink pyrola) | Magnoliopsida | Ericales | This study | FS | Ne | P | Likely Cached |
| 2017 | April | 120 | Algonquin, Ontario | Taxus canadensis (Canada yew) | Pinopsida | Pinales | This study | FS | Ne | P | Likely Cached |
| 2017 | April | 120 | Algonquin, Ontario | Elymus trachycaulus (Slender wildrye) | Liliopsida | Poales | This study | FS | Ne | P | Either Possible |
| 2017 | April | 120 | Algonquin, Ontario | Salix discolor (Pussy willow) | Magnoliopsida | Malpighiales | This study | FS | Ne | P | Likely Fresh |
| 2017 | April | 120 | Algonquin, Ontario | Vaccinium angustifolium (Early low-bush blueberry) | Magnoliopsida | Ericales | This study | FS | Ne | P | Likely Cached |
| 2017 | April | 120 | Algonquin, Ontario | Pyrola asarifolia (Pink pyrola) | Magnoliopsida | Ericales | This study | FS | Ne | P | Likely Cached |
| 2017 | April | 120 | Algonquin, Ontario | Epigaea repens (Trailing Arbutus) | Magnoliopsida | Ericales | This study | FS | Ne | P | Likely Cached |
| 2017 | April | 120 | Algonquin, Ontario | Vaccinium angustifolium (Early low-bush blueberry) | Magnoliopsida | Ericales | This study | FS | Ne | P | Likely Cached |
| 2017 | April | 120 | Algonquin, Ontario | Vaccinium angustifolium (Early low-bush blueberry) | Magnoliopsida | Ericales | This study | FS | Ne | P | Likely Cached |
| 2017 | April | 120 | Algonquin, Ontario | Pinus (Pine) | Pinopsida | Pinales | This study | FS | Ne | P | Either possible |
| 2016 | May | 126 | Algonquin, Ontario | Vaccinium angustifolium (Early low-bush blueberry) | Magnoliopsida | Ericales | This study | FS | Ne | P | Likely Cached |
| 2016 | May | 126 | Algonquin, Ontario | Betula papyrifera (White birch) | Magnoliopsida | Fagales | This study | FS | Ne | P | Either possible |
| 2016 | May | 126 | Algonquin, Ontario | Agrostis scabra (Rough Bentgrass) | Liliopsida | Poales | This study | FS | Ne | P | Likely Cached |
| 2016 | May | 126 | Algonquin, Ontario | Cornus canadensis (Bunchberry) | Magnoliopsida | Cornales | This study | FS | Ne | P | Likely Cached |
| 2016 | May | 126 | Algonquin, Ontario | Pyrola asarifolia (Pink pyrola) | Magnoliopsida | Ericales | This study | FS | Ne | P | Likely Cached |
| 2016 | May | 126 | Algonquin, Ontario | Taxus canadensis (Canada yew) | Pinopsida | Pinales | This study | FS | Ne | P | Likely Cached |
| 2016 | May | 126 | Algonquin, Ontario | Cornus sericea (Red-osier dogwood | Magnoliopsida | Cornales | This study | FS | Ne | P | Either possible |
| 2016 | May | 135 | Algonquin, Ontario | Vaccinium angustifolium (Early low-bush blueberry) | Magnoliopsida | Ericales | This study | FS | Ne | P | Likely Cached |
| 2016 | May | 135 | Algonquin, Ontario | Vaccinium angustifolium (Early low-bush blueberry) | Magnoliopsida | Ericales | This study | FS | Ne | P | Likely Cached |
| 2016 | May | 135 | Algonquin, Ontario | Pinus (Pine) | Pinopsida | Pinales | This study | FS | Ne | P | Either possible |
| 2016 | May | 135 | Algonquin, Ontario | Chamaedaphne calyculata (Leatherleaf) | Magnoliopsida | Ericales | This study | FS | Ne | P | Either possible |
| 2016 | May | 135 | Algonquin, Ontario | Vaccinium angustifolium (Early low-bush blueberry) | Magnoliopsida | Ericales | This study | FS | Ne | P | Likely Cached |
| 2016 | May | 135 | Algonquin, Ontario | Gaultheria hispidula (Creeping Snowberry) | Magnoliopsida | Ericales | This study | FS | Ne | P | Likely Cached |
| 2016 | May | 135 | Algonquin, Ontario | Pinus (Pine) | Pinopsida | Pinales | This study | FS | Ne | P | Either possible |
| 2017 | April | 120 | Algonquin, Ontario | Sorex cinereus (Cinereous Shrew) | Mammalia | Soricomorpha | This study | FS | Ne | V | Either Possible |
| 2016 | May | 135 | Algonquin, Ontario | Rana sylvatica (Wood frog)) | Amphibia | Anura | This study | FS | Ne | V | Either Possible |
| 2016 | May | 135 | Algonquin, Ontario | Sorex cinereus (Cinereous Shrew) | Mammalia | Soricomorpha | This study | FS | Ne | V | Either Possible |
| 1982 | January | 3 | Gogama, Ontario | Unidentifiable insect parts | Insecta | Unknown | DS, unpublished | SC | Ad | A | Unknown |
| 1982 | January | 3 | Gogama, Ontario | Diplopoda - 1 Julidae | Diplopoda | Julida | DS, unpublished | SC | Ad | A | Unknown |
| 1982 | January | 3 | Gogama, Ontario | Phalangida - 4 | Arachnida | Phalangida | DS, unpublished | SC | Ad | A | Unknown |
| 1982 | January | 3 | Gogama, Ontario | Several Coleoptera | Insecta | Coleoptera | DS, unpublished | SC | Ad | A | Unknown |
| 1982 | January | 3 | Gogama, Ontario | Coleoptera - 1 Curculionidae | Insecta | Coleoptera | DS, unpublished | SC | Ad | A | Unknown |
| 1982 | January | 3 | Gogama, Ontario | Orthoptera - Acrididae? | Insecta | Orthoptera | DS, unpublished | SC | Ad | A | Unknown |
| 1982 | January | 3 | Gogama, Ontario | Hymenoptera - 1 Vespula sp | Insecta | Hymenoptera | DS, unpublished | SC | Ad | A | Unknown |
| 1971 | January | 9 | Algonquin, Ontario | "various insect remains, larval beetle (?) parts" | Insecta | Coleoptera (?) | F.W. Schueler, unpublished ms | SC | Ad | A | Unknown |
| 1971 | January | 9 | Algonquin, Ontario | "Much varied insect material, Buprestid parts (elytra, etc.)" | Insecta | Coleoptera | F.W. Schueler, unpublished ms | SC | Ad | A | Unknown |
| 1971 | January |  | Algonquin, Ontario | "Heads of a large cicadellid" | Insecta | Hemiptera | F.W. Schueler, unpublished ms | SC | Ad | A | Unknown |
| 1971 | January |  | Algonquin, Ontario | Heads of Camponotus | Insecta | Hymenoptera | F.W. Schueler, unpublished ms | SC | Ad | A | Unknown |
| 1982 | January | 10 | Gogama, Ontario | Many insect fragments | Insecta | Unknown | DS, unpublished | SC | Ad | A | Unknown |
| 1982 | January | 10 | Gogama, Ontario | Coleoptera | Insecta | Coleoptera | DS, unpublished | SC | Ad | A | Unknown |
| 1982 | January | 10 | Gogama, Ontario | Hemiptera | Insecta | Hemiptera | DS, unpublished | SC | Ad | A | Unknown |
| 1982 | January | 17 | Gogama, Ontario | Hymenoptera - 1 Vespula sp | Insecta | Hymenoptera | DS, unpublished | SC | Ad | A | Unknown |
| 1982 | January | 19 | Gogama, Ontario | Insect fragments - Coleoptera | Insecta | Coleoptera | DS, unpublished | SC | Ad | A | Unknown |
| 1982 | January | 31 | Gogama, Ontario | Orthoptera - several Melanoplus sp | Insecta | Orthoptera | DS, unpublished | SC | Ad | A | Unknown |
| 1983 | February | 44 | Gogama, Ontario | Lepidoptera - 1 pupa | Insecta | Lepidoptera | DS, unpublished | SC | Ad | A | Unknown |
| 1983 | February | 44 | Gogama, Ontario | Lepidoptera - egg mass of Malacosoma dysstria | Insecta | Lepidoptera | DS, unpublished | SC | Ad | A | Unknown |
| 1983 | February | 44 | Gogama, Ontario | Coleoptera - 1 | Insecta | Coleoptera | DS, unpublished | SC | Ad | A | Unknown |
| 1983 | February | 44 | Gogama, Ontario | Hymenoptera - 1 | Insecta | Hymenoptera | DS, unpublished | SC | Ad | A | Unknown |
| 1983 | February | 46 | Gogama, Ontario | Coleoptera - 1 or 2 | Insecta | Coleoptera | DS, unpublished | SC | Ad | A | Unknown |
| 1983 | February | 46 | Gogama, Ontario | Hymenoptera - 1 or 2 | Insecta | Hymenoptera | DS, unpublished | SC | Ad | A | Unknown |
| 1983 | February | 46 | Gogama, Ontario | Hemiptera - 1 Pentatomidae | Insecta | Hemiptera | DS, unpublished | SC | Ad | A | Unknown |
| 1983 | February | 46 | Gogama, Ontario | Lepidoptera - 1 Noctuid larva | Insecta | Lepidoptera | DS, unpublished | SC | Ad | A | Unknown |
| 1983 | February | 46 | Gogama, Ontario | Lepidoptera - 1 Tenthredinid cocoon (Diprion?) | Insecta | Lepidoptera | DS, unpublished | SC | Ad | A | Unknown |
| 1983 | March | 61 | Gogama, Ontario | A few insect fragments | Insecta | Unknown | DS, unpublished | SC | Ad | A | Unknown |
| 1983 | March | 61 | Gogama, Ontario | Hemiptera - 1 Pentatomidae | Insecta | Hemiptera | DS, unpublished | SC | Ad | A | Unknown |
| 1951 | May | 139 | Canmore, Alberta | ? | Insecta | ? | ROM79162 | SC | Ad | A |  |
| 1955 | May | 142 | Moyie, British Columbia | "Beetles" | Insecta | Coleoptera | ROM73764 | SC | Ad | A |  |
| 1957 | June | 152 | Spruce Grove, Alberta | ? | Insecta | ? | ROM77005 | SC | Ad | A |  |
| 1970 | June | 158 | Algonquin, Ontario | Coleoptera - 1 Agriotes stabilis (LeC.) | Insecta | Coleoptera | DS, unpublished | SC | Ad | A |  |
| 1970 | June | 158 | Algonquin, Ontario | Coleoptera - 1 probable Agriotes quebecensis Brown | Insecta | Coleoptera | DS, unpublished | SC | Ad | A |  |
| 1970 | June | 158 | Algonquin, Ontario | Coleoptera - several Agriotes sp | Insecta | Coleoptera | DS, unpublished | SC | Ad | A |  |
| 1970 | June | 158 | Algonquin, Ontario | Coleoptera - 1 Carabidae | Insecta | Coleoptera | DS, unpublished | SC | Ad | A |  |
| 1970 | June | 158 | Algonquin, Ontario | Coleoptera - 1 possible Curculionidae | Insecta | Coleoptera | DS, unpublished | SC | Ad | A |  |
| 1970 | June | 158 | Algonquin, Ontario | Hemiptera - several Pentatomidae (Eustichus?) | Insecta | Hemiptera | DS, unpublished | SC | Ad | A |  |
| 1970 | June | 158 | Algonquin, Ontario | Hymenoptera - 1 carpenter ant | Insecta | Hymenoptera | DS, unpublished | SC | Ad | A |  |
| 1970 | June | 158 | Algonquin, Ontario | Spiders too broken for identification | Arachnida | Araneae | DS, unpublished | SC | Ad | A |  |
| 1956 | June | 160 | Eagle Lake, Alberta | ? | Insecta | ? | ROM76531 | SC | Ad | A |  |
| Not given | June | 164 | Labrador | Small beetle - 1 | Insecta | Coleoptera | Clement in Todd 1963 | SC | Ad | A |  |
| 2021 | June | 168 | Vancouver Island, BC | Unidentified arthrpod exoskeletal fragments | Insecta + others? | Coleoptera + others? | DS, unpublished (photo) | SC | Ad | A |  |
| 1833 | June | 178 | Labrador | "Insects, […], eggs of ants" | Insecta | Unknown | Audubon 1841 | SC | Ad | A |  |
| 1979 | July | 193 | Algonquin, Ontario | Lepidoptera - pupa resembling spruce budworm which would be at observed stage in June | Insecta | Lepidoptera | DS, unpublished | SC | Ad | A |  |
| 1980 | July | 196 | Algonquin, Ontario | Coleoptera fragments | Insecta | Coleoptera | DS, unpublished | SC | Ad | A |  |
| 1980 | July | 196 | Algonquin, Ontario | Hemiptera fragments | Insecta | Hemiptera | DS, unpublished | SC | Ad | A |  |
| 1980 | July | 196 | Algonquin, Ontario | Diptera - Tabanidae - 6 Hybomitra sp larvae | Insecta | Diptera | DS, unpublished | SC | Ad | A |  |
| 1980 | July | 196 | Algonquin, Ontario | Diptera - 6 probable Syrphus sp larvae (Syrphidae - Syrphinae) | Insecta | Diptera | DS, unpublished | SC | Ad | A |  |
| 1950 | July | 203 | Mt. Coakley, British Columbia | "Hard shelled insects, a large brown weevil and several green caterpillars | Insecta |  | ROM77764 | SC | Ad | A |  |
| 1927 | August | 219 | Mt. Arrowhead, BC | "and beetles" | Insecta | Coleoptera | ROM1191 | SC | Ad | A |  |
| 1954 | September | 255 | James River, Alberta | "Insects" | Insecta | ? | ROM72052 | SC | Ad | A |  |
| 1984 | September | 258 | Algonquin, Ontario | Diptera - Syrphidae - 1 Eristalis dimidiata Wiedeman | Insecta | Diptera | DS, unpublished | SC | Ad | A |  |
| 1984 | September | 258 | Algonquin, Ontario | Spiders - possibly Xysitcus sp | Arachnida | Araneae | DS, unpublished | SC | Ad | A |  |
| 1984 | September | 258 | Algonquin, Ontario | Spiders - possibly Clubiona sp | Arachnida | Araneae | DS, unpublished | SC | Ad | A |  |
| 1984 | September | 258 | Algonquin, Ontario | Insect fragments | Insecta | Unknown | DS, unpublished | SC | Ad | A |  |
| 1950 | September | 259 | Canmore, Alberta | "grasshopper" | Insecta | Orthoptera | ROM78616 | SC | Ad | A |  |
| 1939 | September | 263 | Cariboo, British Columbia | Adults and pupae of a large dipterous insect, frgments of small beetles and other insects | Insecta | Unknown | Munro 1945 | SC | Ad | A |  |
| 1904 | September | 271 | North Bay, Ontario | "grasshoppers" | Insecta | Orthoptera | ROM92717 | SC | Ad | A |  |
| 1905 | September | 271 | Ottawa river, Québec | Wasps from a wasp nest frequently found in Gray jay stomachs according to a "Mr. Kingston" cited by author | Insecta | Hymenoptera | Eifrig 1906 "Notes on Northern Birds Auk 23:316-317 | SC | Ad | A |  |
| Not given | Fall? | 311 | Not given | "Numerous remains of insects, a large hairy caterpillar" | Insecta | Unknown | Audubon 1841 | SC | Ad | A |  |
| Not given | Fall? | 311 | Not given | "Numerous remains of insects, a large hairy caterpillar" | Insecta | Lepidoptera | Audubon 1841 | SC | Ad | A |  |
| 1950 | October | 279 | Canmore, Alberta | "Insects" | Insecta |  | ROM78617 | SC | Ad | A |  |
| 1950 | October | 279 | Canmore, Alberta | "Insects, including grasshoppers" | Insecta | Orthoptera | ROM78618 | SC | Ad | A |  |
| 1973 | October | 282 | Algonquin, Ontario | Hymenoptera - 2 Ichneumoninae sp | Insecta | Hymenoptera | DS, unpublished | SC | Ad | A |  |
| 1973 | October | 282 | Algonquin, Ontario | Hymenoptera - Lasius neoniger Emery (50 queens, 3 males, 6 workers) | Insecta | Hymenoptera | DS, unpublished | SC | Ad | A |  |
| 1980 | October | 283 | Algonquin, Ontario | Spider - Araneae - 1 unidentified | Arachnida | Araneae | DS, unpublished | SC | Ad | A |  |
| 1980 | October | 283 | Algonquin, Ontario | Lepidoptera - 1 Geometrid larva | Insecta | Lepidoptera | DS, unpublished | SC | Ad | A |  |
| 1980 | October | 283 | Algonquin, Ontario | Lepidoptera - 1 unidentified larva | Insecta | Lepidoptera | DS, unpublished | SC | Ad | A |  |
| 1980 | October | 283 | Algonquin, Ontario | Coleoptera - 3 unidentified | Insecta | Coleoptera | DS, unpublished | SC | Ad | A |  |
| 1980 | October | 283 | Algonquin, Ontario | Coleoptera - Cantharidae - 2 Catharis sp | Insecta | Coleoptera | DS, unpublished | SC | Ad | A |  |
| 1980 | October | 283 | Algonquin, Ontario | Hemiptera - 2 Pentatomidae | Insecta | Hemiptera | DS, unpublished | SC | Ad | A |  |
| 1978 | October | 284 | Algonquin, Ontario | Hymenoptera - 4 Formicidae | Insecta | Hymenoptera | DS, unpublished | SC | Ad | A |  |
| 1978 | October | 284 | Algonquin, Ontario | Orthoptera - 1 Acrididae | Insecta | Orthoptera | DS, unpublished | SC | Ad | A |  |
| 1978 | October | 284 | Algonquin, Ontario | Orthoptera - 2 Gryllidae | Insecta | Orthoptera | DS, unpublished | SC | Ad | A |  |
| 1978 | October | 284 | Algonquin, Ontario | Coleoptera - 1 Curculionidae | Insecta | Coleoptera | DS, unpublished | SC | Ad | A |  |
| 1978 | October | 284 | Algonquin, Ontario | Hemiptera - 1 Membracidae | Insecta | Hemiptera | DS, unpublished | SC | Ad | A |  |
| 1972 | October | 285 | Algonquin, Ontario | Unidentifiable insect parts | Insecta | Unknown | DS, unpublished | SC | Ad | A |  |
| 1972 | October | 285 | Bracebridge, Ontario | Coleoptera - 1 Sphaeroderus lecontei Dej. | Insecta | Coleoptera | DS, unpublished | SC | Ad | A |  |
| 1972 | October | 285 | Bracebridge, Ontario | Coleoptera - 10 Pterostichus adstrictus Esch. | Insecta | Coleoptera | DS, unpublished | SC | Ad | A |  |
| 1972 | October | 285 | Bracebridge, Ontario | Lepidoptera - 1 Geometridae, probably Ennominae | Insecta | Lepidoptera | DS, unpublished | SC | Ad | A |  |
| 1972 | October | 285 | Bracebridge, Ontario | Diplopoda - parts of one centipede | Diplopoda | Unknown | DS, unpublished | SC | Ad | A |  |
| 1977 | October | 288 | La Vérendrye, Québec | Hymenoptera - Ichneunomidae - 1 Ichneumon sp | Insecta | Hymenoptera | DS, unpublished | SC | Ad | A |  |
| 1977 | October | 288 | La Vérendrye, Québec | Hymenoptera - Ichneunomidae - 1 Ichneumonae sp | Insecta | Hymenoptera | DS, unpublished | SC | Ad | A |  |
| 1981 | October | 288 | Ignace, Ontario | Insect fragments - Coleoptera? | Insecta | Coleoptera | DS, unpublished | SC | Ad | A |  |
| 1981 | October | 288 | Ignace, Ontario | Hymenoptera - 2+ Vespula? | Insecta | Hymenoptera | DS, unpublished | SC | Ad | A |  |
| 1979 | October | 289 | Algonquin, Ontario | Orthoptera - 2 melanoplus sanguinipes F. | Insecta | Orthoptera | DS, unpublished | SC | Ad | A |  |
| 1979 | October | 289 | Algonquin, Ontario | Hymenoptera - 1 Vespula vulgaris L. | Insecta | Hymenoptera | DS, unpublished | SC | Ad | A |  |
| 1979 | October | 289 | Algonquin, Ontario | Lepidoptera - 1 larva | Insecta | Lepidoptera | DS, unpublished | SC | Ad | A |  |
| 1979 | October | 289 | Algonquin, Ontario | Coleoptera - 1 Chrysomelidae? | Insecta | Coleoptera | DS, unpublished | SC | Ad | A |  |
| 1901 | October | 290 | Port Sydney, Ontario | "Insects" | Insecta | Unknown | ROM69578 | SC | Ad | A |  |
| 1979 | October | 292 | Algonquin, Ontario | Hemiptera - 2 or 3 Eustichus sp | Insecta | Hemiptera | DS, unpublished | SC | Ad | A |  |
| 1979 | October | 292 | Algonquin, Ontario | Coleoptera - 2 or 3 Curculionidae | Insecta | Coleoptera | DS, unpublished | SC | Ad | A |  |
| 1979 | October | 292 | Algonquin, Ontario | Coleoptera - 3 larvae | Insecta | Coleoptera | DS, unpublished | SC | Ad | A |  |
| 1979 | October | 292 | Algonquin, Ontario | Neuroptera - 1 Chrysopidae larva | Insecta | Neuroptera | DS, unpublished | SC | Ad | A |  |
| 1979 | October | 292 | Algonquin, Ontario | Orthoptera - 2 or 3 Gryllidae | Insecta | Orthoptera | DS, unpublished | SC | Ad | A |  |
| 1979 | October | 292 | Algonquin, Ontario | Isopoda - probably Oniscus sp. | Malacostraca | Isopoda | DS, unpublished | SC | Ad | A |  |
| 1912 | October | 294 | Wabigoon, Ontario | "Insects etc." | Insecta | Unknown | ROM69578 | SC | Ad | A |  |
| 1898 | October | 297 | Port Sydney, Ontario | "Grasshoppers and small beetles" | Insecta | Orthoptera & Coleoptera | ROM261011207 | SC | Ad | A |  |
| 1981 | October | 299 | Ignace, Ontario | Orthoptera - Several Acrididae | Insecta | Orthoptera | DS, unpublished | SC | Ad | A |  |
| 1972 | October | 300 | Algonquin, Ontario | Coleoptera - Carabidae - 1 Agonumspp | Insecta | Coleoptera | DS, unpublished | SC | Ad | A |  |
| 1972 | October | 300 | Algonquin, Ontario | Coleoptera - Carabidae - 1 Pterostichus sp | Insecta | Coleoptera | DS, unpublished | SC | Ad | A |  |
| 1972 | October | 300 | Algonquin, Ontario | Hymenoptera | Insecta | Hymenoptera | DS, unpublished | SC | Ad | A |  |
| 1972 | October | 300 | Algonquin, Ontario | Orthoptera - 2 Philonthus spp | Insecta | Orthoptera | DS, unpublished | SC | Ad | A |  |
| 1972 | October | 300 | Algonquin, Ontario | Orthoptera - Gryllidae - probably 1 Nemobius sp | Insecta | Orthoptera | DS, unpublished | SC | Ad | A |  |
| 1896 | October | 301 | Port Sydney, Ontario | "Insects" | Insecta | Unknown | ROM1211231169 | SC | Ad | A |  |
| 1983 | October | 302 | Algonquin, Ontario | Lepidoptera - 2 larvae | Insecta | Lepidoptera | DS, unpublished | SC | Ad | A |  |
| 1983 | October | 302 | Algonquin, Ontario | Coleoptera - Carabidae - 1 Cymindis cribricollis | Insecta | Coleoptera | DS, unpublished | SC | Ad | A |  |
| 1983 | October | 302 | Algonquin, Ontario | Possibly Spiders - Xysticus sp | Arachnida | Araneae | DS, unpublished | SC | Ad | A |  |
| 1983 | October | 302 | Algonquin, Ontario | Possibly Spiders - Clubiona sp | Arachnida | Araneae | DS, unpublished | SC | Ad | A |  |
| 1983 | October | 302 | Algonquin, Ontario | Possibly Hymenoptera - Ichneunomidae - Ichneunominae | Insecta | Hymenoptera | DS, unpublished | SC | Ad | A |  |
| 1983 | October | 302 | Algonquin, Ontario | Possibly other insects | Insecta | Unknown | DS, unpublished | SC | Ad | A |  |
| 1898 | October | 302 | Port Sydney, Ontario | "Grasshoppers & insects" | Insecta | Unknown | ROM261011199 | SC | Ad | A |  |
| 1977 | October | 303 | Algonquin, Ontario | Coleoptera - 1 Otiorhynchus sp | Insecta | Coleoptera | DS, unpublished | SC | Ad | A |  |
| 1977 | October | 303 | Algonquin, Ontario | Orthoptera - probably Sulcatus Fab. | Insecta | Orthoptera | DS, unpublished | SC | Ad | A |  |
| 1977 | October | 303 | Algonquin, Ontario | Orthoptera - Gryllidae - probably 1 Gryllus veletis (Alexander and Bigelow) | Insecta | Orthoptera | DS, unpublished | SC | Ad | A |  |
| 1977 | October | 303 | Algonquin, Ontario | Heteroptera - 1 Pentatomidae sp | Insecta | Hemiptera | DS, unpublished | SC | Ad | A |  |
| 1977 | October | 303 | Algonquin, Ontario | Heteroptera - 5 Lygaceidae sp | Insecta | Hemiptera | DS, unpublished | SC | Ad | A |  |
| 1977 | October | 303 | Algonquin, Ontario | Spider - Aranaea - 1 Amaurobiidae (juvenile) | Arachnida | Araneae | DS, unpublished | SC | Ad | A |  |
| Not given | Fall? | 311 | Maine | Carrion beetles | Insecta | Coleoptera | Hardy in Bendire 1895 | SC | Ad | A |  |
| 1981 | November | 310 | Ignace, Ontario | Orthoptera - 2 or 3 Acrididae | Insecta | Orthoptera | DS, unpublished | SC | Ad | A | Unknown |
| 1979 | November | 310 | Algonquin, Ontario | Coleoptera insect fragments | Insecta | Coleoptera | DS, unpublished | SC | Ad | A | Unknown |
| 1979 | November | 310 | Algonquin, Ontario | Hymenoptera insect fragments | Insecta | Hymenoptera | DS, unpublished | SC | Ad | A | Unknown |
| 1979 | November | 310 | Algonquin, Ontario | Hemiptera insect fragments | Insecta | Hemiptera | DS, unpublished | SC | Ad | A | Unknown |
| 1979 | November | 310 | Algonquin, Ontario | Lepidoptera - several Noctuidae - 2 spp | Insecta | Lepidoptera | DS, unpublished | SC | Ad | A | Unknown |
| 1984 | November | 311 | Algonquin, Ontario | Lepidoptera - Noctuidae skin and fragments (1) | Insecta | Lepidoptera | DS, unpublished | SC | Ad | A | Unknown |
| 1984 | November | 311 | Algonquin, Ontario | Coleoptera - Carabidae - 1 Pterostichus patruelis | Insecta | Coleoptera | DS, unpublished | SC | Ad | A | Likely Cached |
| 1984 | November | 311 | Algonquin, Ontario | Coleoptera - Dermestidae - 1 Cryptorhopalium haemorrhoidale | Insecta | Coleoptera | DS, unpublished | SC | Ad | A | Unknown |
| 1984 | November | 311 | Algonquin, Ontario | Possibly spiders | Arachnida | Araneae | DS, unpublished | SC | Ad | A | Unknown |
| 1984 | November | 311 | Algonquin, Ontario | Possibly other insects | Insecta | Unknown | DS, unpublished | SC | Ad | A | Unknown |
| 1981 | November | 314 | Ignace, Ontario | Orthoptera - 1 Melanoplus sanguinipes (Fabricius) | Insecta | Orthoptera | DS, unpublished | SC | Ad | A | Likely Cached |
| 1981 | November | 314 | Ignace, Ontario | Orthoptera - 2 Melanoplus | Insecta | Orthoptera | DS, unpublished | SC | Ad | A | Unknown |
| 1981 | November | 314 | Ignace, Ontario | Coleoptera - parts of several species | Insecta | Coleoptera | DS, unpublished | SC | Ad | A | Unknown |
| 1972 | November | 315 | Algonquin, Ontario | "Insects" | Insecta | Unknown | R. J. Rutter, pers. comm. | SC | Ad | A | Unknown |
| 1981 | November | 318 | Algonquin, Ontario | Coleoptera - 1 Carabidae larva | Insecta | Coleoptera | DS, unpublished | SC | Ad | A | Unknown |
| 1981 | November | 318 | Algonquin, Ontario | Coleoptera - several | Insecta | Coleoptera | DS, unpublished | SC | Ad | A | Unknown |
| 1981 | November | 318 | Algonquin, Ontario | Hemiptera - several Pentatomidae | Insecta | Hemiptera | DS, unpublished | SC | Ad | A | Unknown |
| 1981 | November | 318 | Algonquin, Ontario | Orthoptera - 1 Gryllidae | Insecta | Orthoptera | DS, unpublished | SC | Ad | A | Unknown |
| 1981 | November | 318 | Algonquin, Ontario | Orthoptera - 1 Melanoplus sp | Insecta | Orthoptera | DS, unpublished | SC | Ad | A | Unknown |
| Not given | November | 319 | Wisconsin | Phyncophora - 1 large Weevil (Curculionidae) | Insecta | Coleoptera | Bennetts 1900 | SC | Ad | A | Unknown |
| Not given | November | 319 | Wisconsin | Hymenoptera - 1 | Insecta | Hymenoptera | Bennetts 1900 | SC | Ad | A | Unknown |
| Not given | November | 319 | Wisconsin | Hemiptera - 1 Capsid | Insecta | Hemiptera | Bennetts 1900 | SC | Ad | A | Unknown |
| Not given | November | 319 | Wisconsin | Tettix grasshoper - 1 | Insecta | Orthoptera | Bennetts 1900 | SC | Ad | A | Unknown |
| Not given | November | 319 | Wisconsin | Several caterpillars | Insecta | Lepidoptera | Bennetts 1900 | SC | Ad | A | Unknown |
| 1981 | November | 319 | Temagami (Cassels twp), Ontario | Hymenoptera - several Ichneunomidae - 4 spp | Insecta | Hymenoptera | DS, unpublished | SC | Ad | A | Unknown |
| 1981 | November | 319 | Temagami (Cassels twp), Ontario | Hemiptera - several Pentatomidae? | Insecta | Hemiptera | DS, unpublished | SC | Ad | A | Unknown |
| 1981 | November | 319 | Temagami (Cassels twp), Ontario | Coleoptera - several (3-4 spp) | Insecta | Coleoptera | DS, unpublished | SC | Ad | A | Unknown |
| 1981 | November | 319 | Temagami (Cassels twp), Ontario | Insect fragments | Insecta | Unknown | DS, unpublished | SC | Ad | A | Unknown |
| 1981 | November | 319 | Temagami (Cassels twp), Ontario | Diplopoda - 1 Julidae | Diplopoda | Julida | DS, unpublished | SC | Ad | A | Unknown |
| 1981 | November | 319 | Temagami (Cassels twp), Ontario | Hymenoptera - several Ichneunomidae - at least 3 spp | Insecta | Hymenoptera | DS, unpublished | SC | Ad | A | Unknown |
| 1981 | November | 319 | Temagami (Cassels twp), Ontario | Hemiptera - several Pentatomidae | Insecta | Hemiptera | DS, unpublished | SC | Ad | A | Unknown |
| Not given | November | 319 | New York | Insects | Insecta | Unknown | Wilson and Bonaparte 1831 | SC | Ad | A | Unknown |
| 1951 | November | 321 | White River, Ontario | "Insects" | Insecta | Unknown | ROM10779 | SC | Ad | A | Unknown |
| 1981 | November | 322 | Ignace, Ontario | Coleoptera - 1 Curculionidae | Insecta | Coleoptera | DS, unpublished | SC | Ad | A | Unknown |
| 1981 | November | 322 | Ignace, Ontario | Coleoptera - several | Insecta | Coleoptera | DS, unpublished | SC | Ad | A | Unknown |
| 1981 | November | 322 | Ignace, Ontario | Hemiptera - Pentatomidae | Insecta | Hemiptera | DS, unpublished | SC | Ad | A | Unknown |
| 1981 | November | 322 | Ignace, Ontario | Orthoptera | Insecta | Orthoptera | DS, unpublished | SC | Ad | A | Unknown |
| 1981 | November | 322 | Ignace, Ontario | Spider - Araneae - 1 or 2 | Arachnida | Araneae | DS, unpublished | SC | Ad | A | Unknown |
| 1982 | November | 326 | Chapleau, Ontario | Hemiptera - 1 Pentatomidae | Insecta | Hemiptera | DS, unpublished | SC | Ad | A | Unknown |
| 1982 | November | 326 | Chapleau, Ontario | Hymenoptera - 1 Ichneunomidae (pupa) | Insecta | Hymenoptera | DS, unpublished | SC | Ad | A | Unknown |
| 1982 | November | 326 | Chapleau, Ontario | Hymenoptera - 1 Formicidae | Insecta | Hymenoptera | DS, unpublished | SC | Ad | A | Unknown |
| 1982 | November | 326 | Chapleau, Ontario | Lepidoptera - 1 pupa | Insecta | Lepidoptera | DS, unpublished | SC | Ad | A | Unknown |
| 1982 | November | 326 | Chapleau, Ontario | Hemiptera - several Pentatomidae | Insecta | Hemiptera | DS, unpublished | SC | Ad | A | Unknown |
| 1982 | November | 326 | Chapleau, Ontario | Hymenoptera - several Formicidae | Insecta | Hymenoptera | DS, unpublished | SC | Ad | A | Unknown |
| Not given | Late November | 326 | New York | A few spiders | Arachnida | Araneae | Wilson and Bonaparte 1830 | SC | Ad | A | Unknown |
| Not given | Late November | 326 | New York | A few insects | Insecta | Unknown | Wilson and Bonaparte 1830 | SC | Ad | A | Unknown |
| 1980 | November | 327 | Algonquin, Ontario | Coleoptera - several | Insecta | Coleoptera | DS, unpublished | SC | Ad | A | Unknown |
| 1980 | November | 327 | Algonquin, Ontario | Lepidoptera - 4 unidentified larvae | Insecta | Lepidoptera | DS, unpublished | SC | Ad | A | Unknown |
| 1980 | November | 327 | Algonquin, Ontario | Lepidoptera - 1 Geometrid larva | Insecta | Lepidoptera | DS, unpublished | SC | Ad | A | Unknown |
| 1980 | November | 333 | Algonquin, Ontario | Diplopoda - 1 unidentified | Diplopoda | Unknown | DS, unpublished | SC | Ad | A | Unknown |
| 1980 | November | 333 | Algonquin, Ontario | Lepidoptera - 2 Geometridae | Insecta | Lepidoptera | DS, unpublished | SC | Ad | A | Unknown |
| 1980 | November | 333 | Algonquin, Ontario | Hymenoptera - 1 Formicidae | Insecta | Hymenoptera | DS, unpublished | SC | Ad | A | Unknown |
| 1981 | November | 334 | Ignace, Ontario | Orthoptera - several (Melanoplus?) | Insecta | Orthoptera | DS, unpublished | SC | Ad | A | Unknown |
| Not given | Winter | 334 | Gogama, Ontario | Coleoptera - 1 thorax of beetle | Insecta | Coleoptera | DS, unpublished | SC | Ad | A | Unknown |
| 1981 | December | 336 | Ignace, Ontario | Orthoptera - Several Acrididae | Insecta | Orthoptera | DS, unpublished | SC | Ad | A | Unknown |
| 1926 | December | 337 | British Columbia | Insect fragments | Insecta | Unknown | Munro in Bent 1946 | SC | Ad | A | Unknown |
| 1982 | December | 340 | Gogama, Ontario | Hymenoptera - 1 Formicidae | Insecta | Hymenoptera | DS, unpublished | SC | Ad | A | Unknown |
| 1982 | December | 340 | Gogama, Ontario | Hemiptera - 1 Pentatomidae | Insecta | Hemiptera | DS, unpublished | SC | Ad | A | Unknown |
| 1982 | December | 340 | Gogama, Ontario | Lepidoptera - 1 larva | Insecta | Lepidoptera | DS, unpublished | SC | Ad | A | Unknown |
| 1982 | December | 340 | Gogama, Ontario | Lepidoptera - 1 Hemerobius sp larva | Insecta | Lepidoptera | DS, unpublished | SC | Ad | A | Unknown |
| 1981 | December | 340 | Temagami, Ontario | Coleoptera - 1 beetle thorax | Insecta | Coleoptera | DS, unpublished | SC | Ad | A | Unknown |
| 1981 | December | 340 | Temagami, Ontario | Diplopoda - 1 Julidae | Diplopoda | Julida | DS, unpublished | SC | Ad | A | Unknown |
| 1981 | December | 349 | Ignace, Ontario | Orthoptera - 2+ | Insecta | Orthoptera | DS, unpublished | SC | Ad | A | Unknown |
| Not given | Not given |  | Not given | 1000 eggs of "Forest tent-caterpillar" | Insecta | Lepidoptera | Moore 1904 | SC | Ad | A | Unknown |
| 1982 | January | 3 | Gogama, Ontario | Maianthemum stellatum (Smilacina stellata) seeds, Star-flowered False Solomon's Seal | Magnoliopsida | Asparagales | DS, unpublished | SC | Ad | P | Likely Cached |
| 1982 | January | 3 | Gogama, Ontario | Frageria sp seeds (= Fragaria) | Magnoliopsida | Rosales | DS, unpublished | SC | Ad | P | Likely Cached |
| 1982 | January | 3 | Gogama, Ontario | Vaccinium sp seeds | Magnoliopsida | Ericales | DS, unpublished | SC | Ad | P | Likely Cached |
| 1982 | January | 3 | Gogama, Ontario | Unidentified seeds | Unknown | Unknown | DS, unpublished | SC | Ad | P | Unknown |
| 1971 | January | 9 | Algonquin, Ontario | "A seed (partridgeberry [Mitchella repens]?) | Magnoliopsida | Gentianales | F.W. Schueler, unpublished ms | SC | Ad | P | Likely Cached |
| 1971 | January | 9 | Algonquin, Ontario | "Spruce needles, a small piece of moss" | Pinopsida | Pinales | F.W. Schueler, unpublished ms | SC | Ad | P | Likely Fresh |
| 1982 | January | 10 | Gogama, Ontario | Maianthemum canadense (Canada Mayflower) seeds | Magnoliopsida | Asparagales | DS, unpublished | SC | Ad | P | Likely Cached |
| 1982 | January | 10 | Gogama, Ontario | Vaccinium sp seeds | Magnoliopsida | Ericales | DS, unpublished | SC | Ad | P | Likely Cached |
| 1982 | January | 10 | Gogama, Ontario | Sorbus sp seeds | Magnoliopsida | Rosales | DS, unpublished | SC | Ad | P | Either possible |
| 1982 | January | 10 | Gogama, Ontario | Liliaceae sp seeds | Liliopsida | Liliales | DS, unpublished | SC | Ad | P | Likely Cached |
| 2020 | January | 20 | Algonquin, Ontario | Prunus sp. (Cherry) | Megalopsida | Rosales | M. Runtz (photo) | SC | Ad | P | Either possible |
| 1982 | January | 31 | Gogama, Ontario | Maianthemum stellatum (Smilacina stellata) seeds, Star-flowered False Solomon's Seal | Magnoliopsida | Asparagales | DS, unpublished | SC | Ad | P | Likely Cached |
| 1982 | January | 31 | Gogama, Ontario | Vaccinium sp seeds | Magnoliopsida | Ericales | DS, unpublished | SC | Ad | P | Likely Cached |
| 1982 | January | 31 | Gogama, Ontario | Unidentified seeds | Unknown | Unknown | DS, unpublished | SC | Ad | P | Unknown |
| 1983 | February | 44 | Gogama, Ontario | Maianthemum canadense (Canada Mayflower) seeds | Magnoliopsida | Asparagales | DS, unpublished | SC | Ad | P | Likely Cached |
| 1983 | February | 44 | Gogama, Ontario | Sorbus sp seeds | Magnoliopsida | Rosales | DS, unpublished | SC | Ad | P | Either possible |
| 1983 | February | 46 | Gogama, Ontario | Maianthemum canadense (Canada Mayflower) seeds | Magnoliopsida | Asparagales | DS, unpublished | SC | Ad | P | Likely Cached |
| 1983 | February | 46 | Gogama, Ontario | Vaccinium sp seeds | Magnoliopsida | Ericales | DS, unpublished | SC | Ad | P | Likely Cached |
| 1983 | March | 61 | Gogama, Ontario | Maianthemum canadense (Canada Mayflower) seeds | Magnoliopsida | Asparagales | DS, unpublished | SC | Ad | P | Likely Cached |
| 1983 | March | 61 | Gogama, Ontario | Maianthemum canadense (Canada Mayflower) seeds | Magnoliopsida | Asparagales | DS, unpublished | SC | Ad | P | Likely Cached |
| 1983 | March | 61 | Gogama, Ontario | Sorbus sp seeds | Magnoliopsida | Rosales | DS, unpublished | SC | Ad | P | Either possible |
| 1968 | March | 66 | La Vérendrye, Québec | Maianthemum trifolium (Smilacina trifolia) seeds Three-leaved False Solomon's Seal | Magnoliopsida | Asparagales | DS, unpublished | SC | Ad | P | Likely Cached |
| 1972 | March | 78 | Algonquin, Ontario | Maianthemum trifolium (Smilacina trifolia) 1 seed Three-leaved False Solomon's Seal | Magnoliopsida | Asparagales | DS, unpublished | SC | Ad | P | Likely Cached |
| 1970 | June | 158 | Algonquin, Ontario | Maianthemum trifolium (Smilacina trifolia) seeds Three-leaved False Solomon's Seal | Magnoliopsida | Asparagales | DS, unpublished | SC | Ad | P |  |
| 1867 | June | 159 | North Shore, Gulf of St. Lawrence | Vaccinium oxycoccos berries (small bog cranberry) | Magnoliopsida | Ericales | Couper in Lewis 1935 | SC | Ad | P |  |
| Not given | June | 164 | Québec | "Vaccinium" berries | Magnoliopsida | Ericales | Harper 1953 | SC | Ad | P |  |
| Not given | June | 164 | Labrador | Arctostaphylos - 20 berries | Magnoliopsida | Ericales | Clement in Todd 1963 | SC | Ad | P |  |
| Not given | June | 164 | Labrador | Empetrum - 1 berry | Magnoliopsida | Ericales | Clement in Todd 1963 | SC | Ad | P |  |
| Not given | June | 164 | Labrador | Vaccinium vitis-idaea - 1 berry | Magnoliopsida | Ericales | Clement in Todd 1963 | SC | Ad | P |  |
| 1945 | June | 174 | Sandhill Lake, Manitoba | "Seeds, Fruit" | ? | ? | ROM72659 | SC | Ad | P |  |
| 1833 | June | 178 | Labrador | "Leaves of fir trees" | Coniferopsida | Pinales | Audubon 1841 | SC | Ad | P |  |
| 1980 | July | 196 | Algonquin, Ontario | Prunus sp seeds | Magnoliopsida | Rosales | DS, unpublished | SC | Ad | P |  |
| 1980 | July | 196 | Algonquin, Ontario | Maianthemum canadense (Canada Mayflower) seeds | Magnoliopsida | Asparagales | DS, unpublished | SC | Ad | P |  |
| 1915 | July | 203 | Shecatica, southern Labrador | "Curlew Berries" (= Empetrum nigrum?) | Magnoliopsida | Ericales | ROM71415 | SC | Ad | P |  |
| 1927 | August | 219 | Mt. Arrowhead, BC | "Fruit seeds" | ? | ? | ROM1191 | SC | Ad | P |  |
| Not given | August | 241 | Labrador Coast | Blueberries | Magnoliopsida | Ericales | Sutton in Todd 1963 | SC | Ad | P |  |
| 1984 | September | 258 | Algonquin, Ontario | Possibly seeds | Unknown | Unknown | DS, unpublished | SC | Ad | P |  |
| 1950 | September | 259 | Canmore, Alberta | "Red berries" | ? | ? | ROM78616 | SC | Ad | P |  |
| 1939 | September | 263 | British Columbia | Rosaceae seeds | Magnoliopsida | Rosales | Munro 1945 | SC | Ad | P |  |
| 1978 | October | 284 | Algonquin, Ontario | Aralia hispida Vent - 112 seeds | Magnoliopsida | Apiales | DS, unpublished | SC | Ad | P |  |
| 1978 | October | 284 | Algonquin, Ontario | Maianthemum canadense Desf. (Canada Mayflower)- 13 seeds | Magnoliopsida | Asparagales | DS, unpublished | SC | Ad | P |  |
| 1972 | October | 285 | Algonquin, Ontario | Maianthemum trifolium (Smilacina trifolia) 25 seeds Three-leaved False Solomon's Seal | Magnoliopsida | Asparagales | DS, unpublished | SC | Ad | P |  |
| 1972 | October | 285 | Bracebridge, Ontario | Viburnum sp - 2 large seeds | Magnoliopsida | Dipsacales | DS, unpublished | SC | Ad | P |  |
| 1972 | October | 285 | Bracebridge, Ontario | Sambucus pubens Michx. - 1 seed | Magnoliopsida | Dipsacales | DS, unpublished | SC | Ad | P |  |
| 1977 | October | 288 | La Vérendrye, Québec | Maianthemum canadense Desf. (Canada Mayflower)- 3 seeds | Magnoliopsida | Asparagales | DS, unpublished | SC | Ad | P |  |
| 1977 | October | 288 | La Vérendrye, Québec | Viburnum sp - 27 seeds | Magnoliopsida | Dipsacales | DS, unpublished | SC | Ad | P |  |
| 1981 | October | 288 | Ignace, Ontario | Maianthemum canadense (Canada Mayflower) seeds | Magnoliopsida | Asparagales | DS, unpublished | SC | Ad | P |  |
| 1981 | October | 288 | Ignace, Ontario | Maianthemum stellatum (Smilacina stellata) seeds, Star-flowered False Solomon's Seal | Magnoliopsida | Asparagales | DS, unpublished | SC | Ad | P |  |
| 1981 | October | 288 | Ignace, Ontario | Prunus sp seeds | Magnoliopsida | Rosales | DS, unpublished | SC | Ad | P |  |
| 1981 | October | 288 | Ignace, Ontario | Vaccinium sp seeds | Magnoliopsida | Ericales | DS, unpublished | SC | Ad | P |  |
| 1981 | October | 288 | Ignace, Ontario | Maianthemum canadense (Canada Mayflower) seeds | Magnoliopsida | Asparagales | DS, unpublished | SC | Ad | P |  |
| 1929 | October | 289 | Deer Lodge, Manitoba | "Acorn" |  |  | ROM79388 | SC | Ad | P |  |
| 1979 | October | 289 | Algonquin, Ontario | Maianthemum stellatum (Smilacina stellata) 9 seeds, Star-flowered False Solomon's Seal | Magnoliopsida | Asparagales | DS, unpublished | SC | Ad | P |  |
| 1979 | October | 289 | Algonquin, Ontario | Unidentified seeds - 3 | Unknown | Unknown | DS, unpublished | SC | Ad | P |  |
| 1901 | October | 290 | Muskoka, Ontario | "Seeds" | Unknown | Unknown | ROM69578 | SC | Ad | P |  |
| 1979 | October | 292 | Algonquin, Ontario | Unidentified seed - 1 | Unknown | Unknown | DS, unpublished | SC | Ad | P |  |
| 1981 | October | 299 | Ignace, Ontario | Alnus sp seeds | Magnoliopsida | Fagales | DS, unpublished | SC | Ad | P |  |
| 1981 | October | 299 | Ignace, Ontario | Vaccinium sp seeds | Magnoliopsida | Ericales | DS, unpublished | SC | Ad | P |  |
| 1972 | October | 300 | Algonquin, Ontario | Maianthemum trifolium (Smilacina trifolia) seeds Three-leaved False Solomon's Seal | Magnoliopsida | Asparagales | DS, unpublished | SC | Ad | P |  |
| 1896 | October | 301 | Port Sydney, Ontario | "Seeds" | Unknown | Unknown | ROM1211231169 | SC | Ad | P |  |
| 1983 | October | 302 | Algonquin, Ontario | Possibly seeds | Unknown | Unknown | DS, unpublished | SC | Ad | P |  |
| 1977 | October | 303 | Algonquin, Ontario | Maianthemum canadense Desf. (Canada Mayflower)- 1 seed | Magnoliopsida | Asparagales | DS, unpublished | SC | Ad | P |  |
| 1977 | October | 303 | Algonquin, Ontario | Ilex verticillata (L) Grey - 10 seeds | Magnoliopsida | Aquifoliales | DS, unpublished | SC | Ad | P |  |
| Not given | Fall? |  | Maine | Maianthemum stellatum (Smilacina stellata) seeds, Star-flowered False Solomon's Seal | Unknown | Unknown | Hardy in Bendire 1895 | SC | Ad | P |  |
| Not given | Fall? |  | Maine | Vaccinium sp seeds | Magnoliopsida | Rosales | Hardy in Bendire 1895 | SC | Ad | P |  |
| 1981 | November | 310 | Ignace, Ontario | Maianthemum canadense (Canada Mayflower) seeds | Magnoliopsida | Asparagales | DS, unpublished | SC | Ad | P | Either Possible |
| 1981 | November | 310 | Ignace, Ontario | Viburnum sp seeds | Magnoliopsida | Ericales | DS, unpublished | SC | Ad | P | Likely Fresh |
| 1979 | November | 310 | Algonquin, Ontario | Maianthemum canadense (Canada Mayflower) seeds | Magnoliopsida | Asparagales | DS, unpublished | SC | Ad | P | Either Possible |
| 1979 | November | 310 | Algonquin, Ontario | Viburnum sp seeds | Magnoliopsida | Dipsacales | DS, unpublished | SC | Ad | P | Either Possible |
| 1984 | November | 311 | Algonquin, Ontario | Possibly seeds | Unknown | Unknown | DS, unpublished | SC | Ad | P | Unknown |
| 1981 | November | 314 | Ignace, Ontario | Maianthemum canadense (Canada Mayflower) seeds | Magnoliopsida | Asparagales | DS, unpublished | SC | Ad | P | Either Possible |
| 1981 | November | 314 | Ignace, Ontario | Maianthemum stellatum (Smilacina stellata) seeds, Star-flowered False Solomon's Seal | Magnoliopsida | Asparagales | DS, unpublished | SC | Ad | P | Either Possible |
| 1981 | November | 314 | Ignace, Ontario | Vaccinium sp seeds | Magnoliopsida | Ericales | DS, unpublished | SC | Ad | P | Either Possible |
| 1972 | November | 315 | Algonquin, Ontario | Seeds | Unknown | Unknown | R. J. Rutter, pers. comm. | SC | Ad | P | Unknown |
| 1972 | November | 315 | Algonquin, Ontario | "Seeds, probably of a fruit" | Unknown | Unknown | R. J. Rutter, pers. comm. | SC | Ad | P | Unknown |
| Not given | November | 319 | Wisconsin | Rhus typhina - 5 seeds | Magnoliopsida | Sapindales | Bennetts 1900 | SC | Ad | P | Likely Fresh |
| Not given | November | 319 | Wisconsin | Unknown seeds - 10 | Unknown | Unknown | Bennetts 1900 | SC | Ad | P | Unknown |
| 1981 | November | 319 | Temagami (Cassels twp), Ontario | Maianthemum canadense (Canada Mayflower) seeds | Magnoliopsida | Asparagales | DS, unpublished | SC | Ad | P | Likely Cached |
| 1981 | November | 319 | Temagami (Cassels twp), Ontario | Vaccinium sp seeds | Magnoliopsida | Ericales | DS, unpublished | SC | Ad | P | Likely Cached |
| 1981 | November | 319 | Temagami (Cassels twp), Ontario | Carex sp seeds | Magnoliopsida | Poales | DS, unpublished | SC | Ad | P | Either Possible |
| 1981 | November | 319 | Temagami (Cassels twp), Ontario | Maianthemum canadense (Canada Mayflower) seeds | Magnoliopsida | Asparagales | DS, unpublished | SC | Ad | P | Likely Cached |
| Not given | November | 319 | New York | Seeds | Unknown | Unknown | Wilson and Bonaparte 1831 | SC | Ad | P | Unknown |
| 1981 | November | 322 | Ignace, Ontario | Aralia hispida seeds | Magnoliopsida | Apiales | DS, unpublished | SC | Ad | P | Either Possible |
| 1981 | November | 322 | Ignace, Ontario | Alnus sp seeds | Magnoliopsida | Fagales | DS, unpublished | SC | Ad | P | Likely Fresh |
| 1981 | November | 322 | Ignace, Ontario | Carex sp seeds | Magnoliopsida | Poales | DS, unpublished | SC | Ad | P | Either Possible |
| 1981 | November | 322 | Ignace, Ontario | Maianthemum stellatum (Smilacina stellata) seeds, Star-flowered False Solomon's Seal | Magnoliopsida | Asparagales | DS, unpublished | SC | Ad | P | Likely Cached |
| 1981 | November | 322 | Ignace, Ontario | Sorbus sp seeds | Magnoliopsida | Rosales | DS, unpublished | SC | Ad | P | Either Possible |
| 1981 | November | 322 | Ignace, Ontario | Vaccinium sp seeds | Magnoliopsida | Ericales | DS, unpublished | SC | Ad | P | Either Possible |
| 1981 | November | 322 | Ignace, Ontario | Maianthemum canadense (Canada Mayflower) seeds | Magnoliopsida | Asparagales | DS, unpublished | SC | Ad | P | Either Possible |
| 1982 | November | 326 | Chapleau, Ontario | Prunus sp seeds | Magnoliopsida | Rosales | DS, unpublished | SC | Ad | P | Either Possible |
| 1982 | November | 326 | Chapleau, Ontario | Maianthemum canadense (Canada Mayflower) seeds | Magnoliopsida | Asparagales | DS, unpublished | SC | Ad | P | Likely Cached |
| 1982 | November | 326 | Chapleau, Ontario | Sorbus sp seeds | Magnoliopsida | Rosales | DS, unpublished | SC | Ad | P | Either Possible |
| 1982 | November | 326 | Chapleau, Ontario | Prunus sp seeds | Magnoliopsida | Rosales | DS, unpublished | SC | Ad | P | Either Possible |
| 1982 | November | 326 | Chapleau, Ontario | Maianthemum canadense (Canada Mayflower) seeds | Magnoliopsida | Asparagales | DS, unpublished | SC | Ad | P | Likely Cached |
| 1981 | November | 334 | Ignace, Ontario | Maianthemum canadense (Canada Mayflower) seeds | Magnoliopsida | Asparagales | DS, unpublished | SC | Ad | P | Likely Cached |
| 1981 | November | 334 | Ignace, Ontario | Vaccinium sp seeds | Magnoliopsida | Ericales | DS, unpublished | SC | Ad | P | Likely Cached |
| 1981 | December | 336 | Ignace, Ontario | Maianthemum canadense (Canada Mayflower) seeds | Magnoliopsida | Asparagales | DS, unpublished | SC | Ad | P | Likley Cached |
| 1981 | December | 336 | Ignace, Ontario | Maianthemum stellatum (Smilacina stellata) seeds, Star-flowered False Solomon's Seal | Magnoliopsida | Asparagales | DS, unpublished | SC | Ad | P | Likely Cached |
| 1926 | December | 337 | British Columbia | Mostly seeds of Rosaceae | Magnoliopsida | Rosales | Munro in Bent 1946 | SC | Ad | P | Likely Cached |
| 1926 | December | 337 | British Columbia | A few seeds of serviceberry (Amelanchier sp) | Magnoliopsida | Rosales | Munro in Bent 1946 | SC | Ad | P | Either Possible |
| 1981 | December | 338 | Ignace, Ontario | Mostly seeds of Smilacina stellata (L.) Desf | Magnoliopsida | Asparagales | DS, unpublished | SC | Ad | P | Likely Cached |
| 1982 | December | 340 | Gogama, Ontario | Maianthemum canadense (Canada Mayflower) seeds | Magnoliopsida | Asparagales | DS, unpublished | SC | Ad | P | Likely Cached |
| 1982 | December | 340 | Gogama, Ontario | Prunus sp seeds | Magnoliopsida | Rosales | DS, unpublished | SC | Ad | P | Either Possible |
| 1972 | December | 341 | Algonquin, Ontario | Seeds "probably of a fruit" | Unknown | Unknown | R. J. Rutter, pers. comm. | SC | Ad | P | Unknown |
| 1972 | December | 341 | Algonquin, Ontario | Seeds | Unknown | Unknown | DS, unpublished | SC | Ad | P | Unknown |
| 1981 | December | 346 | Gogama?, Ontario | Maianthemum canadense (Canada Mayflower) seeds | Magnoliopsida | Asparagales | DS, unpublished | SC | Ad | P | Likely Cached |
| 1981 | December | 346 | Gogama?, Ontario | Prunus sp seeds | Magnoliopsida | Rosales | DS, unpublished | SC | Ad | P | Either Possible |
| 1981 | December | 346 | Gogama?, Ontario | Liliaceae seeds ? | Liliopsida | Liliales | DS, unpublished | SC | Ad | P | Likely Cached |
| 1981 | December | 349 | Ignace, Ontario | Maianthemum stellatum (Smilacina stellata) seeds, Star-flowered False Solomon's Seal | Magnoliopsida | Asparagales | DS, unpublished | SC | Ad | P | Likely Cached |
| 1981 | December | 349 | Ignace, Ontario | Vaccinium sp seeds | Magnoliopsida | Ericales | DS, unpublished | SC | Ad | P | Likely Cached |
| 1981 | December | 352 | Nipissing, Ontario | Carex sp seeds | Magnoliopsida | Poales | DS, unpublished | SC | Ad | P | Either Possible |
| 1981 | December | 352 | Nipissing, Ontario | Viburnum sp seeds | Magnoliopsida | Dipsacales | DS, unpublished | SC | Ad | P | Likely Fresh |
| 1982 | January | 3 | Gogama, Ontario | Mammal jaw - Clethrionomys? | Mammalia | Rodentia | DS, unpublished | SC | Ad | V | Either Possible |
| 1982 | January | 3 | Gogama, Ontario | Flesh, pehaps small mammal stomach | Mammalia | Unknown | DS, unpublished | SC | Ad | V | Either Possible |
| 1971 | January | 9 | Algonquin, Ontario | "Isolated limbbones of a young small mammal (mouse or shrew)" | Mammalia | Unknown | F.W. Schueler, unpublished ms | SC | Ad | V | Either Possible |
| 1982 | January | 17 | Gogama, Ontario | Short bristly hair in "furballs" perhaps Sorex sp | Mammalia | Eulipotyphla | DS, unpublished | SC | Ad | V | Either Possible |
| 1982 | January | 19 | Gogama, Ontario | Flesh and hair (Lepus)? | Mammalia | Lagomorpha | DS, unpublished | SC | Ad | V | Either Possible |
| 1982 | January | 31 | Gogama, Ontario | Short bristly hair (Sorex?) | Mammalia | Eulipotyphla | DS, unpublished | SC | Ad | V | Either Possible |
| 1983 | February | 46 | Gogama, Ontario | Bones of small mammal | Mammalia | Unknown | DS, unpublished | SC | Ad | V | Either Possible |
| 1983 | March | 61 | Gogama, Ontario | Small microtine - Clethrionomys? | Mammalia | Rodentia | DS, unpublished | SC | Ad | V | Either Possible |
| 1972 | March | 78 | Algonquin, Ontario | Many bones and hair of rodent | Mammalia | Rodentia | DS, unpublished | SC | Ad | V | Either Possible |
| 1979 | July | 193 | Algonquin, Ontario | Vertebrate flesh - may be amphibian | Amphibia? | Unknown | DS, unpublished | SC | Ad | V |  |
| 1980 | October | 283 | Algonquin, Ontario | Insectivora - 1 Sorex sp | Mammalia | Eulipotyphla | DS, unpublished | SC | Ad | V |  |
| 1981 | October | 288 | Ignace, Ontario | Short bristly hair and vertebrae (Sorex?) | Mammalia | Eulipotyphla | DS, unpublished | SC | Ad | V |  |
| 1981 | October | 299 | Ignace, Ontario | Bones and skin of a small mammal (Insectivore) | Mammalia | Eulipotyphla | DS, unpublished | SC | Ad | V |  |
| 1981 | October | 299 | Ignace, Ontario | Half shell of a small mollusc | (Mollusca) | Unknown | DS, unpublished | SC | Ad | V |  |
| 1981 | November | 310 | Ignace, Ontario | Skin and bone (microtine)? | Mammalia | Rodentia | DS, unpublished | SC | Ad | V | Either Possible |
| 1981 | November | 314 | Ignace, Ontario | Small microtine? - skin and bones | Mammalia | Rodentia | DS, unpublished | SC | Ad | V | Either Possible |
| 1972 | November | 315 | Algonquin, Ontario | "Bones" | (Vertebrate) | Unknown | R. J. Rutter, pers. comm. | SC | Ad | V | Either Possible |
| 1981 | November | 318 | Algonquin, Ontario | Remains and bones of a small mammal | Mammalia | Unknown | DS, unpublished | SC | Ad | V | Either Possible |
| 1981 | November | 319 | Temagami (Cassels twp), Ontario | Small mammal - Sorex sp - Lower jaw and bones | Mammalia | Eulipotyphla | DS, unpublished | SC | Ad | V | Either Possible |
| 1981 | November | 319 | Temagami (Cassels twp), Ontario | Bones and feathers of a small bird | Aves | Unknown | DS, unpublished | SC | Ad | V | Either Possible |
| Not given | November | 319 | New York | Bones of some batrachian - probably tree frog | Amphibia | Anura | Wilson and Bonaparte 1831 | SC | Ad | V | Either Possible |
| 1899 | November | 319 | Barron Co. Wisconsin | "Bones of some batrachian-probably treefrog | Amphibia | Anura | Bennetts 1900 | SC | Ad | V | Either Possible |
| 1982 | November | 326 | Chapleau, Ontario | Hair of small mammal | Mammalia | Unknown | DS, unpublished | SC | Ad | V | Either Possible |
| Not given | Late November | 326 | New York | Mollusc shell fragments | (Mollusca) | Unknown | Wilson and Bonaparte 1830 | SC | Ad | V | Either Possible |
| 1981 | November | 334 | Ignace, Ontario | Hair (microtine?) | Mammalia | Rodentia | DS, unpublished | SC | Ad | V | Either Possible |
| 1981 | December | 336 | Ignace, Ontario | Bones of small mammal (microtine?) | Mammalia | Rodentia | DS, unpublished | SC | Ad | V | Either Possible |
| 1981 | December | 338 | Ignace, Ontario | A few feathers (Gray Jay) | Aves | Passeriformes | DS, unpublished | SC | Ad | V | Either Possible |
| 1981 | December | 340 | Temagami, Ontario | Fat and/or flesh | (Vertebrate) | Unknown | DS, unpublished | SC | Ad | V | Either Possible |
| 1972 | December | 341 | Algonquin, Ontario | Bones | (Vertebrate) | Unknown | R. J. Rutter, pers. comm. | SC | Ad | V | Either Possible |
| 1972 | December | 341 | Algonquin, Ontario | Bones | (Vertebrate) | Unknown | DS, unpublished | SC | Ad | V | Either Possible |
| 1981 | December | 346 | Gogama?, Ontario | Fat, flesh | (Vertebrate) | Unknown | DS, unpublished | SC | Ad | V | Either Possible |
| 1981 | December | 349 | Ignace, Ontario | small mammal femur, tibula, humerus | Mammalia | Unknown | DS, unpublished | SC | Ad | V | Either Possible |
| 1981 | December | 352 | Nipissing, Ontario | Bones, fur, skin - Blarina? | Mammalia | Eulipotyphla | DS, unpublished | SC | Ad | V | Either Possible |
| 1981 | December | 352 | Nipissing, Ontario | Flesh, bone fragments and hair (microtine?) | Mammalia | Rodentia | DS, unpublished | SC | Ad | V | Either Possible |
| 1977 | April | 113 | Algonquin, Ontario | Lepidoptera - Geometridae - 24 Protoboarmia porcelaria | Insecta | Lepidoptera | DS, unpublished | SC | Ne | A | Unknown |
| 1977 | April | 113 | Algonquin, Ontario | Lepidoptera - Geometridae - Euchlaena sp probably marginata (Minot) | Insecta | Lepidoptera | DS, unpublished | SC | Ne | A | Unknown |
| 1977 | April | 113 | Algonquin, Ontario | Lepidoptera - 2 Geometridae sp headless larvae (not Protoboarmia) | Insecta | Lepidoptera | DS, unpublished | SC | Ne | A | Unknown |
| 1977 | April | 113 | Algonquin, Ontario | Lepidoptera - Noctuidae - 2 Pseudaletia unipuncta (Haw.) | Insecta | Lepidoptera | DS, unpublished | SC | Ne | A | Unknown |
| 1977 | April | 113 | Algonquin, Ontario | Lepidoptera - Noctuidae - 2 Polia sp probably purpurissata (Grt.) | Insecta | Lepidoptera | DS, unpublished | SC | Ne | A | Unknown |
| 1977 | April | 113 | Algonquin, Ontario | Lepidoptera - Noctuidae - 2 Hadeninae sp | Insecta | Lepidoptera | DS, unpublished | SC | Ne | A | Unknown |
| 1977 | April | 113 | Algonquin, Ontario | Lepidoptera - Noctuidae - 1 Noctuinae sp | Insecta | Lepidoptera | DS, unpublished | SC | Ne | A | Unknown |
| 1977 | April | 113 | Algonquin, Ontario | Diptera - Syrphidae - 27 Syrphinae | Insecta | Diptera | DS, unpublished | SC | Ne | A | Unknown |
| 1977 | April | 113 | Algonquin, Ontario | Diptera - Syrphidae - 2 Eristalini | Insecta | Diptera | DS, unpublished | SC | Ne | A | Unknown |
| 1977 | April | 113 | Algonquin, Ontario | Diptera - Tipulidae - 1 Tipula sp | Insecta | Diptera | DS, unpublished | SC | Ne | A | Unknown |
| 1977 | April | 113 | Algonquin, Ontario | Diptera - Tabanidae - 3 Hybomitra sp | Insecta | Diptera | DS, unpublished | SC | Ne | A | Unknown |
| 1977 | April | 113 | Algonquin, Ontario | Coleoptera - Dysticidae - 2 Ilybius sp (larvae) | Insecta | Coleoptera | DS, unpublished | SC | Ne | A | Unknown |
| 1977 | April | 113 | Algonquin, Ontario | Coleoptera - Carabidae - 1 Pterostichus adstrictus Esch. or pennsylvanicus Le C. (1 head) | Insecta | Coleoptera | DS, unpublished | SC | Ne | A | Either Possible |
| 1977 | April | 113 | Algonquin, Ontario | Coleoptera - 2 Carabidae (elytron and fragment) | Insecta | Coleoptera | DS, unpublished | SC | Ne | A | Unknown |
| 1977 | April | 113 | Algonquin, Ontario | Coleoptera - Curculionidae - Otiorhyuchus ovatus (L.) (strawberry root weevil) | Insecta | Coleoptera | DS, unpublished | SC | Ne | A | Likely Cached |
| 1977 | April | 113 | Algonquin, Ontario | Coleoptera - Curculionidae - 1 Weevil (?) elytron | Insecta | Coleoptera | DS, unpublished | SC | Ne | A | Unknown |
| 1977 | April | 113 | Algonquin, Ontario | Spider - 1 Xysticus sp | Arachnida | Araneae | DS, unpublished | SC | Ne | A | Unknown |
| 1977 | April | 113 | Algonquin, Ontario | Spider - 1 Pachygnatha tristriata C. L. Koch | Arachnida | Araneae | DS, unpublished | SC | Ne | A | Either Possible |
| 1977 | April | 113 | Algonquin, Ontario | Spider - 1 Trochosa terricola (Thorell) | Arachnida | Araneae | DS, unpublished | SC | Ne | A | Either Possible |
| 1977 | April | 114 | Algonquin, Ontario | Lepidoptera - Noctuidae - 7 Polis sp probably purpussiata (Grt.) | Insecta | Lepidoptera | DS, unpublished | SC | Ne | A | Unknown |
| 1977 | April | 114 | Algonquin, Ontario | Lepidoptera - 1 Actiidae sp | Insecta | Lepidoptera | DS, unpublished | SC | Ne | A | Unknown |
| 1977 | April | 114 | Algonquin, Ontario | Coleoptera - 1 Carabidae fragment | Insecta | Coleoptera | DS, unpublished | SC | Ne | A | Unknown |
| 1977 | April | 114 | Algonquin, Ontario | Spider - Amaurobiidae - 1 Trochosa terricola (Thorell) | Arachnida | Araneae | DS, unpublished | SC | Ne | A | Either Possible |
| 1983 | April | 114 | Algonquin, Ontario | Lepidoptera - 1 Citheroniidae caterpillar | Insecta | Lepidoptera | DS, unpublished | SC | Ne | A | Unknown |
| 1983 | April | 114 | Algonquin, Ontario | Lepidoptera - several Arctiidae caterpillars | Insecta | Lepidoptera | DS, unpublished | SC | Ne | A | Unknown |
| 1983 | April | 114 | Algonquin, Ontario | Hemiptera - 1+ Pentatomidae | Insecta | Hemiptera | DS, unpublished | SC | Ne | A | Unknown |
| 1983 | April | 114 | Algonquin, Ontario | Plecoptera - 1 nymph | Insecta | Plecoptera | DS, unpublished | SC | Ne | A | Unknown |
| 1983 | April | 114 | Algonquin, Ontario | Several aquatic bugs and beetles | Insecta | Unknown | DS, unpublished | SC | Ne | A | Unknown |
| 1983 | April | 114 | Algonquin, Ontario | Severla aquatic bugs and beetles | Insecta | Coleoptera | DS, unpublished | SC | Ne | A | Unknown |
| 1979 | April | 118 | Algonquin, Ontario | Coleoptera - 1 Carabid near Pterostichus | Insecta | Coleoptera | DS, unpublished | SC | Ne | A | Unknown |
| 1979 | April | 118 | Algonquin, Ontario | Coleoptera - 1 Dysticid near Agabus | Insecta | Coleoptera | DS, unpublished | SC | Ne | A | Unknown |
| 1979 | April | 118 | Algonquin, Ontario | Coleoptera - 10 Donacia | Insecta | Coleoptera | DS, unpublished | SC | Ne | A | Likely Cached |
| 1979 | April | 118 | Algonquin, Ontario | Coleoptera - 1 wireworm | Insecta | Coleoptera | DS, unpublished | SC | Ne | A | Unknown |
| 1968 | April | 119 | La Vérendrye, Québec | Insects (mostly beetles) and at least one spider | Insecta | Unknown | DS, unpublished | SC | Ne | A | Unknown |
| 1968 | April | 119 | La Vérendrye, Québec | Insects (mostly beetles) and at least one spider | Insecta | Coleoptera | DS, unpublished | SC | Ne | A | Unknown |
| 1968 | April | 119 | La Vérendrye, Québec | Insects (mostly beetles) and at least one spider | Insecta | Araneae | DS, unpublished | SC | Ne | A | Unknown |
| 1981 | April | 119 | Algonquin, Ontario | Lepidoptera - 2 larvae | Insecta | Lepidoptera | DS, unpublished | SC | Ne | A | Unknown |
| 1981 | April | 119 | Algonquin, Ontario | Lepidoptera - several moths | Insecta | Lepidoptera | DS, unpublished | SC | Ne | A | Unknown |
| 1981 | April | 119 | Algonquin, Ontario | Coleoptera - 2 Carabidae | Insecta | Coleoptera | DS, unpublished | SC | Ne | A | Unknown |
| 1981 | April | 119 | Algonquin, Ontario | Hemiptera - 1 Pentatomidae | Insecta | Hemiptera | DS, unpublished | SC | Ne | A | Unknown |
| 1981 | April | 119 | Algonquin, Ontario | Diptera - 1 Tabanid larva (Hybomitra sp) | Insecta | Diptera | DS, unpublished | SC | Ne | A | Unknown |
| 1968 | May | 121 | La Vérendrye, Québec | Odonata - 1 Libellula sp nymph | Insecta | Odonata | DS, unpublished | SC | Ne | A | Unknown |
| 1968 | May | 121 | La Vérendrye, Québec | Lepidoptera - 1 Noctuidae sp | Insecta | Lepidoptera | DS, unpublished | SC | Ne | A | Unknown |
| 1968 | May | 121 | La Vérendrye, Québec | Coleoptera - 1 Sphenophorus sp | Insecta | Coleoptera | DS, unpublished | SC | Ne | A | Unknown |
| 1968 | May | 121 | La Vérendrye, Québec | Coleoptera - 1 Agonum sp | Insecta | Coleoptera | DS, unpublished | SC | Ne | A | Unknown |
| 1968 | May | 121 | La Vérendrye, Québec | Coleoptera - 1 Semanotus ligneus | Insecta | Coleoptera | DS, unpublished | SC | Ne | A | Likely Fresh |
| 1968 | May | 121 | La Vérendrye, Québec | Spider - Araneida - Agelenidae - 1 Coras sp | Arachnida | Araneae | DS, unpublished | SC | Ne | A | Unknown |
| 1968 | May | 121 | La Vérendrye, Québec | Spider - Araneida - Amaurobiidae - 1 Trochosa terricola Thorell | Arachnida | Araneae | DS, unpublished | SC | Ne | A | Either Possible |
| 1968 | May | 121 | La Vérendrye, Québec | Spider - Araneida - Clubionidae - 1 Phrurotimpus sp possibly alarius Hentz | Arachnida | Araneae | DS, unpublished | SC | Ne | A | Either Possible |
| 1968 | May | 121 | La Vérendrye, Québec | Spider - Araneida - Thomisidae - 1 Philodromus sp, immature | Arachnida | Araneae | DS, unpublished | SC | Ne | A | Unknown |
| 1968 | May | 121 | La Vérendrye, Québec | Spider - Araneida - Lycosidae - Callioplus tibialis Emerton | Arachnida | Araneae | DS, unpublished | SC | Ne | A | Unknown |
| 1979 | May | 121 | Algonquin, Ontario | Coleoptera - Carabidae - 3 Pterostichus patruelis | Insecta | Coleoptera | DS, unpublished | SC | Ne | A | Likely Fresh |
| 1979 | May | 121 | Algonquin, Ontario | Coleoptera - Carabidae - 1 Agonum tenuecolle? | Insecta | Coleoptera | DS, unpublished | SC | Ne | A | Likely Fresh |
| 1979 | May | 121 | Algonquin, Ontario | Coleoptera - Carabidae - 4 Pterostichus | Insecta | Coleoptera | DS, unpublished | SC | Ne | A | Unknown |
| 1979 | May | 121 | Algonquin, Ontario | Coleoptera - 1 Curculionidae | Insecta | Coleoptera | DS, unpublished | SC | Ne | A | Unknown |
| 1979 | May | 121 | Algonquin, Ontario | Hymenoptera - 10 Formicidae | Insecta | Hymenoptera | DS, unpublished | SC | Ne | A | Unknown |
| 1979 | May | 121 | Algonquin, Ontario | Hymenoptera - 5 Apoidea | Insecta | Hymenoptera | DS, unpublished | SC | Ne | A | Unknown |
| 1979 | May | 121 | Algonquin, Ontario | Hymenoptera - 2 Ichneumonidae | Insecta | Hymenoptera | DS, unpublished | SC | Ne | A | Unknown |
| 1972 | May | 139 | Algonquin, Ontario | Coleoptera | Insecta | Coleoptera | DS, unpublished | SC | Ne | A | Unknown |
| 1972 | May | 139 | Algonquin, Ontario | Hymenoptera | Insecta | Hymenoptera | DS, unpublished | SC | Ne | A | Unknown |
| 1977 | April | 113 | Algonquin, Ontario | Spruce needle - 1 | Coniferopsida | Pinales | DS, unpublished | SC | Ne | P | Likely Fresh |
| 1977 | April | 113 | Algonquin, Ontario | Betula sp - 1 seed | Magnoliopsida | Fagales | DS, unpublished | SC | Ne | P | Either Possible |
| 1977 | April | 113 | Algonquin, Ontario | Sunflower - 1 seed | Magnoliopsida | Asterales | DS, unpublished | SC | Ne | P | Likely Cached |
| 1977 | April | 113 | Algonquin, Ontario | Grass - 1 seed | Magnoliopsida | Poales | DS, unpublished | SC | Ne | P | Likely Cached |
| 1972 | May | 139 | Algonquin, Ontario | Part of bone | (Vertebrate) | Unknown | DS, unpublished | SC | Ne | V | Either Possible |

**Literature Cited**

Addison, E. M., R. D. Strickland and D. J. H. Fraser. (1989). Gray Jays, *Perisoreus canadensis* and Common Ravens, *Corvus corvax*, as predators of winter ticks, *Dermacentor albipictus*. Can. Field Nat. no. 103:406-408.

Audubon, J. J. (1840-1844). The birds of America, Vol. 4. Dover Publications, 1967, New York.

Barnard, W. H. (1997). Juvenile Gray Jay preys upon Magnolia Warbler. J. Field Ornithol. no. 67: 252-253.

Beiswenger, R. E. (1981). Predation by grey jays on aggregating tadpoles of the Boreal Toad (*Bufo boreas*). Copeia no. 1981 (2):459-460.

Bendire, C. (1895). Life histories of North American birds, from the parrots to the grackles, with special reference to their breeding habits and eggs. U.S. Natl. Mus. Spec. Bull. no. 3.

Bennetts, W. J. (1900). Note on the food of the Canada Jay. Bull. Wis. Nat. Hist. Soc. no. 1:133.

Bent, A. C. (1946a). Life histories of North American jays, crows and titmice, Part I. U.S. Natl. Mus. Bull. no. 191.

Bottom of Form

Top of Form

Bent, A. C. (1946b). Life histories of north american jays, crows, and titmice, Pt. 2. U.S. Natl. Mus. Bull. no. 191.

Brewster, (1937). The birds of the Lake Umbagog region of Maine, pt. 3 Bull. Mus. Comp. Zool., vol. 66, pt. 3, pp. 408-521.

Earley, C. (2005). Gray Jay catching and eating dragonflies. Ontario Odonata no. 6:40.

Eifrig, C. W. G. (1906). Notes on some northern birds. Auk no. 23:313-318.

Fuirst, M., J. McLeod, D.R. Norris. (2022). Habitat preferences of adult Canada Jays (*Perisoreus canadensis*) during the post-breeding period in Algonquin Provincial Park, Ontario, Canada. Canadian Journal of Zoology 100: 355-362.

Gill, D. (1974). The Gray Jay as a predator of small mammals. Can. Field-Nat. no. 88:370-371.

Gilmore, D. (1996). Gray Jay captures mouse. Ontario Birds no. 14 (3):89-90.

Harper, F. (1953). Birds of the Nueltin Lake Expedition, Keewatin, 1947. Am. Mid. Nat. no. 49 (1):1-116.

Harper, F. (1958). Birds of the Ungava Peninsula. Univ. Kansas Mus. Nat. Hist. Misc. Publ. no. 17.

Hendricks, P. (2023). Canada jay predation of winter ticks (*Dermacentor albipictus*). Northwestern Nat. no. 104 (4):272-275.

Hendricks, P. and S. S. Pagano (2023). Canada Jay (*Perisoreus canadensis*) harvesting and caching fruits of Thin-leaved Snowberry (*Symphoricarpos albus*). Canadian Field-Naturalist 137(3): 000–000. <https://doi.org/10.22621/cfn.v137i3.3079>

Jewett, S. G., W. P. Taylor and J. W. Aldrich. (1953). Birds of Washington State. Seattle: Univ. of Washington Press.

Kingery, H. E. and U. C. Kingery. (1995). Gray Jay as predator on Cassin's finch nestlings. C.F.O. Journal no. 29 (1):17.

Lawrence, L. de K. (1947). Five days with a pair of nesting Canada Jays. Can. Field-Nat. no. 61:1-11.

Lawrence, L. de K. (1968). Notes on hoarding nesting material, display, and flycatching in the Gray Jay (*Perisoreus canadensis*). Auk no. 85:139.

Lesher, F. and J. Lesher. (1984). Gray Jay takes live mammal. Loon no. 56: 72-73.

Lewis, H. F. (1935). William Couper's observations of birds of the Labrador Peninsula. Can. Field-Nat. no. 49:112-116.

Lewis, H. F. (1939). Notes on September birds along Ontario's sea coast. Can. Field Nat. no. 53:50-53.

Macior, L. W. (1959). Predation by Gray Jays on the young of the hoary bat. Flicker no. 31: 100.

Moore, W. H. (1904). The Canada Jay. The Ottawa Naturalist:142-144.

Munro. J. A. (1945). The Birds of the Cariboo Parklands, British Columbia. Canadian Journal of Research. 23: 17-103.

Murray, M. P., C. A. Pearl and R. B. Bury. (2005). Apparent predation by Gray Jays, *Perisoreus canadensis*, on Long-toed Salamanders, *Ambystoma macrodactylum*, in the Oregon Cascade Range. Canadian Field-Naturalist no. 119 (2):291-292.

Olyphant, J. C. (1976). What Four Gray Jays—Banded in Cook County, Minnesota—revealed. North American Bird Bander 1: 22-24.

Ouellet, H. (1970). Further observations on the food and predatory habits of the Gray Jay. Can. J. Zool. no. 48:327-330.

Pike, E. A. (1978). Probable predation of netted birds by the Gray Jay *Perisoreus canadensis*. Jack-Pine Warbler no. 56 (4):211-212.

Rutter, R. J. (1969). A contribution to the biology of the Gray Jay (*Perisoreus canadensis*). Can. Field-Nat. no. 83:300-316.

Schueler, FW. unpublished MS

Strickland, D. (1969). Écologie, comportement social et nidification du Geai Gris (*Perisoreus canadensis*). M.Sc. thesis, Univ. Montréal, Montréal.

Sutherland, J. B. and R. L. Crawford. (1979). Gray Jay feeding on slime mold. Murrelet no. 60:28.

Todd, W. E. C. (1963). Birds of the Labrador Peninsula and adjacent areas: A distributional list. Toronto: University of Toronto Press.

Tozer, D. C. and M. L. Allen. (2004). Adult Gray Jay captures an adult Black-capped Chickadee. Wilson Bulletin no. 116 (4):357-359.

Warren, O. B. (1899). A chapter in the life of the Canada Jay. Auk no. 16:12-19.

Wilson, A., and C. L. Bonaparte. (1830). American Ornithology, vol. 1

Wilson, A., and C. L. Bonaparte. (1831). American Ornithology, vol. 2
