# Supplementary material for: Fecal DNA metabarcoding helps characterize the Canada jay’s diet and confirms its reliance on stored food for winter survival and breeding
Source: PLoS One. 2024 Apr 24;19(4):e0300583. doi: 10.1371/journal.pone.0300583 (PMC11042713; doi:10.1371/journal.pone.0300583)
Supplement: S3 File — Available at 10.6084/m9.figshare.25422847. (DOCX) [file pone.0300583.s005.docx]

#********************************************Main program section***********************************************

rm(list=ls())

library("ape", lib.loc="~/R/win-library/3.4")

library("ape", lib.loc="~/R/win-library/3.2")

# prompting to choose the folder location of the working directory with the input file to run the program

print("Choose the folder location where your input files are located")

Work_loc<-choose.dir()

#This is setting the directory to the working directory

Work_loc_0=Work_loc

setwd(Work_loc_0)

# prompting to choose the file of interest with the tab deliminted info

print("Choose your fasta file with the header format >ID|TaxaID|Family|Genus|Species|BOLD:Id")

list_file<-file.choose()

print(list_file)

# load in the data file in to the data for the lines

Seq_file<-data.frame(read.table(list_file,header=F,sep="\t",dec="."))

sink(file = paste(Work_loc, "\\run_output.txt", sep=""), append = FALSE)

#**********************************************************************************************************************************************************

#This section is taking the fasta file and formatting it in to columns to create the input file for the program

#taking the read in file and changing from Fasta to tab deliminted

Header0 <- Seq_file[seq(from = 1, to = nrow(Seq_file), by = 2), 1]

#Getting the unique identifier by pulling it off of the front of the fasta header

Header<-gsub("\\|.*", "", Header0)

#Header1 is the processID only (unique identifier)

Header1<-gsub("\\|.*", "", Header0)

Header1<-gsub(">","",Header1)

#also copying the header and pulling out the Family Header

Header2<-gsub("^.*?\\|","",Header0)

#print(Header2)

Header2<-gsub("^.*?\\|","",Header2)

#print(Header2)

Header2<-gsub("\\|.*", "", Header2)

#print(Header2)

#also copying the header and pulling out the Genus Header

Header3<-gsub("^.*?\\|","",Header0)

#print(Header3)

Header3<-gsub("^.*?\\|","",Header3)

#print(Header3)

Header3<-gsub("^.*?\\|","",Header3)

#print(Header3)

Header3<-gsub("\\|.*", "", Header3)

#print(Header3)

#also copying the header and pulling out the Species Header

Header4<-gsub("^.*?\\|","",Header0)

#print(Header4)

Header4<-gsub("^.*?\\|","",Header4)

#print(Header4)

Header4<-gsub("^.*?\\|","",Header4)

#print(Header4)

Header4<-gsub("^.*?\\|","",Header4)

#print(Header4)

Header4<-gsub("\\|.*", "", Header4)

#print(Header4)

#also copying the header and pulling out the BOLD BINs and creating another column to make the list more easily searchable

Header5<-gsub(".*\\|", "", Header0)

Sequence <- Seq_file[seq(from = 2, to = nrow(Seq_file), by = 2), 1]

New_Seq_file <- data.frame(Header,Header1,Header2,Header3,Header4,Sequence)

#******************MAKING THE DIRECTORY TO STORE THE OUTPUT FILES****************************************************

#Make a file folder to conatin all results from the program

file_folder_cmd_str<-paste("mkdir ",Work_loc, "\\",New_Seq_file[1,3],"_Barcodes_Gap_Calcs_Sp_Gen", sep="")

shell(file_folder_cmd_str)

Work_loc=paste(Work_loc, "\\",New_Seq_file[1,3],"_Barcodes_Gap_Calcs_Sp_Gen", sep="")

#Initiating the output file

x <- data.frame(Taxa="", Within="", Between="",stringsAsFactors=FALSE)

write.table(x,file=paste(Work_loc, "\\Barcodes_Gap_Calcs_Sp_Gen.txt", sep=""), na="", row.names=FALSE, col.names=TRUE, quote = FALSE,sep="\t", append=FALSE)

#**********************************************************************************************************************************************************

#Then need to create three distance matricies

#For each genus create new species table and run a distance matrix

#Get all unique genera from the data set

Species<-unique(New_Seq_file[[5]])

Genera<-unique(New_Seq_file[[4]])

Families<-unique(New_Seq_file[[3]])

#*****************************THIS SECTION IS REMOVING ********************

#Remove all entries where there is no species or genus name

#Need to sort the data frame by headers

Work_sp_gen_seq_file<-New_Seq_file[order(New_Seq_file$Header4),]

#Here I remove any species identifications which are non-existent or blank

Work_sp_gen_seq_file<-Work_sp_gen_seq_file[!(Work_sp_gen_seq_file$Header4==""),]

#Here I remove any genus identifications which are non-existent or blank

Work_sp_gen_seq_file<-Work_sp_gen_seq_file[!(Work_sp_gen_seq_file$Header3==""),]

#Get the list of all unique species in the data set

Species<-unique(Work_sp_gen_seq_file[[5]])

# In a loop for each unique species name count the number of entries in Header4 column.

for (c in 1:length(Species)){

# Check the number of sequences per taxon (species) by looping through, searching, and counting the Header4 column for each unique taxon(Species)

num_rows_with_target_species_name=nrow(Work_sp_gen_seq_file[c(Work_sp_gen_seq_file$Header4==Species[c]),])

try_to_get_taxa<-Work_sp_gen_seq_file[(Work_sp_gen_seq_file$Header4==Species[c]),]

print("Here is my try")

print(try_to_get_taxa)

print(paste("A Here is the number of rows w target sp:",Species[c], ":",num_rows_with_target_species_name, sep=""))

print(paste("B Here is the number of rows w target sp:",Work_sp_gen_seq_file[Species[c],"Header4"], ":",num_rows_with_target_species_name, sep=""))

#Check the above calculated number of entries and if equal to 1 indicating a single sequence for the taxon(species) then remove that taxon

if (num_rows_with_target_species_name==1){

Work_sp_gen_seq_file<-Work_sp_gen_seq_file[!(Work_sp_gen_seq_file$Header4==Species[c]),]

print("Removed target species w one entry")

}

}

#Here getting the number of Genera in the data set

Genera<-unique(Work_sp_gen_seq_file[[4]])

Genera

# In a loop for each unique genera name count the number of species in Header4 column.

for (d in 1:length(Genera)){

#first get the genus of target

Genera_subset<-Work_sp_gen_seq_file[Work_sp_gen_seq_file$Header3==Genera[d],]

# Check the number of species per genus by looping through, searching, and counting the Header4 column for the number of Species for each Genus

num_species_in_the_target_genus=length(unique(Genera_subset[[5]]))

print(paste("Here is the number of rows w target genus:",Genera[d], ":",num_species_in_the_target_genus, sep=""))

#Check the above calculated number of entries and if equal to 1 indicating a single species in the genus then remove that taxon

if (num_species_in_the_target_genus==1){

Work_sp_gen_seq_file<-Work_sp_gen_seq_file[!(Work_sp_gen_seq_file$Header3==Genera[d]),]

print("Removed target genus w one entry")

}

}

#Here getting the number of Genera in the data set

Genera<-unique(Work_sp_gen_seq_file[[4]])

#*******************************************LOOP ****************************************************

#This section will subset the data in the loop into each of the Genera present in the data set one at a time

for (i in 1:length(Genera)){

#Get the subset of data in the genus of interest for this loop

Genera_subset<-subset(Work_sp_gen_seq_file,Header3==Genera[i])

#This is setting up the data frame with the species names and sequences for the genus currently in the loop

Species_subset <- data.frame(Genera_subset["Header4"],Genera_subset["Sequence"])

#Flag to enter in the first addition to the matrix to make the fasta file

fasta_flag=0

for (j in 1:nrow(Species_subset)){

#This is setting up a flag so the first time we initialize the matrix and then the second time we rbind to the matrix for the fasta file format

if (fasta_flag==0){

final_matrix_for_file<-paste(">",Species_subset[j,1],sep="")

x<-as.character(Species_subset[j,2])

final_matrix_for_file<-rbind(final_matrix_for_file,x)

fasta_flag=1

}else{

x<-paste(">",Species_subset[j,1],sep="")

final_matrix_for_file<-rbind(final_matrix_for_file,x)

x<-as.character(Species_subset[j,2])

final_matrix_for_file<-rbind(final_matrix_for_file,x)

}

}

#Providing the name and the location where the output files will be located

Write_to_file_out_str<-paste(Work_loc,"\\TEST.fas", sep="")

write.table(final_matrix_for_file,file=Write_to_file_out_str,append=FALSE, na="", row.names=FALSE, col.names=FALSE, quote = FALSE,sep="\n")

#********Calculating distance matrix**************************************************************************************************************************************************

dat<-read.dna(Write_to_file_out_str, format="fasta")

dat_out<-dist.dna(dat, model = "raw", variance = FALSE, gamma = FALSE, pairwise.deletion = TRUE, base.freq = NULL, as.matrix = TRUE)

write.table(dat_out,file=paste(Work_loc,"\\Genus_results_",Genera[i],".txt",sep=""),row.names=TRUE, col.names=NA, quote = FALSE,sep="\t")

#********Calculating highest inter vs lowest intra values**************************************************************************************************************************************************

#get all unique column names

unique_col_or_row_names<-unique(colnames(dat_out))

temp_col_or_row_names<-as.vector(colnames(dat_out))

#***************************************Looping through each of the unique columns***************

for (k in 1:length(unique_col_or_row_names)){

#***************Getting the columns pulled out*************

flag=0

for (j in 1:ncol(dat_out)){

if (temp_col_or_row_names[j]!=unique_col_or_row_names[k]){

#the below if get the headers which we want to remove. The extra if is there to initiate the vector the first time and then to add

if(flag==0){

col_to_remove<-as.vector(-j)

flag=1

}else{

col_to_remove<-as.vector(c(col_to_remove,-j))

}

}

}

#*************Now getting the rows for intra**********

temp_matrix<-dat_out[,c(col_to_remove)]

flag=0

for (j in 1:ncol(dat_out)){

if (temp_col_or_row_names[j]!=unique_col_or_row_names[k]){

#the below if get the headers which we want to remove. The extra if is there to initiate the vector the first time and then to add

if(flag==0){

row_to_keep<-as.vector(-j)

row_to_remove<-as.vector(j)

flag=1

}else{

row_to_keep<-as.vector(c(row_to_keep,-j))

row_to_remove<-as.vector(c(row_to_remove,j))

}

}

}

row_to_use_for_intra<-temp_matrix[c(row_to_keep),]

row_to_use_for_inter<-temp_matrix[c(row_to_remove),]

#***************************************************************************

#pushing the results of this loop to the output file

x <- data.frame(Taxa=unique_col_or_row_names[k], Within=max(row_to_use_for_intra), Between=min(row_to_use_for_inter),AVGWithin=average(row_to_use_for_intra),AVGBetween=average(row_to_use_for_inter),stringsAsFactors=FALSE)

write.table(x,file=paste(Work_loc, "\\Barcodes_Gap_Calcs_Sp_Gen.txt", sep=""), na="", row.names=FALSE, col.names=FALSE, quote = FALSE,sep="\t", append=TRUE)

print(paste("Final results in format: Species Name: ", unique_col_or_row_names[k], " - Max within species: ",max(row_to_use_for_intra), " - Min between species: ",min(row_to_use_for_inter)))

} #closing of the loop statement getting the min within and max between values

} #closing of the main loop which obtains the list for the species and genera

sink()

#******************************************************************************************************************

#*****************************GEN_FAM******************************************************************************

#******************************************************************************************************************

#This is setting the directory to the working directory

Work_loc=Work_loc_0

#Make a file folder to conatin all results from the program

file_folder_cmd_str<-paste("mkdir ",Work_loc, "\\",New_Seq_file[1,3],"_Barcodes_Gap_Calcs_Gen_Fam", sep="")

shell(file_folder_cmd_str)

Work_loc=paste(Work_loc, "\\",New_Seq_file[1,3],"_Barcodes_Gap_Calcs_Gen_Fam", sep="")

#Initiating the output file

x <- data.frame(Genus="", Within="", Between="",stringsAsFactors=FALSE)

write.table(x,file=paste(Work_loc, "\\Barcodes_Gap_Calcs_Gen_Fam.txt", sep=""), na="", row.names=FALSE, col.names=TRUE, quote = FALSE,sep="\t", append=FALSE)

sink(file = paste(Work_loc, "\\run_output.txt", sep=""), append = FALSE)

#**********************************************************************************************************************************************************

#This section is taking the fasta file and formatting it in to columns to create the input file for the program

#taking the read in file and changing from Fasta to tab deliminted

Header0 <- Seq_file[seq(from = 1, to = nrow(Seq_file), by = 2), 1]

#Getting the unique identifier by pulling it off of the front of the fasta header

Header<-gsub("\\|.*", "", Header0)

#Header1 is the processID only (unique identifier)

Header1<-gsub("\\|.*", "", Header0)

Header1<-gsub(">","",Header1)

#also copying the header and pulling out the Family Header

Header2<-gsub("^.*?\\|","",Header0)

#print(Header2)

Header2<-gsub("^.*?\\|","",Header2)

#print(Header2)

Header2<-gsub("\\|.*", "", Header2)

#print(Header2)

#also copying the header and pulling out the Genus Header

Header3<-gsub("^.*?\\|","",Header0)

#print(Header3)

Header3<-gsub("^.*?\\|","",Header3)

#print(Header3)

Header3<-gsub("^.*?\\|","",Header3)

#print(Header3)

Header3<-gsub("\\|.*", "", Header3)

#print(Header3)

#also copying the header and pulling out the Species Header

Header4<-gsub("^.*?\\|","",Header0)

#print(Header4)

Header4<-gsub("^.*?\\|","",Header4)

#print(Header4)

Header4<-gsub("^.*?\\|","",Header4)

#print(Header4)

Header4<-gsub("^.*?\\|","",Header4)

#print(Header4)

Header4<-gsub("\\|.*", "", Header4)

#print(Header4)

#also copying the header and pulling out the BOLD BINs and creating another column to make the list more easily searchable

Header5<-gsub(".*\\|", "", Header0)

Sequence <- Seq_file[seq(from = 2, to = nrow(Seq_file), by = 2), 1]

New_Seq_file <- data.frame(Header,Header1,Header2,Header3,Header4,Sequence)

#**********************************************************************************************************************************************************

#Then need to create three distance matricies

#For each genus create new species table and run a distance matrix

#Get all unique genera from the data set

Species<-unique(New_Seq_file[[5]])

Genera<-unique(New_Seq_file[[4]])

Family<-unique(New_Seq_file[[3]])

#*****************************THIS SECTION IS REMOVING ********************

#Remove all entries where there is no species or genus name

#Need to sort the data frame by headers

Work_gen_fam_seq_file<-New_Seq_file[order(New_Seq_file$Header4),]

#Here I remove any genus identifications which are non-existent or blank

Work_gen_fam_seq_file<-Work_gen_fam_seq_file[!(Work_gen_fam_seq_file$Header3==""),]

#Here I remove any Family identifications which are non-existent or blank

Work_gen_fam_seq_file<-Work_gen_fam_seq_file[!(Work_gen_fam_seq_file$Header2==""),]

#Get the list of all unique Genera in the data set

Genera<-unique(New_Seq_file[[4]])

print("Here are the Genera present")

print(Genera)

# In a loop for each unique genera name count the number of species in Header3 column.

for (d in 1:length(Genera)){

#first get the genus of target

Genera_subset<-Work_gen_fam_seq_file[Work_gen_fam_seq_file$Header3==Genera[d],]

Genera_subset

print(paste("Here is the number of rows in ", nrow(Genera_subset)," genus:",Genera[d], sep=""))

#Check the above calculated number of entries and if equal to 1 indicating a single species in the genus then remove that taxon

if (nrow(Genera_subset)==1){

Work_gen_fam_seq_file<-Work_gen_fam_seq_file[!(Work_gen_fam_seq_file$Header3==Genera[d]),]

print("Removed target genus w one entry")

}

}

#Here getting the number of Family in the data set

Family<-unique(Work_gen_fam_seq_file[[3]])

# In a loop for each unique family name count the number of entries in Header3 column.

for (c in 1:length(Family)){

Family_subset<-Work_gen_fam_seq_file[Work_gen_fam_seq_file$Header2==Family[c],]

print("This is the Family subset")

print(Family_subset)

# Check the number of species per genus by looping through, searching, and counting the Header4 column for the number of Species for each Genus

num_species_in_the_target_Family=length(unique(Family_subset[[4]]))

print(paste("Here is the number of rows in ", nrow(Family_subset)," Family:",Family[c], sep=""))

#Check the above calculated number of entries and if equal to 1 indicating a single species in the genus then remove that taxon

if (num_species_in_the_target_Family==1){

print("This is the value of the Family I am trying to remove:")

print(Family[c])

Work_gen_fam_seq_file<-Work_gen_fam_seq_file[!(Work_gen_fam_seq_file$Header2==Family[c]),]

print("Removed target Family w one entry an here is the new value of the main file")

print(Work_gen_fam_seq_file)

}

}

#*******************************************LOOP ****************************************************

#This section will subset the data in the loop into each of the Genera present in the data set one at a time

#Get the list of all unique Families in the data set

Family<-unique(Work_gen_fam_seq_file[[3]])

for (i in 1:length(Family)){

#Get the subset of data in the Family of interest for this loop

Family_subset<-subset(Work_gen_fam_seq_file,Header2==Family[i])

print("Here is the family subset")

print(Family_subset)

#This is setting up the data frame with the species names and sequences for the genus currently in the loop

Genera_subset <- data.frame(Family_subset["Header3"],Family_subset["Sequence"])

print("Here is the Genera subset")

print(Genera_subset)

#Flag to enter in the first addition to the matrix to make the fasta file

fasta_flag=0

for (j in 1:nrow(Genera_subset)){

#This is setting up a flag so the first time we initialize the matrix and then the second time we rbind to the matrix for the fasta file format

if (fasta_flag==0){

final_matrix_for_file<-paste(">",Genera_subset[j,1],sep="")

x<-as.character(Genera_subset[j,2])

final_matrix_for_file<-rbind(final_matrix_for_file,x)

fasta_flag=1

}else{

x<-paste(">",Genera_subset[j,1],sep="")

final_matrix_for_file<-rbind(final_matrix_for_file,x)

x<-as.character(Genera_subset[j,2])

final_matrix_for_file<-rbind(final_matrix_for_file,x)

}

}

#Providing the name and the location where the output files will be located

Write_to_file_out_str<-paste(Work_loc,"\\TEST.fas", sep="")

write.table(final_matrix_for_file,file=Write_to_file_out_str,append=FALSE, na="", row.names=FALSE, col.names=FALSE, quote = FALSE,sep="\n")

print("Made it this far")

#********Calculating distance matrix****************************************************************************************************************************

dat<-read.dna(Write_to_file_out_str, format="fasta")

dat_out<-dist.dna(dat, model = "raw", variance = FALSE, gamma = FALSE, pairwise.deletion = TRUE, base.freq = NULL, as.matrix = TRUE)

write.table(dat_out,file=paste(Work_loc,"\\Genus_results_",Genera[i],".txt",sep=""),row.names=TRUE, col.names=NA, quote = FALSE,sep="\t")

#********Calculating highest inter vs lowest intra values**************************************************************************************************************************************************

#get all unique column names

unique_col_or_row_names<-unique(colnames(dat_out))

temp_col_or_row_names<-as.vector(colnames(dat_out))

print("Here is the dat_out")

print(dat_out)

#***************************************Looping through each of the unique columns***************

for (k in 1:length(unique_col_or_row_names)){

#***************Getting the columns pulled out*************

flag=0

for (j in 1:ncol(dat_out)){

if (temp_col_or_row_names[j]!=unique_col_or_row_names[k]){

#the below if get the headers which we want to remove. The extra if is there to initiate the vector the first time and then to add

if(flag==0){

col_to_remove<-as.vector(-j)

flag=1

}else{

col_to_remove<-as.vector(c(col_to_remove,-j))

}

}

}

#*************Now getting the rows for intra and inter**********

temp_matrix<-dat_out[,c(col_to_remove)]

flag=0

for (j in 1:ncol(dat_out)){

if (temp_col_or_row_names[j]!=unique_col_or_row_names[k]){

#the below if get the headers which we want to remove. The extra if is there to initiate the vector the first time and then to add

if(flag==0){

row_to_keep<-as.vector(-j)

row_to_remove<-as.vector(j)

flag=1

}else{

row_to_keep<-as.vector(c(row_to_keep,-j))

row_to_remove<-as.vector(c(row_to_remove,j))

}

}

}

print("Here is the temp matrix")

print(temp_matrix)

row_to_use_for_intra<-temp_matrix[c(row_to_keep),]

print("Here are the rows to keep and calcuate the within")

print(row_to_use_for_intra)

row_to_use_for_inter<-temp_matrix[c(row_to_remove),]

print("Here are the rows to keep and calcuate the between")

print(row_to_use_for_inter)

#***************************************************************************

#pushing the results of this loop to the output file

x <- data.frame(Genus=unique_col_or_row_names[k], Within=max(row_to_use_for_intra), Between=min(row_to_use_for_inter),stringsAsFactors=FALSE)

write.table(x,file=paste(Work_loc, "\\Barcodes_Gap_Calcs_Gen_Fam.txt", sep=""), na="", row.names=FALSE, col.names=FALSE, quote = FALSE,sep="\t", append=TRUE)

print(paste("Final results in format: Genus Name: ", unique_col_or_row_names[k], " - Max within species: ",max(row_to_use_for_intra), " - Min between species: ",min(row_to_use_for_inter)))

} #closing of the loop statement getting the min within and max between values

} #closing of the main loop which obtains the list for the species and genera

sink()
