# Supplementary material for: Fecal DNA metabarcoding helps characterize the Canada jay’s diet and confirms its reliance on stored food for winter survival and breeding
Source: PLoS One. 2024 Apr 24;19(4):e0300583. doi: 10.1371/journal.pone.0300583 (PMC11042713; doi:10.1371/journal.pone.0300583)
Supplement: Attachment — Submitted filename: PONE-D-23-24828_reviewer.pdf [file pone.0300583.s009.pdf]

PLOS ONE

Fecal DNA metabarcoding helps characterize Canada jay diet and confirms the reliance of stored food for winter survival and breeding

--Manuscript Draft--

**Manuscript Number:**	PONE-D-23-24828
**Article Type:**	Research Article
**Full Title:**	Fecal DNA metabarcoding helps characterize Canada jay diet and confirms the reliance of stored food for winter survival and breeding
**Short Title:**	Metabarcoding helps characterize Canada jay diet and confirms the use of stored food for survival and breeding
**Corresponding Author:**	Alex O Sutton<br>Bangor University<br>Bangor, Gwynedd UNITED KINGDOM
**Keywords:**	diet analysis; food hoarding; stomach content analysis; winter breeding
**Abstract:**	Accurately determining the diet of wild animals can be challenging if food items are small, visible only briefly, or are rendered visually unidentifiable in the digestive system. In some food caching species, an additional challenge is determining whether consumed diet items had been previously stored or are fresh. The Canada jay (Perisoreus canadensis) is a generalist resident of North American boreal and subalpine forests with anatomical and behavioural adaptations allowing it to make thousands of arboreal food caches in summer and fall that are presumably responsible for its high winter survival and propensity to begin breeding in the late winter. We first used DNA fecal metabarcoding to obtain novel information on nestling diet and then consolidated this information with all published and additional unpublished diet information throughout the year and across life stages. Using natural history information of diet items identified, we then estimated the extent to which food consumed by adults in the winter and nestlings in the late winter/early spring was 'likely cached' or 'fresh'. In total, our dataset included 662 observations of food items consumed by Canada jays. Of the 87 food items consumed by adults in the winter, 39% were classified as 'likely recovered' caches and 6% were deemed to be 'likely fresh'. For nestlings, 29% of 125 food items identified to species were 'likely recovered' and 38% were 'likely fresh'. Our results support both the indispensability of cached food for Canada jay winter survival and previous suggestions that cached food is important for late winter/early spring breeding. Our work highlights the value of combining metabarcoding and natural history observations to determine the temporal origins of food items consumed by individuals, which, for the Canada jay, will be integral for understanding how diet influences individual success and population dynamics.
**Order of Authors:**	Alex O Sutton
	Dan Strickland
	Jacob Lachapelle
	Robert G Young
	Robert Hanner
	Daniel F Brunton
	Jeffrey H Skevington
	Nikole E Freeman
	D Ryan Norris
**Additional Information:**	
**Question**	**Response**
**Financial Disclosure**	We would also like to thank NSERC (DRN, NEF), Ontario Parks (AOS, DRN, NEF),

Powered by Editorial Manager® and ProduXion Manager® from Aries Systems Corporation

<table>
<tr><td>Enter a financial disclosure statement that describes the sources of funding for the work included in this submission. Review the submission guidelines for detailed requirements. View published research articles from PLOS ONE for specific examples.

This statement is required for submission and will appear in the published article if the submission is accepted. Please make sure it is accurate.

Unfunded studies
Enter: The author(s) received no specific funding for this work.

Funded studies
Enter a statement with the following details:
• Initials of the authors who received each award
• Grant numbers awarded to each author
• The full name of each funder
• URL of each funder website
• Did the sponsors or funders play any role in the study design, data collection and analysis, decision to publish, or preparation of the manuscript?
• NO - Include this sentence at the end of your statement: The funders had no role in study design, data collection and analysis, decision to publish, or preparation of the manuscript.
• YES - Specify the role(s) played.

* typeset</td>
<td>Friends of Algonquin Park, Wildlife Conservation Society Canada (AOS, NEF), and the Ministry of Natural Resources and Forestry (AOS, DRN, NEF) for providing funding and logistical support for our project.</td></tr>
<tr><td>Competing Interests

Use the instructions below to enter a competing interest statement for this submission. On behalf of all authors, disclose any competing interests that could be perceived to bias this work—acknowledging all financial support and any other relevant financial or non-financial competing interests.

This statement is required for submission and will appear in the published article if</td>
<td>The authors have declared that no competing interests exist.</td></tr>
</table>

the submission is accepted. Please make sure it is accurate and that any funding sources listed in your Funding Information later in the submission form are also declared in your Financial Disclosure statement.<br><br>View published research articles from *PLOS ONE* for specific examples.<br><br>**NO authors have competing interests**<br><br>Enter: *The authors have declared that no competing interests exist.*<br><br>**Authors with competing interests**<br><br>Enter competing interest details beginning with this statement:<br><br>*I have read the journal's policy and the authors of this manuscript have the following competing interests: [insert competing interests here]*<br><br>* typeset	
**Ethics Statement**<br><br>Enter an ethics statement for this submission. This statement is required if the study involved:<br><br>• Human participants<br>• Human specimens or tissue<br>• Vertebrate animals or cephalopods<br>• Vertebrate embryos or tissues<br>• Field research<br><br>Write "N/A" if the submission does not require an ethics statement.<br><br>General guidance is provided below. Consult the submission guidelines for detailed instructions. **Make sure that all information entered here is included in the Methods section of the manuscript.**	All work with the Canada jays was approved by the University of Guelph Animal Care and Use Committee and Canadian Wildlife Services. Field research in Algonquin Provincial Park was approved by Ontario Parks and the Ontario Ministry of Natural Resources and Forestry.

Powered by Editorial Manager® and ProduXion Manager® from Aries Systems Corporation

**Format for specific study types**<br><br>**Human Subject Research (involving human participants and/or tissue)**<br>• Give the name of the institutional review board or ethics committee that approved the study<br>• Include the approval number and/or a statement indicating approval of this research<br>• Indicate the form of consent obtained (written/oral) or the reason that consent was not obtained (e.g. the data were analyzed anonymously)<br><br>**Animal Research (involving vertebrate animals, embryos or tissues)**<br>• Provide the name of the Institutional Animal Care and Use Committee (IACUC) or other relevant ethics board that reviewed the study protocol, and indicate whether they approved this research or granted a formal waiver of ethical approval<br>• Include an approval number if one was obtained<br>• If the study involved *non-human primates*, add *additional details* about animal welfare and steps taken to ameliorate suffering<br>• If anesthesia, euthanasia, or any kind of animal sacrifice is part of the study, include briefly which substances and/or methods were applied<br><br>**Field Research**<br><br>Include the following details if this study involves the collection of plant, animal, or other materials from a natural setting:<br>• Field permit number<br>• Name of the institution or relevant body that granted permission	
**Data Availability**<br><br>Authors are required to make all data underlying the findings described fully available, without restriction, and from the time of publication. PLOS allows rare exceptions to address legal and ethical concerns. See the PLOS Data Policy and FAQ for detailed information.	Yes - all data are fully available without restriction

Powered by Editorial Manager® and ProduXion Manager® from Aries Systems Corporation

A Data Availability Statement describing where the data can be found is required at submission. Your answers to this question constitute the Data Availability Statement and **will be published in the article**, if accepted.<br><br>**Important:** Stating 'data available on request from the author' is not sufficient. If your data are only available upon request, select 'No' for the first question and explain your exceptional situation in the text box.<br><br>Do the authors confirm that all data underlying the findings described in their manuscript are fully available without restriction?	
**Describe where the data may be found in full sentences. If you are copying our sample text, replace any instances of XXX with the appropriate details.**<br><br>• If the data are **held or will be held in a public repository**, include URLs, accession numbers or DOIs. If this information will only be available after acceptance, indicate this by ticking the box below. For example: *All XXX files are available from the XXX database (accession number(s) XXX, XXX.).*<br>• If the data are all contained **within the manuscript and/or Supporting Information files**, enter the following: *All relevant data are within the manuscript and its Supporting Information files.*<br>• If neither of these applies but you are able to provide **details of access elsewhere**, with or without limitations, please do so. For example:<br><br>*Data cannot be shared publicly because of [XXX]. Data are available from the XXX Institutional Data Access / Ethics Committee (contact via XXX) for researchers who meet the criteria for access to confidential data.*<br><br>*The data underlying the results presented in the study are available from (include the name of the third party*	Data is provided in the supplemental material. Upon acceptance, short read sequences will be deposited in NCBI.

and contact information or URL).<br>• This text is appropriate if the data are owned by a third party and authors do not have permission to share the data.<br><br>* typeset	
Additional data availability information:	

Manuscript

Click here to access/download;Manuscript;CAJA_Diet analysis_PlosOne_Final.docx

Fecal DNA metabarcoding helps characterize Canada jay diet and confirms the reliance of stored food for winter survival and breeding

Alex O. Sutton[1,2], Dan Strickland[3], Jacob Lachapelle[1], Robert G. Young[1], Robert Hanner[1], Daniel F. Brunton[4], Jeffrey H. Skevington[5], Nikole E. Freeman[1,2,6], D. Ryan Norris[1]

[1] Department of Integrative Biology, University of Guelph, Guelph, ON, CA

[2] School of Natural Sciences, Bangor University, Bangor, Wales, UK

[3] 1063 Oxtongue Lake Road, Dwight, ON, CA

[4] Beaty Centre for Species Discovery and Botany Section, Canadian Museum of Nature, Ottawa, ON, CA

[5] Carleton University, Ottawa, ON, CA

[6] Division of Biology, Kansas State University, Manhattan, KS, USA

Key words: diet analysis, food hoarding, stomach content analysis, winter breeding

Running Head: Canada jay diet

Abstract

Accurately determining the diet of wild animals can be challenging if food items are small, visible only briefly, or are rendered visually unidentifiable in the digestive system. In some food caching species, an additional challenge is determining whether consumed diet items had been previously stored or are fresh. The Canada jay (*Perisoreus canadensis*) is a generalist resident of North American boreal and subalpine forests with anatomical and behavioural adaptations allowing it to make thousands of arboreal food caches in summer and fall that are presumably responsible for its high winter survival and propensity to begin breeding in the late winter. We first used DNA fecal metabarcoding to obtain novel information on nestling diet and then consolidated this information with all published and additional unpublished diet information throughout the year and across life stages. Using natural history information of diet items identified, we then estimated the extent to which food consumed by adults in the winter and nestlings in the late winter/early spring was 'likely cached' or 'fresh'. In total, our dataset included 662 observations of food items consumed by Canada jays. Of the 87 food items consumed by adults in the winter, 39% were classified as 'likely recovered' caches and 6% were deemed to be 'likely fresh'. For nestlings, 29% of 125 food items identified to species were 'likely recovered' and 38% were 'likely fresh'. Our results support both the indispensability of cached food for Canada jay winter survival and previous suggestions that cached food is important for late winter/early spring breeding. Our work highlights the value of combining metabarcoding and natural history observations to determine the temporal origins of food items consumed by individuals, which, for the Canada jay, will be integral for understanding how diet influences individual success and population dynamics.

Introduction

While diet is closely linked to growth and individual performance (1–3), several factors frequently limit our ability to identify what free-living animals are consuming (4). It is often difficult to observe what is eaten or fed to young either because individual food items are too small to identify, have been manipulated in ways that limit the ability of an observer to identify a food item (e.g., multiple food items combined into a single food bolus), or are hidden by cryptic behaviour of adults (e.g., nesting high in a tree, concealed nest). Some of these difficulties can be overcome through the identification and quantification of stomach contents but this approach suffers from limitations of its own, including the need to collect samples before they are digested (5–7), the requirement that animals must either be found dead or sacrificed to obtain samples, and the inability to identify or quantify soft-tissue foods such as fungi, vertebrate flesh, or even some plant material (8).

Technological advances have overcome at least a few of problems associated with estimating diet. In some circumstances, high-speed photography and/or the use of drones have enhanced our ability to identify food items being consumed by animals (9) and stable isotope analysis can be used to quantify diet composition during the period of tissue growth (6,10). Limitations nevertheless persist; videography, for example, will likely never permit certain identification of arthropods being delivered in a compact bolus directly into the mouth of a nestling passerine and, unless the diet of an animal is simple (few potential sources), stable isotope analysis is typically restricted to identifying only major food groups (e.g., invertebrates vs. vertebrates vs. plants). The problems associated with food item identification can be further complicated in species that cache food (11). In these, it may not always be clear whether individuals are consuming food that is currently available (i.e., fresh) or food items that were

stored weeks or months earlier, raising the possibility that a completely different suite of food resources had been available compared to when the food was consumed.

All these challenges have a bearing on attempts to elucidate the diet of the Canada jay (*Perisoreus canadensis*), an iconic, sedentary food-storing species of North American boreal and subalpine forests. Canada jays regularly store food in trees, well above eventual snow levels, using sticky saliva from unusually large salivary glands (12,13) to fasten individual food items under bark scales or tufts of lichens. Food storage is conspicuous late-summer and fall behaviour in Canada jays and, presumably as a result, adults have extremely high (> 90%) winter survival, at least at the southern edge of their range (14,15). However, despite the high likelihood that the recovery of cached food explains the high winter survival and territorial fidelity of Canada jays, we have very few data on what sustains them throughout the winter and what proportion of this food may be from cached stores. A further reason why such information is of particular interest is that the main foods Canada jays are known to consume (arthropods; berries; fleshy fungi; and vertebrate flesh; 22) are all highly perishable. It seems questionable that such foods could reliably retain sufficient nutritional value from summer storage to the onset of sub-freezing (rot-arresting) temperatures in fall to account for the high winter survival of Canada jays (14). Strickland et al. (16) provided some experimental evidence that the volatile anti-microbial resins of conifers, particularly spruce (*Picea* spp.), may account for reduced degradation of perishable Canada jay caches but, ideally, one would like some assurance that the high winter jay survival is not attributable instead to some unsuspected non-perishable food, as in nutcrackers (*Nucifraga* spp.; 17).

Unequivocally characterizing the diet of the Canada jay and determining the importance of stored food is even more difficult in the breeding season. With no obvious sources of fresh

food, Canada jay females form and lay clutches as early as mid-February in Algonquin Park, Ontario (18) and raise offspring to fledging by late April-early May when, at least historically, the ground may still be snow covered, the lakes are still frozen, green-up has barely begun, and fewer than 10% of migratory passerines have returned let alone begun nesting themselves (19). Experimental evidence supports the inference that stored food facilitates early breeding in Canada jays (20), but concrete evidence in the form of positively identified natural food items that could only have been stored when recovered and fed to nestlings does not exist.

We had three primary aims in this study. First, we used dietary DNA (dDNA) metabarcoding (21) to expand our knowledge of the diet of nestling Canada jays. Second, we characterized, as completely as possible, the nestling and adult diets of the Canada jay, identifying, if possible, any significant variation between them and, for adults, any seasonal differences (i.e., winter versus "non-winter"). Third, we assessed the extent to which items identified in the diets of nestlings and winter adults had likely been cached before being recovered by the adults and then consumed or fed to nestlings. In doing so, we also discuss the practicality and limitations of using fecal dDNA metabarcoding for determining previously difficult-to-obtain information on animal diets.

Methods

Study Species and Field Site

Canada jays are year-round residents of North American boreal and subalpine forests (22), ranging from the tree line in the north to as far south as Arizona. We collected diet data as part of an ongoing long-term study begun in 1964 of Canada jay demography and behavioral ecology in Algonquin Provincial Park (APP) near the southern edge of its range in Ontario, Canada in the

transition zone between the Great Lakes-St. Lawrence deciduous hardwood forest and the boreal forest (16). Nest building begins in mid- to late-February with clutches initiated as early as February 22. Nestlings typically hatch in mid- to late-March under still wintry conditions and remain in the nest for approximately 23 days before fledging (22).

Collection of nestling fecal samples for metabarcoding

Since the only information on the Canada Jay nestling diet was from the contents of ten nestling stomachs or castings summarized by Strickland and Ouellet (22), our first aim was to improve our understanding of the diet through fecal dDNA metabarcoding. From 2015 to 2017, we collected 20 fecal sacs from Canada jay nestlings in APP during April and May. Fourteen fecal sacs were collected opportunistically while nestlings were removed from the nest to be banded when they were approximately 11 days old (1,15). Each fecal sac was collected from a unique individual, although six fecal sacs came from the same nest. These six fecal sacs were collected from a single nest on the day that the young fledged, but the individual that each fecal sac came from could not be determined with certainty. Thus, at least one nestling, likely more, was represented more than once. Cumulatively, the fecal sacs came from 8 separate nests and at least 11 nestlings. Upon collection, fecal sacs were immediately stored in a 50mL falcon tube filled with 100% ethanol and stored in a freezer within 12 hrs of being collected before being transferred to a -20°C freezer until processing.

DNA extraction, amplification, and sequencing

DNA was extracted from the fecal sacs for amplification following methods outlined by Prosser and Hebert (23). Polymerase chain reaction (PCR) was used to amplify three molecular regions:

a 157 bp region of the animal COI-5P (Cytochrome c Oxidase I) barcode (primer set ZBJ-ArtF1c-ion and ZBJ-Art2-ion; Zeale et al. 2011), a 350 bp region of the ITS2 (Internal Transcribed Spacer) region for fungi (primer set ITS3-M13ion and ITS4-M13ion; 23), and a 163 bp region of the rbcLa (Ribulose 1,5-Biphosphate Carboxylase) gene for plants (primer set rbcLaF-M13ion and MrbcL 163-R1-M13ion; 23, 25).

The choices of molecular markers for DNA metabarcoding followed Prosser and Hebert (23). Three molecular gene regions were chosen to provide information on Canada jay fungal (ITS2), botanical (rbcLa), and animal (COI) diet components. The rbcLa molecular region was selected as it is readily amplifiable across a large diversity of plant life and for its utility in placing specimens to family and/or genus taxonomic levels (26). The ITS region was selected as it has been shown to provide specimen identifications to species for a diverse number of fungi (27). The COI molecular region has been selected as a well established "DNA barcode" standard region by the Consortium for the Barcode of Life, and it has been well established as an effective barcode across all animal taxa of interest in this study (28,29). The selection of these three molecular regions is further supported through the large number of publicly available sequence records against which we can compare our results (as opposed to other molecular marker options). Lowest taxonomic level identification of the sequence reads was supported through barcode gap analyses using the target molecular region and available public data. The amplification and sequencing process was successful for only 15 of the 20 fecal sacs collected and all subsequent results are based on these 15 fecal sacs[illegible]

Bioinformatics

Sequence reads were filtered to include only sequences with a minimum length of 100 bp and a quality value (QV) of 2[illegible]rimers were trimmed from each read and filtered again by a minimum

size of 100 bp. Reads were collapsed based on identical sequences, which were then compared to custom databases using the Basic Local Alignment Search Tool (BLAST) algorithm. The custom databases included a land plant rbcLa database, a fungal ITS2 database, and an animal COI database obtained from the Barcode of Life Datasystem (BOLD), and Genbank. We filtered the BLAST output to exclude hits with less than 95% identity across at least 100 bp of the query sequence. The data were further filtered to exclude sequences with fewer than 100 reads to eliminate degraded sequence which may be present from other sources such as secondary predation or environmental contamination.

To support taxonomic identifications, the discriminatory ability of the amplified molecular regions was assessed. A barcode gap analysis was used to ascertain if the molecular region, using all publicly available data, was able to reliably place a sequence to a taxonomic group. To have a barcode gap there must be less variation within a group then there is between the members of that group and all other members outside the group (30). To provide the most robust analysis of this gap, distances between all elements were calculated and the highest difference between members within the target group is compared to the smallest distance between members of the target group and all other members outside the target group. COI molecular region BLAST identifications were pulled back to genus if no gap was obtained from our gap analyses. With the rbcLa molecular region, taxa with no barcode gap using species level taxonomic identifications were tested between genera. If there was no apparent gap at the level of genus taxonomic identifications were pulled back to Family. Placing a higher-level taxonomic identification at genus for animals using a segment of the COI and family for plants using a segment of the rbcLa follows established methods (25).

To assess if a barcode gap exists for the BLAST identifications, higher taxonomic levels (family for plants and either family or genus for animals depending on the manageable size of the data set and the number of BLAST identified species from the same genus or family) were used to obtain sequence data sets from the BOLD system. Sequences were aligned (MAFFT: 31) and trimmed to target region using MEGA (32). Sequences were removed from further analysis if they had greater than 2% unknown nucleotides, or if they had greater than 12 gap characters (‘-’) at either the 3’ or 5’ ends of the sequences. A simple distance matrix was constructed using the R package Ape and the dist.dna matrix function (33). These matrices were then used to obtain the maximum within species genetic variation and the minimum genetic distance between species of the same genus (see Supplemental Data).

Compilation of a comprehensive adult and nestling diet dataset

To facilitate a more thorough analysis and comparison of adult and nestling Canada jay diets, we combined the nestling food items identified in the metabarcoding outlined above, our own and published direct observations of items taken by foraging jays across their continental range, and all items visually identified by us or others in stomach contents of both nestlings and adults, (including those identified in the 1980s by experts of the then Biosystematics Research Institute (BRI) of Canada’s federal Department of Agriculture in Ottawa) into a single dataset (Supplementary Table S1). The BRI identifications (summarized by 22 but otherwise never published) included items from the stomachs and regurgitated pellets of 10 APP nestlings and from 18 stomachs of adults inadvertently killed in traps set for furbearers in northern Ontario. The entire dataset included 662 items and was partitioned into three “food groups”: (1) arthropods, (2) “plants” - including vascular plants, fungi, and slime moulds, and (3) vertebrate flesh - including from carcasses and nestling birds or small mammals killed by jays themselves.

These items were further partitioned according to life stage (nestling *vs* "adult"), observation method (direct observation *vs* stomach contents *vs* barcoding), and time of year ("winter" – Nov. 1 to Mar. 31 *vs* "non-winter" – May 1 to Oct. 31). We supplemented these data with findings from two previously published papers: an stable-isotope analysis of adult and nestling diet (10) and an observational study of adults in Alaska (34) before numerically and graphically summarizing the results (Figure 1) and using them to draw inferences about the similarities and differences between adult and nestling diets.

Determination of cached vs "fresh" origins of food items from nestling and adult winter diets

Based on natural history considerations, we sought to determine the extent to which food items identified in nestling fecal sacs through dDNA metabarcoding or stomach contents or in the stomach contents of adults sampled in winter (Nov. 1-Mar. 31) were likely cached or likely "fresh" (i.e., non-cached). To do this we assigned all food items to one of four categories: "unknown" (when nothing was known about the source organism's natural history, when we were confident the item had been misidentified, or when it had not been identified to species); "either possible" (when the food items were present and accessible year round); "likely fresh" (if the food items were widely available as non-cached items at the time when they were detected in fecal sacs or stomach contents and especially if they were unavailable in the preceding summer/fall food storage season); or "likely cached". We deemed *a priori* that the "likely cached" designation would be justified if the food item satisfied one or more of the following four criteria: (1) The food item was from a migratory species known to be entirely absent from the location where, and on the date when, the food observation was made (e.g., finding Monarch butterfly (*Danaus plexippus*) DNA in a nestling fecal sac in April), (2) The identified taxon was in a life stage known not to be present on the date of the food observation (e.g., the adult form of

an insect that overwinters as an egg or larva), (3) All individuals of the source taxon are known to be underground when and where the food observation was made (e.g., a hibernating jumping mouse, *Zapodidae*), and (4) Snow depth on the date of the food observation would have precluded access to the item if it were still in its source location (e.g., a cranberry, *Vaccinium sp.*, still on the parent plant, a few cm from the substrate). To inform our application of criterion 4, we obtained historical snow depth records from Environment and Climate Change Canada weather stations located close to the sampling location (https://climate.weather.gc.ca/historical_data/search_historic_data_e.html) and, whenever possible, supplemented them with more detailed records from the then Ontario Ministry of Natural Resources.

The final determinations of "likely cached vs likely fresh vs either possible" status was made by D.F.B. and J.H.S., using their respective botanical and entomological expertise and their specific familiarity with the natural history and phenology of northern Ontario (where we obtained most of our winter adult stomachs) and of APP (where we obtained all of our nestling fecal barcoding and stomach-contents results).

Results

Summary of nestling diet items identified through DNA fecal metabarcoding

Of the 147 unique taxa accounted for in nestling fecal sacs, 2% (3/147) were vertebrates (wood frog; *Lithobates sylvaticus* and a shrew; *Sorex cinereus*), 74% (109/147) were arthropods, and the remaining 24% (35/147) were plants. Overall, *Araneae* (30 spiders) and *Lepidoptera* (44 moths and butterflies) represented a majority total food items detected (n = 147; Figure 1), but the most common food items detected across fecal sacs were *Ericales* plants (heather and allies;

most commonly Blueberries in our study), which were detected in 51% of the fecal sacs (Supplementary Table S1, Figure 1).

Summary description of consolidated dataset

Figure 1 summarizes and compares results across methodologies based on data in Table S1, as well as two recently published studies (10, 34). Table S1 lists 662 observations of Canada jay diet items that were either reported in the literature (excluding observations in 34), observed by us personally, or reported to us by others. Of these, 121 (18 %) were “direct observations” (i.e., cases where one or more jays were seen consuming a single plant or animal species), 147 (22%) were species identified in our fecal barcoding analysis (see below), and 394 (60%) were items identified in stomach contents including 65 items from ten nestling stomachs. Within each of these three “observation-type” categories, we further partitioned observations into three broad food types (arthropods, plants, and vertebrates). Of the 121 direct observations, for example, 20 (17%) involved arthropods, 23 (19%) involved plant material, and 78 (64%) involved vertebrates. The direct observations were unique in that, for two of the direct observation sub-categories (plants and vertebrates), a further useful partitioning was possible. Eight of the 23 observations of “plants” being consumed by Canada jays involved fungi or slime moulds, hinting at the possibly under-appreciated importance of these taxa in Canada jay diets. Soft-bodied fungi cannot be identified in semi-digested stomach contents. Nor, for technical reasons, could they be detected through dDNA metabarcoding of nestling fecal sacs in our study (because amplification of ITS2 was not successful) or through stable isotope analyses. In a similar manner, our compilation of direct observations of the consumption of vertebrates (n = 75; Table S1) showed that 8% (n =6) involved vertebrate eggs, 45% (n = 34) involved carrion, and 47% (n = 35) involved live prey. Most live prey were small mammals or nestlings of other bird species but

recently fledged birds were also observed to be consumed, including two that were first struck and disabled in flight (35). Overall, the nestling diet, as revealed by the combined results of our fecal dDNA metabarcoding and stomach-contents analyses, had a greater proportion of arthropods than the winter-adult diet (80% vs 49%), and a correspondingly lower proportion of "plant" items (18% vs 36%) and vertebrate items (2% vs 15%).

Cached vs. fresh food determinations for diet items

Figure 2 and Table S2 summarize the 'cached-versus-fresh' data obtained from our nestling fecal barcoding analysis and from the stomach contents from 18 winter adults and 10 nestling stomachs and castings. Of the 194 winter adult food items and the 212 nestling diet items, 45% (n= 87) and 59% (n= 125), respectively, were identified to species with sufficiently well-known natural histories that we were able to further categorize the items as "likely cached", "likely fresh", or "either possible". In the subset of winter-adult food items (n = 87), we judged that 39% were "likely cached", 55% could have been cached or fresh, and only 6% were "likely fresh". In the corresponding subset of nestling food items (n = 125), we found a different pattern: 28% were "likely cached", 34% could have been either cached or fresh, and 38% were "likely fresh". "Likely-cached" food was most likely to be plant items (e.g. berries) for both winter-adult diets (57%; 32/56 total items) and nestling diets (67%; 22/33 total items). In contrast, vertebrate tissue was the least likely diet food category to be "likely-cached" but this may reasonably be attributed to the fact that all the vertebrate items we found in winter stomachs were from species, particularly small mammals, that are active year-round and we could therefore not assess whether the items represented cached or fresh food. Additionally, despite the relatively small proportion of "likely cached" food items in the nestling diet, it is noteworthy that, 66% (10/15)

of nestling fecal sacs contained at least one “likely cached” food item, supporting the importance of cached food in the nestling diet of Canada jays.

Discussion

The value of dietary DNA metabarcoding to investigate animal diets

We used DNA metabarcoding to significantly improve our knowledge of the nestling diet of Canada jays and to strengthen evidence that stored food is important in that diet. This is a relatively new method for analyzing diets and a careful assessment of its strengths and weaknesses may therefore be useful (36). First, it does have limitations; it requires DNA reference libraries of potential food items (37,38) and it can be prone to misidentifications when those libraries are incomplete or PCR primers are not optimized for the taxa of interest. Second, in combination with accurate natural-history and environmental (e.g., snow-depth) information, it largely duplicates the ability of stomach contents analysis to plausibly assess whether or not a given item was “likely cached”. Moreover, at least in principle, stomach contents analysis would outperform barcoding if it found exoskeletal fragments corresponding to an arthropod’s life-history stage that was unavailable in nature on the date when the fragments were found; dDNA metabarcoding cannot aspire to such determinations because it can determine only species identity, not its life-history stage. As a practical matter, however, we found no examples in our study where this potential handicap detracted from dDNA metabarcoding’s otherwise superior ability to identify taxa ingested by Canada jays and to determine which items had been “likely cached”. Not only does metabarcoding permit the identification of soft-tissue or small food items that are easily missed by stomach contents analysis (39–41), but also, compared to stomach contents analysis, it allows far more samples to be collected, far more easily, and importantly,

without sacrificing individuals. In just three seasons (2015 - 2017) of measuring nestlings, we were able to double the number of identified nestling diet items that we had obtained during the previous 40 years through chance acquisitions of stomach contents and castings. A further advantageous feature is that the technique could be extended to more accurately assess the diets of not just nestling Canada jays, but also those of adults, including the winter diets that are of greatest interest. We have no doubt that it would be eminently feasible to capture winter adults and safely hold them in a suitably appointed small cage until they produced a fecal sample. By the same reasoning, it should be possible, in principle, to use fecal dDNA metabarcoding far beyond the limited use we have made of it here and we encourage others to do so.

Characterization of the Canada jay diet by season and life history stage

The four methods we used to elucidate diet ("direct observations", stomach contents analysis, stable isotope analysis, and fecal metabarcoding; Figure 1) all indicated that Canada jays consume arthropods, "plants" (almost entirely berries), and vertebrates, but each method provided different estimates for the overall portion of each food type in the diet. For example, it is likely that the high proportions of vertebrate flesh suggested by our compilation of "direct observations" (65%) and stable isotope analysis of food-dependent APP juveniles (72%; 10) are both overestimates. Stable isotope analyses relied on a fractionation factor derived from an unrelated species (Yellow-rumped warbler; *Setophaga coronata*), which could have resulted in a mischaracterization of diet groups and their contributions to Canada jay diet (10). Similarly, our compilation of direct observations almost certainly exaggerates the importance of vertebrate flesh in the diet (and minimizes that of arthropods) because observers are more likely to report consumption of familiar vertebrate taxa than tiny, quickly ingested invertebrates. In contrast, the much lower proportion of vertebrates (24%) reported in a direct-observation study in Denali,

Alaska (34) is probably more accurate because it is based on a much larger sample and because the observers made concerted efforts to record all food acquisitions by focal individuals. However, we also note that differences in diet composition between the APP and Denali population could simply be due to differences in prey composition or climate. Despite potential bias associated with direct observations, one major advantage of this approach is that it is able to register the consumption of fleshy fungi (Figure 3a) and slime moulds (Figure 3b), to distinguish between incidents of scavenging versus actual predation (Figure 3c, d), and to record unusual foraging methods (e.g. flycatching; wading in shallow water to capture tadpoles (42) or larval salamanders (43).

The only presently available data permitting a comparison of winter and "non-winter" (May 1 – Oct. 31) Canada jay diets are from stomach contents and stomach castings (Figure 3e). Given the frigid realities of boreal forest winters, it is noteworthy that arthropod and plant items (Figure 3f) accounted for 49% and 36%, respectively of items identified in winter-adult stomachs, compared to corresponding figures of 66% and 30% in non-winter stomachs.

In the Canada jay nestling period, arthropods accounted for even greater proportions of the items identified in nestling stomachs and feces (92% and 74%, respectively), possibly reflecting a particular preference in parents for protein-rich arthropods for their rapidly growing nestlings, as has been found in many other bird species (44,45). Caution is warranted, however, before concluding that arthropods are overwhelmingly important in the diet of Canada jay nestlings. Notably, dDNA metabarcoding cannot rule out the possibility of secondary predation. In their stable-isotope analysis of nestling tissues, Freeman et al. (1) found that only 14% of the nestling diet was attributable to invertebrates whereas 51% of the food given to nestlings was believed to be of vertebrate or human-food origin. Notwithstanding methodological issues with

stable isotope analysis noted above, this may well be the more meaningful representation of the nestling diet because stable isotopes give an estimate of volume of each food class whereas the analysis of nestling stomach contents or feces yields numbers of separate, identifiable food items or taxa contained in each stomach or fecal sac without an indication of the proportion of each food item. Consider that, if a Canada jay consumed three individuals of each of five species of arthropods and an amount of flesh and identifiable small bones from a single mouse species with an equal nutritional value and mass to that of the arthropods, stomach-contents analysis would potentially indicate, correctly, that the jay had consumed 15 arthropod items and one vertebrate item, dDNA metabarcoding would indicate that the diet included 5 arthropod taxa and one vertebrate, and stable-isotope analysis would indicate, also correctly, that arthropods and vertebrates contributed equally to the jay's nutrition. Despite these persisting uncertainties in how best to characterize Canada jay diets, we believe our results make clear that nestling and adult diets are at least roughly similar and that, even in winter, Canada jays somehow have access to berries and arthropods that would seem to be primarily, if not exclusively, available only in the snow-free part of the year.

Contribution of stored food to adult-winter and nestling diets

By combining the identification to species level of many items found in the stomachs and feces of winter adults and nestlings with natural history information about those food species, we deemed that 39% of winter-adult items had likely been cached and only 6% were likely found as "fresh items". By contrast, we deemed that only 28% of items detected in nestling stomachs and feces had likely been cached and at least 38% had been consumed as fresh items. We attribute the much greater proportion of apparently fresh items in the nestling diet to the fact that Canada jay nestling period in our study area typically straddles the disappearance of snow cover in mid-

to late-April and, in our case, half of our nestling stomachs and fecal sacs were collected considerably later than that, even as late as May 19 (from a replacement nest). Derbyshire et al. (20) previously pointed out that once snow cover has disappeared, Canada jay parents forage for their nestlings on the forest floor where food storage has never been observed and Swift et al. (34) similarly reported that Canada jays in Denali, Alaska "responded to a record-setting warm spring by directing their foraging efforts away from cache recovery and towards the emergence of fresh food". Together these results lend further support to the suggestion by Strickland and Ouellet (2020) that food storage is the key behaviour in Canada jays permitting not only their high winter survival and territorial fidelity, but also their high nesting success (thanks to the use of stored food as critical "emergency food" in late springs or during the not unusual snow and ice storms that occur in the late-winter Canada jay nesting season).

Our work demonstrates the value of combining methods (e.g., direct observation, stomach contents, and DNA metabarcoding) when assessing the composition of the Canada Jay's nestling and adult diets. We believe a similar approach could be applied to other free-living species to advance our knowledge of the diet of wild birds. Such knowledge is integral to understanding how individuals interact with their environments and how diet, and subsequently individual performance, may change across habitats and with climate change (46).

Acknowledgements

We would like to thank countless field assistants who collected diet information and demographic data from Canada jays in Algonquin Provincial Park. We would also like to thank NSERC, Ontario Parks, Friends of Algonquin Park, Wildlife Conservation Society Canada, and the Ministry of Natural Resources and Forestry for providing funding and logistical support for

our project. Finally, we would like to thank the Ontario Ministry of Northern Development, Mines, Natural Resources and Forestry for providing snow depth data.

Literature Cited

1. Freeman NE, Norris DR, Sutton AO, Newman AEM. Raising young with limited resources: supplementation improves body condition and advances fledging of Canada Jays. Ecology. 2020;101(1):e02909.

2. Kearney SP, Porensky LM, Augustine DJ, Derner JD, Gao F. Predicting spatial-temporal patterns of diet quality and large herbivore performance using satellite time series. Ecological Applications. 2022;32(2):e2503.

3. Pascacio-Villafán C, Righini N, Nestel D, Birke A, Guillén L, Aluja M. Diet quality and conspecific larval density predict functional trait variation and performance in a polyphagous frugivorous fly. Functional Ecology. 2022;36(5):1163–76.

4. Robinson BG, Franke A, Derocher AE. Estimating nestling diet with cameras: quantifying uncertainty from unidentified food items. wbio. 2015 Dec;21(5):277–82.

5. Ralph CP, Nagata SE, Ralph CJ. Analysis of Droppings to Describe Diets of Small Birds. Journal of Field Ornithology. 1985;56(2):165–74.

6. Meckstroth AM, Miles AK, Chandra S. Diets of Introduced Predators Using Stable Isotopes and Stomach Contents. The Journal of Wildlife Management. 2007;71(7):2387–92.

7. Connan M, McQuaid CD, Bonnevie BT, Smale MJ, Cherel Y. Combined stomach content, lipid and stable isotope analyses reveal spatial and trophic partitioning among three

sympatric albatrosses from the Southern Ocean. Marine Ecology Progress Series. 2014;497:259–72.

8. Valentini A, Pompanon F, Taberlet P. DNA barcoding for ecologists. Trends in Ecology & Evolution. 2009 Feb 1;24(2):110–7.

9. Verkuil YI, Nicolaus M, Ubels R, Dietz MW, Samplonius JM, Galema A, et al. DNA metabarcoding quantifies the relative biomass of arthropod taxa in songbird diets: Validation with camera-recorded diets. Ecology and Evolution. 2022;12(5):e8881.

10. Freeman NE, Newman AEM, Sutton AO, Kyser TK, Norris DR. Causes and consequences of variation in diet composition of nestling Canada jays. Journal of Avian Biology. 2021;52(1).

11. Vander Wall SB. Food Hoarding in Animals. University of Chicago Press; 1990. 458 p.

12. Bock WJ. Salivary Glands in the Gray Jays (Perisoreus). The Auk. 1961;78(3):355–65.

13. Dow DD. The Role of Saliva in Food Storage by the Gray Jay. The Auk. 1965;82(2):139–54.

14. Norris DR, Flockhart DTT, Strickland D. Contrasting patterns of survival and dispersal in multiple habitats reveal an ecological trap in a food-caching bird. Oecologia. 2013 Nov;173(3):827–35.

15. Sutton AO, Strickland D, Freeman NE, Norris DR. Climate-driven carry-over effects negatively influence population growth rate in a food-caching boreal passerine. Global Change Biology. 2021;27(5):983–92.

16. Strickland D, Kielstra B, Ryan Norris D. Experimental evidence for a novel mechanism driving variation in habitat quality in a food-caching bird. Oecologia. 2011;167(4):943–50.

17. Tomback DF. Clark's Nutcracker (Nucifraga columbiana), version 1.0. Birds of the World. 2020; Available from: https://birdsoftheworld-org/bow/species/clanut/cur/introduction

18. Sechley TH, Strickland D, Norris DR. Causes and consequences of pre-laying weight gain in a food-caching bird that breeds in late winter. Journal of Avian Biology. 2014 Jan;45(1):85–93.

19. Tozer R. Birds of Algonquin Provincial Park. 2012.

20. Derbyshire R, Norris DR, Hobson KA, Strickland D. Isotopic spiking and food dye experiments provide evidence that nestling Canada Jays (Perisoreus canadensis) receive cached food from their parents. Can J Zool. 2019;97(4):368–75.

21. de Sousa LL, Silva SM, Xavier R. DNA metabarcoding in diet studies: Unveiling ecological aspects in aquatic and terrestrial ecosystems. Environmental DNA. 2019;1(3):199–214.

22. Strickland D, Ouellet HR. Canada Jay (Perisoreus canadensis). Rodewald PG, editor. Birds of the World. 2020; Available from: https:// birdsoftheworld-org./Species-Account/species/gryjay/introduction

23. Prosser SWJ, Hebert PDN. Rapid identification of the botanical and entomological sources of honey using DNA metabarcoding. Food Chemistry. 2017 Jan 1;214:183–91.

24. Zeale MRK, Butlin RK, Barker GLA, Lees DC, Jones G. Taxon-specific PCR for DNA barcoding arthropod prey in bat faeces. Molecular Ecology Resources. 2011;11(2):236–44.

25. CBOL. A DNA Barcode for Land Plants. Proceedings of the National Academy of Sciences of the United States of America. 2009;106(31):12794–7.

26. Kress WJ, Erickson DL. A Two-Locus Global DNA Barcode for Land Plants: The Coding rbcL Gene Complements the Non-Coding trnH-psbA Spacer Region. PLOS ONE. 2007 Jun 6;2(6):e508.

27. Schoch CL, Seifert KA, Huhndorf S, Robert V, Spouge JL, Levesque CA, et al. Nuclear ribosomal internal transcribed spacer (ITS) region as a universal DNA barcode marker for Fungi. Proceedings of the National Academy of Sciences. 2012 Apr 17;109(16):6241–6.

28. Ratnasingham S, Hebert PDN. bold: The Barcode of Life Data System (http://www.barcodinglife.org). Molecular Ecology Notes. 2007;7(3):355–64.

29. Virgilio M, Backeljau T, Nevado B, De Meyer M. Comparative performances of DNA barcoding across insect orders. BMC Bioinformatics. 2010 Apr 27;11(1):206.

30. Meyer CP, Paulay G. DNA Barcoding: Error Rates Based on Comprehensive Sampling. PLOS Biology. 2005 Nov 29;3(12):e422.

31. Katoh K, Standley DM. MAFFT Multiple Sequence Alignment Software Version 7: Improvements in Performance and Usability. Molecular Biology and Evolution. 2013 Apr 1;30(4):772–80.

32. Kumar S, Stecher G, Li M, Knyaz C, Tamura K. MEGA X: Molecular Evolutionary Genetics Analysis across Computing Platforms. Mol Biol Evol. 2018 Jun 1;35(6):1547–9.

33. Paradis E, Claude J, Strimmer K. APE: Analyses of Phylogenetics and Evolution in R language. Bioinformatics. 2004 Jan 22;20(2):289–90.

34. Swift KN, Williams EJ, Marzluff JM. An observational analysis of Canada Jay (Perisoreus canadensis) foraging and caching ecology in Denali National Park and Preserve, Alaska, USA. Can J Zool. 2022;100(2):133–46.

35. Fuirst M, McLeod J, Norris DR. Habitat preferences of adult Canada Jays (Perisoreus canadensis) during the post-breeding period in Algonquin Provincial Park, Ontario, Canada. Can J Zool. 2022;100(6):355–62.

36. Ando H, Mukai H, Komura T, Dewi T, Ando M, Isagi Y. Methodological trends and perspectives of animal dietary studies by noninvasive fecal DNA metabarcoding. Environmental DNA. 2020;2(4):391–406.

37. Taberlet P, Coissac E, Pompanon F, Brochmann C, Willerslev E. Towards next-generation biodiversity assessment using DNA metabarcoding. Molecular Ecology. 2012;21(8):2045–50.

38. De Barba M, Adams JR, Goldberg CS, Stansbury CR, Arias D, Cisneros R, et al. Molecular species identification for multiple carnivores. Conservation Genet Resour. 2014;6(4):821–4.

39. Deagle BE, Gales NJ, Evans K, Jarman SN, Robinson S, Trebilco R, et al. Studying Seabird Diet through Genetic Analysis of Faeces: A Case Study on Macaroni Penguins (Eudyptes chrysolophus). PLOS ONE. 2007;2(9):e831.

40. Zarzoso-Lacoste D, Bonnaud E, Corse E, Gilles A, Meglecz E, Costedoat C, et al. Improving morphological diet studies with molecular ecology: An application for invasive mammal predation on island birds. Biological Conservation. 2016;193:134–42.

41. Groen K, Beekenkamp S, de Iongh HH, Lesilau F, Chege M, Narisha L, et al. DNA metabarcoding illuminates the contribution of small and very small prey taxa to the diet of lions. Environmental DNA. *in press*. Available from: https://onlinelibrary.wiley.com/doi/abs/10.1002/edn3.457

42. Beiswenger, R.. Predation by gray jays on aggregating tadpoles of the boreal toad (*Bufo boreas*). Copeia 1981;2:459-460.

43. Murray MP, Pearl CA, Bury RB. Apparent Predation by Gray Jays, *Perisoreus canadensis*, on Long-toed Salamanders, *Ambystoma macrodactylum*, in the Oregon Cascade Range. The Canadian Field-Naturalist. 2005;119(2):291–2.

44. Wilman H, Belmaker J, Simpson J, de la Rosa C, Rivadeneira MM, Jetz W. EltonTraits 1.0: Species-level foraging attributes of the world's birds and mammals. Ecology. 2014;95(7):2027–2027.

45. Bowler DE, Heldbjerg H, Fox AD, de Jong M, Böhning-Gaese K. Long-term declines of European insectivorous bird populations and potential causes. Conservation Biology. 2019;33(5):1120–30.

46. Bookwalter J, Niyas AMM, Caballero-López B, Villari C, Marco-Tresserras J, Burgas A, et al. DNA metabarcoding Passerine bird feces at tree-line uncovers little intra- and inter-

species dietary overlap. Community Ecology. 2023; Available from: https://doi.org/10.1007/s42974-023-00148-4

Figures

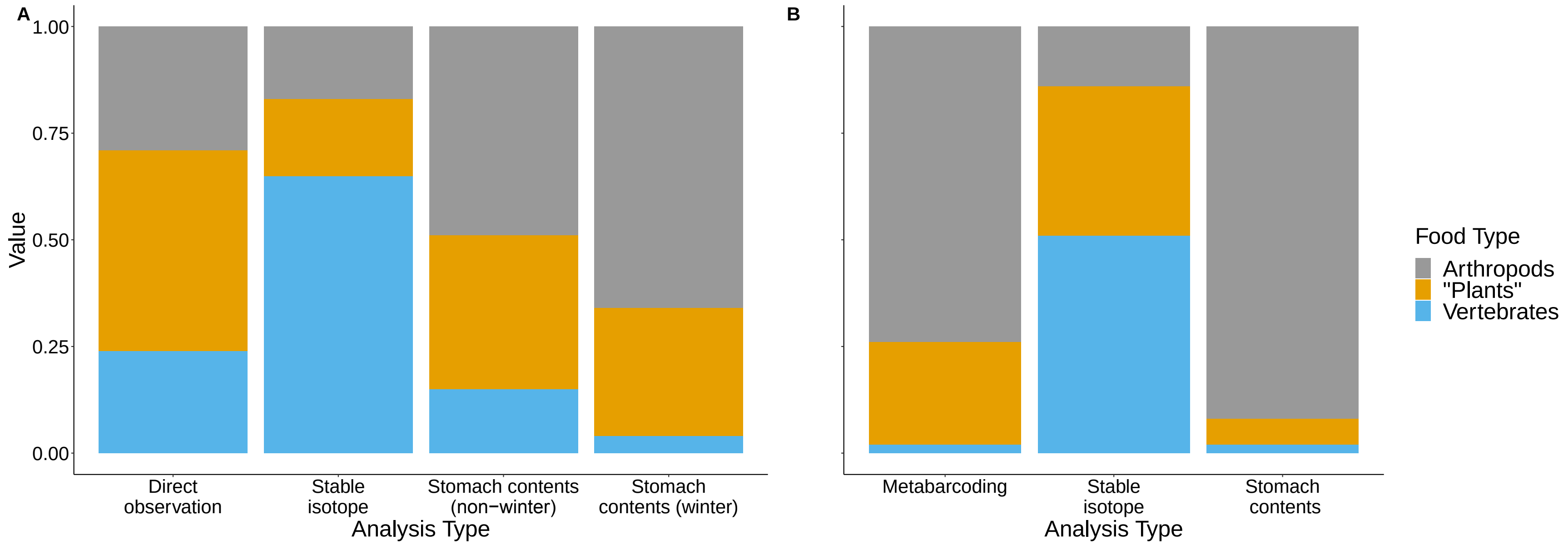

Figure 1. Proportion of diets composed of arthropods and other invertebrates (grey), 'plants' (orange, includes plants, fungi, and molds), and vertebrate tissue (blue). The table is divided into two sections; free flying individuals (adults and juveniles; A) and nestlings (B). We further divide our observations according to the method used to characterize diet composition (direct observation, stable isotope, and stomach contents) and present the estimated proportion of each of the three food groups in the overall diet. Additional information on identified diet items from each food type are available in Table S1.

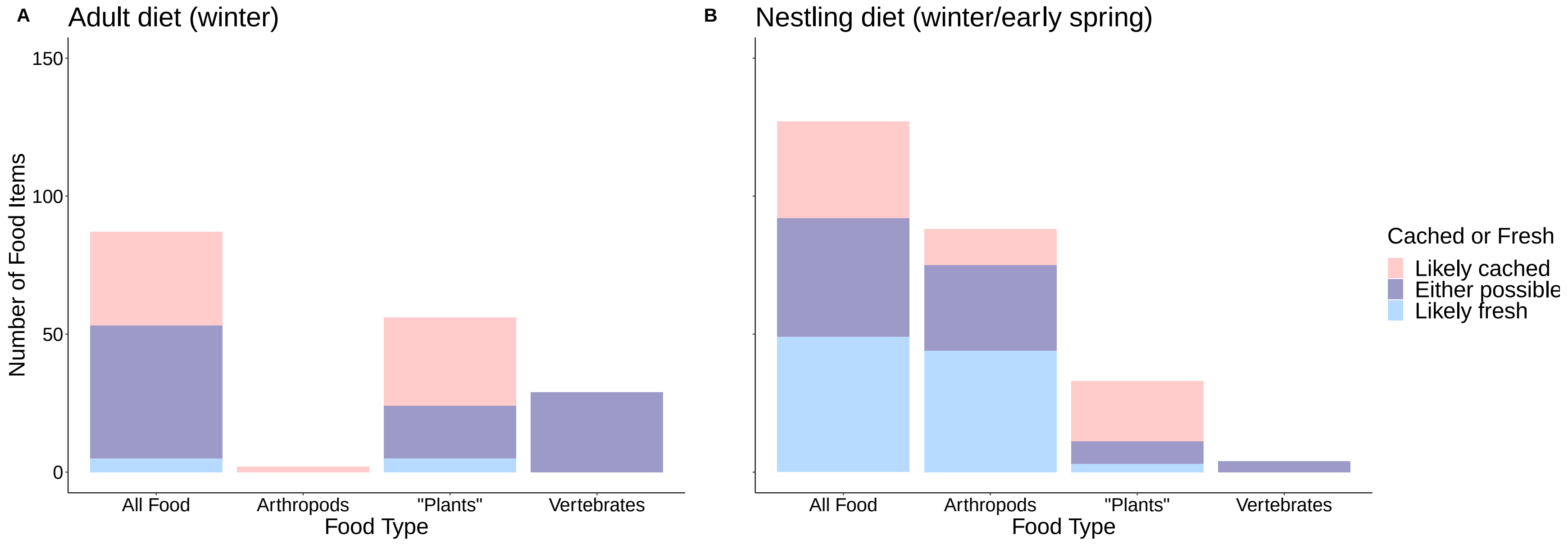

Figure 2. Characterization of food items as “likely cached” (light red), “either possible” (light purple), or “likely fresh” (light blue) for (A) adult Canada jay winter diet (n = 87 total observations) and (B) nestling diets using direct observations, stomach contents, and metabarcoding (n = 125 total observations). Allocation to the three categories was based on life history characteristics of food items and environmental conditions at time of observation or sample collection. The total number of food items in each of the three “cached” vs “fresh” categories are displayed under “All Food” and designations for the three major food groups (arthropods, plants, and vertebrate tissue) individually are also displayed.

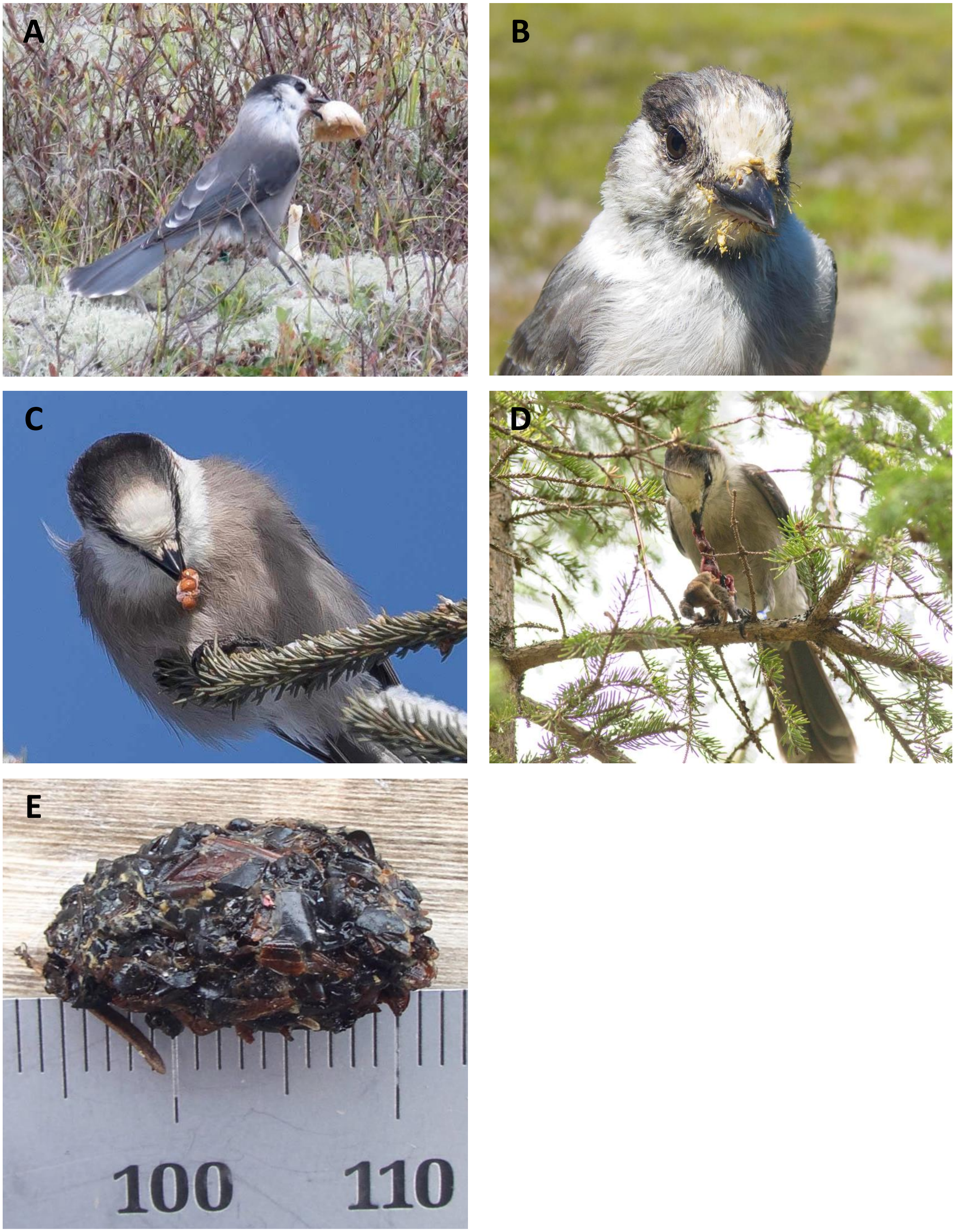
A
B
C
D
E
100
110

Figure 3. Direct observations made of Canada jays foraging on a wide range of food items. *A)* A Canada jay in Algonquin Provincial Park, Ontario feeding on *Amanita muscaria* (Photo by Langis Sirois, November 5, 2018), *B)* Canada jay with yellow residue around bill from recent consumption of the slime mould (*Fuligo septica*) on Vancouver Island, British Columbia (Photo by Dan Strickland, August 1, 2020), *C)* Canada jay regurgitating Choke Cherry (*Prunus virginiana*) seeds on a date (January 20, 2020) when cherries would be available only as cached items (Photo by Michael Runtz), *D)* Canada jay dismembering a recently caught shrew (*Sorex sp.*) in Algonquin Provincial Park, Ontario (Photo by Ann Brokelman, August 27, 2016), *E)* Canada jay stomach casting apparently consisting of arthropod exoskeletal fragments (Strathcona Provincial Park, Vancouver Island, British Columbia; Photo by Dan Strickland, June 26, 2021).

Click here to access/download
Supporting Information
CAJA_PLOSONE_Table S1_Formatted.docx

Click here to access/download
Supporting Information
CAJA_PLOSONE_Table S2_Formatted.docx
