# Supplementary material for: Fecal DNA metabarcoding helps characterize the Canada jay’s diet and confirms its reliance on stored food for winter survival and breeding
Source: PLoS One. 2024 Apr 24;19(4):e0300583. doi: 10.1371/journal.pone.0300583 (PMC11042713; doi:10.1371/journal.pone.0300583)
Supplement: Attachment — Submitted filename: CAJA Diet_Final_Response to Reviewer_final.docx [file pone.0300583.s011.docx]

**We thank the reviewers for their comments on our revised manuscript. Our responses to their comments are highlighted in the manuscript in red and bolded below for clarity.**

Reviewer #2: My main concerns have been addressed and I am satisfied with the answers and corrections.
Methods are now described in detail.
One note is that if you were following standard kits (i.e. for DNA extraction) and followed manufacturers instructions then you can skip giving all the details and just say "we used this-and-that kit following manufacturers instructions".

**Author response: Thank you for your suggestion. We have left the description of our manual extraction methods in the manuscript because it describes the methods followed throughout the analysis.**

Small comments:

general: Degrees symbol doesn't appear well in the PDF. To be fixed in formatting.

**Author response: As suggested, we have made sure all degree symbols are now being displayed appropriately.**

general: Sometimes you write "COI" and sometimes "COI-5P". I'm not sure what the difference is. Usually "COI" is sufficient.

**Author response: There is a difference between COI and COI-5P. For consistency and to reflect the fact we used the COI-5P, we have made sure all mention of COI throughout the manuscript has been changed to COI-5P.**

Line 126-127: Thanks for clarifying. In this case you can just write that it was stored in -20 degrees within 12hrs of collection and until further processing.

**Author response: As suggested, we have revised the text to state this.**

Line 130: You should write full name of the region first - Cytochrome c Oxidase I - and abbreviation in parentheses. Also only "c" should be italicised. Check it throughout the text.

**Author response: As suggested, we have made sure to write the full name first and only italicize the “c”.**

Line 131: Same with ITS. First write the full name of the fragment and then add abbreviation in parentheses.

**Author response: As suggested, we have made sure to write the full name first.**

Line 132: same

**Author response: As suggested, we have made sure to write the full name first.**

Line 224: Thanks for clarifying what the distance metric is. Now I think that "simple" is necessary. This is not a precise word. Just delete it and rephrase.

**Author response: As suggested, we have removed the word “simple” and revised the sentence to describe the matrix that was created. The revised text now reads: “A distance matrix (the proportion or the number of sites that differ between each pair of aligned sequences) was constructed with the R package Ape (Ver. 4.1) dist.dna() matrix function (35).” (lines 224 – 226)**

Reviewer #3: I want to commend the authors for the effort they put in to improve the manuscript and the attention they paid to reviewers’ comments. The authors made major changes to the manuscript that improved the value of the manuscript considerably. I like to recommend that the authors also consider the UNITE database for fungal sequence identification in possible future studies using DNA metabarcoding. I have noticed a few minor errors in the methods section:

Line 173: 10 µM (not lM) primer, 0.0625 μL of 10 mM dNTP (KAPA Biosystems), 0.060 µL (not lL) of 5U/μL PlatinumTaq

**Author response: Thank you for catching these mistakes. We have revised the text to correct these errors.**

Line 175: 175 reactions consisted of 94 °C for 5 min, 40–60 cycles (40 for ITS2, 60 for rbcLa (not A) and COI)

**Author response: We now have removed the [] from the sentence.**

Reviewer #4: I am very happy with the answers and changes provided by the authors.
I think the manuscript is now ready to be accepted.

I have only two very minor remarks:

- Lines 129-145: the paragraph on the selection of the markers should go with the PCR section, not before the DNA extraction. DNA extraction and purification happen before PCR, and they should be reported in this order.

**Author response: As suggested, we have moved this paragraph to be below our description of the PCR method used and now appears on lines 177 – 187.**

- Check the whole manuscript for the use of the degree symbol °C. Not sure what symbol was used instead, but it really looks weird.
**Author response: We have made sure all uses of °C are now consistently formatted.**

Great work, looking forward to see this published! Congratulations!

**Author response: Thank you for the kind words and for your helpful comment on previous versions of the MS.**
